# Template-Based Modelling of the Structure of Fungal Effector Proteins

**DOI:** 10.1007/s12033-023-00703-4

**Published:** 2023-03-20

**Authors:** Lina Rozano, Darcy A. B. Jones, James K. Hane, Ricardo L. Mancera

**Affiliations:** 1 Curtin Medical School, Curtin Health Innovation Research Institute, GPO Box U1987, Perth, WA 6845 Australia; 2 Centre for Crop and Disease Management, School of Molecular and Life Sciences, Curtin University, GPO Box U1987, Perth, WA 6845 Australia; 3 Curtin Institute for Computation, Curtin University, GPO Box U1987, Perth, WA 6845 Australia

**Keywords:** Fungal effector proteins, Template-based modelling, 3D structure, Structural families, Cytotoxic peptides

## Abstract

The discovery of new fungal effector proteins is necessary to enable the screening of cultivars for disease resistance. Sequence-based bioinformatics methods have been used for this purpose, but only a limited number of functional effector proteins have been successfully predicted and subsequently validated experimentally. A significant obstacle is that many fungal effector proteins discovered so far lack sequence similarity or conserved sequence motifs. The availability of experimentally determined three-dimensional (3D) structures of a number of effector proteins has recently highlighted structural similarities amongst groups of sequence-dissimilar fungal effectors, enabling the search for similar structural folds amongst effector sequence candidates. We have applied template-based modelling to predict the 3D structures of candidate effector sequences obtained from bioinformatics predictions and the PHI-BASE database. Structural matches were found not only with ToxA- and MAX-like effector candidates but also with non-fungal effector-like proteins—including plant defensins and animal venoms—suggesting the broad conservation of ancestral structural folds amongst cytotoxic peptides from a diverse range of distant species. Accurate modelling of fungal effectors were achieved using RaptorX. The utility of predicted structures of effector proteins lies in the prediction of their interactions with plant receptors through molecular docking, which will improve the understanding of effector–plant interactions.

**Supplementary Information:**

The online version contains supplementary material available at 10.1007/s12033-023-00703-4.

## Introduction

Effector proteins are secreted from the cells of plant pathogenic fungi and typically locate to the plant cytoplasm (cytoplasmic effectors) or apoplastic space (apoplastic effectors), to cause disease in plants [1]. Secreted effector proteins are usually less than 200 amino acids long and cysteine-rich, particularly in the case of apoplastic effectors. Studies of effector proteins have been ongoing for over three decades [2], with the identification of new effectors crucially allowing the screening of disease resistance/susceptibility in the breeding of new plant cultivars. However, their identification has not been straightforward because most have low sequence similarity with other known fungal effectors or other proteins [3, 4]. Sequence similarity is low amongst many fungal effectors due to high genome plasticity, which plays an important role in fungal pathogenic fitness [5–7]. This is attributed to high rates of mutation in structurally plastic and repeat-rich genome regions that typically containing effector proteins [6, 7]. However, a few effector families can be identified based on loosely conserved sequence motifs or domains, such as the RxLR-like and LysM families [8]. Recently developed tools have enabled computational prediction of effector candidates based on predicted sequence properties with limited success, such as EffectorP 3.0 [9] and Predector [10], and remote homology has also been used to predict structural homology from sequence data with RemEff [3]. However, these predictive methods do not offer the level of functionality that could be achieved using homologous three-dimensional (3D) protein structures.

In 2015, de Guillen et al. reported the presence of structural similarities amongst *Magnoparthe oryzae* and ToxB (*Pyrenophora tritici repentis*) effectors, termed the MAX family, despite having less than 20% sequence similarity [11]. Similar observations have also been reported in other effector families with available 3D protein structures resolved by X-ray crystallography (XRD) or nuclear magnetic resonance (NMR), such as ToxA family, as well as for a number of lone members: RNAse like protein expressed in the haustoria (RALPH), knottin-like and Tox3 [12–14]. None of these effectors have sequence similarity to any other fungal effectors or known proteins despite having similar structures/secondary structures [11]. This demonstrates the challenge in sequence-based identification of these effector families, but holds out hope for structure-based identification.

The increased rate of genome sequencing of agriculturally important plant pathogenic fungi has led to a substantial increase in genome and associated ‘-omics’ data to enable the search for new fungal effectors; however, the aforementioned challenges to sequence-based identification have imposed significant bottlenecks [4, 15]. There is currently an abundance of unconfirmed effector candidate sequences lacking 3D structures [3, 4, 15, 16], but solving their 3D structures using XRD and NMR approaches, although highly accurate, requires substantial resources and time. The application of computational approaches, such as template- and non-template-based modelling, can enable the determination of the structure of fungal effector candidates from their sequences, which might help to address this problem. In template-based modelling, structure-based identification of effectors relies on available 3D structures as a template/reference during modelling, which involves the use of homology modelling, threading and/or fold recognition approaches. Template-based modelling may thus enable the categorisation of many effector candidates into either established or novel structural families on the basis of a common predicted structural fold/motif and/or overall structural homology. This may also provide additional support for the experimental validation of effector proteins that are predicted to belong to structural effector families based on sequence-derived predictions. In addition, template-based modelling may enable the identification of novel folds/motifs if a predicted structure contains at least some regions that are homologous to a known, non-effector protein structure. There is a limited number of currently known structural effector families, and many more folds/motifs are yet to be discovered, which is indicated by broad survey of proteins folds found in nature [17].

**Fig. 1:**
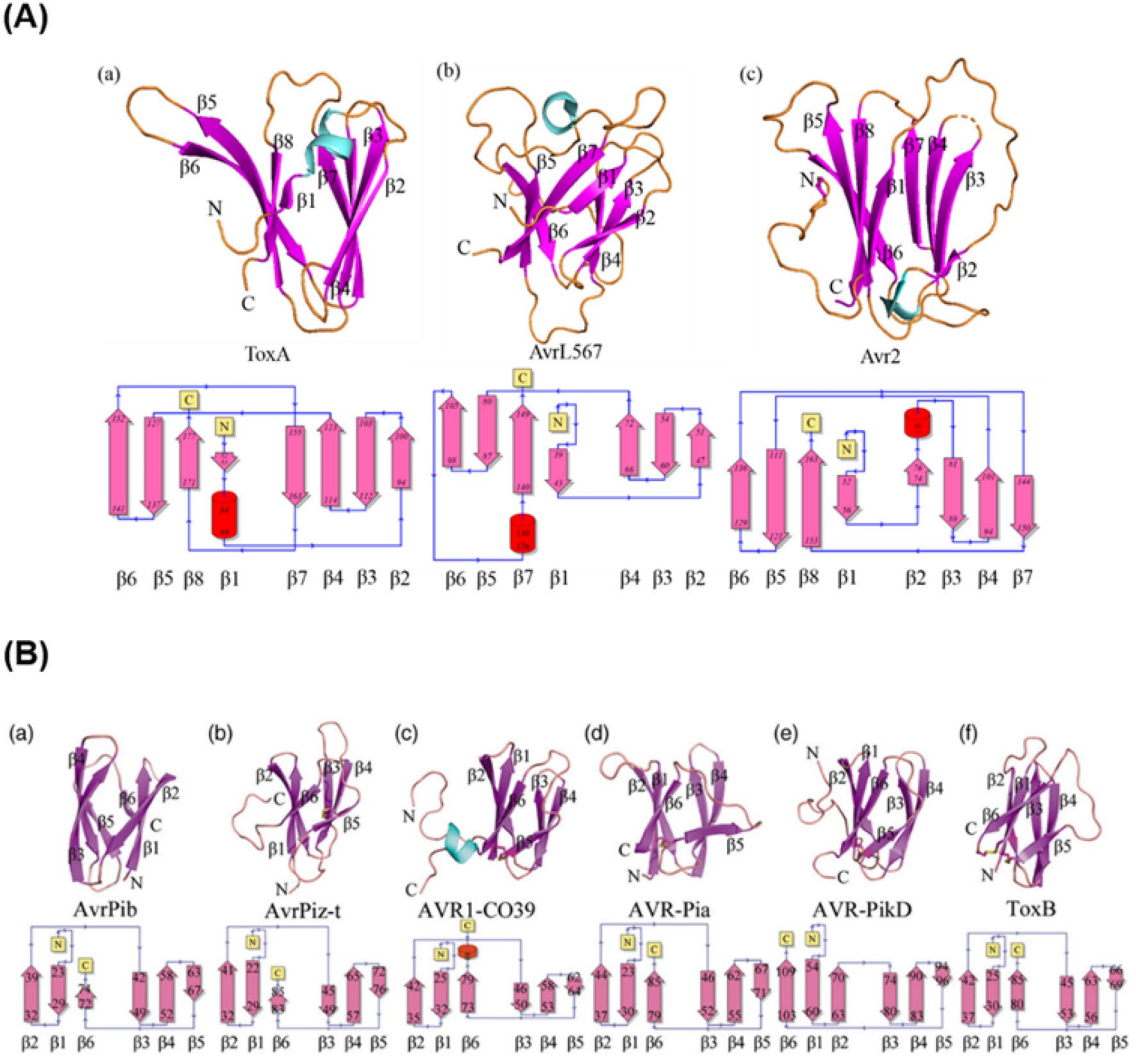
Structures of effector proteins. **A** ToxA-like family with ToxA (a), AvrL567 (b) and Avr2 (c), and **B** MAX-like family with AvrPib (a), AvrPizt (b), Avr1CO39 (c), AvrPia (d), AvrPikD (e) and ToxB (f). Structures are shown in ribbon representation with β-strands coloured in purple, α-helices in cyan and loops in gold. The structural topology of the secondary structure is shown below each protein. Panel **B** was adapted from Zhang et al. (2018). Copyright (2018) John Wiley and Sons

This study focuses on the well-established ToxA and MAX effector families, which contain virulence factors of devastating cereal diseases, caused by fungal pathogen species including *M. oryzae* (infecting rice), *P. tritici repentis* (wheat), *Parastagonospora nodorum* (wheat) and *Bipolaris sorokiniana* (maize). Both families have the largest number of experimentally confirmed 3D protein structures amongst the currently proposed structural families of fungal effectors. The ToxA-like family structures consist of ToxA, Avr2 and AvrL567 whilst the MAX family structures consist of ToxB, AvrPia, AvrPib, AvrPizt and AvrPik variants. Both families share a β-sandwich fold, with an additional short α-helix at the N-terminus found in the ToxA-like effectors (Fig. 1).

To date, there is no structure-based approach in the context of template-based modelling dedicated to the prediction of fungal effector protein structures, largely due to the limited number of experimentally confirmed structures available. Secondly, template-based modelling is dependent on sequence homology between template and the sequence candidates in order to make accurate predictions, which is challenging because sequence homology is typically low amongst fungal effector proteins. Until recently, it was also not computationally feasible to model thousands of effector candidate structures in a high-throughput manner to enable large-scale structural homology comparisons. Very recently, a significant number of public protein datasets for multiple species has become publicly available via the DeepMind (AlphaFold) project [18], which uses ab initio or non-template-based approaches for protein modelling.

Template-based modelling can generally be categorised into basic homology modelling, threading and fold recognition, and the selection of each approach depends on the percentage of sequence similarity of the target sequence with its template(s). In general, the most commonly used homology modelling programs are SWISS-MODEL and MODELLER [19]. Other modelling programs such as Phyre2 [20], I-TASSER [21] and RaptorX [22, 23] are based on threading and fold recognition. These programs have been widely used to successfully predict the structure of a diverse range of proteins from different organisms. The main advantage of using template-based modelling compared to non-template-based methods is the higher confidence and reliability of the predicted structures, since an existing experimentally confirmed structure (template) guides the folding, rather than following a first principles (ab initio) approach. Studies of template-based modelling of uncharacterised sequence candidates have also shown it to be reliable for “transferring” protein function of homologous proteins onto the predicted structures [24].

The application of computational modelling to fungal effector proteins has been recently published by Seong et al. based on the secretome of *M. oryzae* [25]. Structures were predicted for effector protein candidates obtained from secreted and non-transmembrane sequence sets of *M. oryzae* secretome. Protein modelling was done using TrRosetta, which applies co-evolutionary information from multiple sequence alignment (MSA) of the target sequences and homologues, and the template library for MSA was obtained from the I-TASSER server. Full atomic models were generated using PyRosetta and the structures with the lowest energy score were selected. Predicted structures were assessed with RUPEE, SCOP and CATH. The study reported here differs from this previous work because it focusses on elucidating the structure of fungal effector proteins from diverse fungal species and improving overall knowledge of effector structural families, rather than a single species. Furthermore, in this study, a fully structure-based rather than sequence-based approach was adopted. Template-based modelling was assessed for its ability to model the structure of fungal effector proteins, and the advantages and limitations of this approach are explored.

## Methods

### Effector Candidate Sequences

Thirty-three ToxA candidate protein sequences were obtained from bioinformatics predictions generated using sequence-derived hidden-Markov model (HMM) clustering (remote homology) [3] (Fig. S1; Table S1 in Supporting Information, SI), and 55 MAX effector candidate protein sequences were obtained from de Guillen et al*.* [11] (Fig. S2; Table S2 in SI). Protein sequences of 87 effector candidates (with experimentally validated virulence phenotypes) were downloaded from PHI-base v4.4 [26] and based on the training dataset used in EffectorP version 1.0 and 2.0 [27, 28] (Fig. S3 in SI). The training dataset includes some effectors with structures available in the PDB and which were subsequently omitted from effector candidate analysis in this study but were used for benchmarking. All sequences were obtained in FASTA format and were pre-processed with SignalP v4.1 [29] using default parameters to remove the N-terminal secretion signal peptide, resulting in mature secreted sequences. Sequences of fungal effector candidates within the same family were viewed and aligned using BioEdit [30] under ClustalW alignment with default parameters [31]. Figures S1 and S2 in SI show the sequence alignment of the candidates of ToxA and MAX-like effector families, respectively. Details of effector candidate sequences are compiled in Tables S1 and S2 in SI.

### Template-Based Modelling

RaptorX version 3.0 (http://raptorx.uchicago.edu/) [23] was used to model the structure of effector candidates. Both the web and standalone versions were utilised. RaptorX ranks models based on the top RaptorX score, and each model is assigned a *p*-value, RaptorX score, un-normalised global distance test (uGDT)/GDT, un-normalised sequence identity (uSeqID)/SeqID, alpha carbon (Cα) root mean square displacement (RMSD) and TM-score. RaptorX score is the alignment score in a range between 0 and the total sequence length, with 0 being the worst. SeqID/SeqID is the number of identical residues in the alignment. SeqID is uSeqID normalised by the sequence length and multiplied by 100. Higher uSeqID/SeqID is better and SeqID of > 30% (in proteins of > 200 amino acid residues) suggests that the predicted model has a correct fold [22, 32]. uGDT is un-normalised GDT, defined as 1 × *N*(1) + 0.75 × *N*(2) + 0.5 × *N*(4) + 0.25 × *N*(8), with *N*(*x*) being the number of residues with estimated modelling error in Å smaller than *x*. GDT is uGDT normalised by the protein domain length, and measures the quality of a model by comparing it with the native structure, having a value of 0 to 100. For a protein with > 100 amino acid residues, uGDT > 50 is an indicator of good quality. For a protein < 100 amino acid residues, GDT > 50 is an indicator of good quality. *p*-value assesses the relative quality of a model, with smaller *p*-values reflecting better model quality. A small *p*-value < 10^−5^ also indicates that the model has uGDT/GDT ≥ 50 [23].

### Comparison with Other Template-Based Modelling Approaches

Two other well-known template-based modelling programs were used for comparison with RaptorX: SWISS-MODEL (homology modelling) (https://swissmodel.expasy.org/) [19] and Phyre2 (fold recognition) (http://www.sbg.bio.ic.ac.uk/phyre2/) [20]. Both of these programs were used through their online webservers. In SWISS-MODEL, the automated mode was applied, whereby suitable templates are identified based on BLAST and HHblits searches. In the case of Phyre2, its normal mode was applied, which uses HHblits and fold library scanning.

### Structural Analysis

The predicted protein structures were superimposed onto their respective templates based on their Cα, and the RMSD and TM-score values were calculated using TM-align [33]. TM-score compares structures based on global topology and is less sensitive to local structure variations compared to the use of RMSD. TM-score values range between 0 and 1, where a score greater than 0.5 indicates that structures have the same fold, and a higher value indicates greater similarity [34]. Domain and fold analysis of the predicted models was done using CATH [35, 36] and SCOP [17, 37]. The structural quality of the predicted models was assessed using PROCHECK [38]. Protein structures were visualised using PyMol Open Source (https://github.com/schrodinger/pymol-open-source).

## Results

In this study, three different candidate sequence datasets were used as input for template-based modelling: ToxA-like effectors, MAX-like effectors, and phenotypically validated virulence proteins obtained from PHI-base. The first two datasets were obtained from bioinformatics sequence-based predictions implementing sequence–profile/profile–profile-based approaches specific for ToxA and MAX effector families [3, 11]. The identification of ToxA-like candidates applied a remote homology clustering method using MMSeq2 profiles, HMM–HMM searches and HHBlits search iterations [3], whilst sequences of MAX-like candidates were obtained using HMM search, NCBI PSI-BLAST and HMMERsearch (HMMER v 3.0) [11]. The templates used in the modelling of these two datasets were thus expected to correspond to either ToxA or MAX structures. The third sequence dataset from PHI-base contains some effector sequences with structures available in the PDB, and which were excluded as effector candidates and instead later used instead as input for the benchmarking of template-based modelling approach ("Modelling of Fungal Effector Candidates Using Templates from Known Effector Structural Families" section). This third dataset was also tested for predicted 3D models that matched known structural families.

We divide the presentation of our findings into three major parts. "Modelling of Fungal Effector Candidates Using Templates from Known Effector Structural Families" section presents the predictions of template-based modelling with matches to templates that belong to known effector structural families, which includes sequence datasets of ToxA-like and MAX candidates as well as phenotypically validated PHI-base sequences. "Modelling of Fungal Effector Candidates Based on Templates Belonging to Non-effector Proteins" section presents and discusses the predictions of structural modelling that used templates from protein structures other than the known fungal effector structural families. "Comparison with Other Template-Based Modelling Programs" section presents the comparison of effectors modelled with RaptorX with SWISS-MODEL and Phyre2.

### Modelling of Fungal Effector Candidates Using Templates from Known Effector Structural Families

This subsection describes the predictions of the modelling of the structure of effector candidates from ToxA-like, MAX-like and phenotypically validated virulence proteins that utilises templates from known fungal effector structural families using RaptorX. Currently, there are thirteen known effector structural families, with ToxA and MAX having the largest number of resolved 3D structures deposited in the PDB compared to other fungal effector families.

For each candidate sequence, the top five models ranked according to the best RaptorX score are reported, with the top-ranked model deemed to be the best model. In addition to the scoring function given by RaptorX and its corresponding *p*-value, RaptorX score, uGDT/GDT, uSeqID/SeqID, Cα–RMSD and TM-score were used to determine structural similarity between the modelled structure (target) and the reference template structure. The sequences of fungal effector proteins with available structures in the PDB (Table 1; Fig. S4 in the SI) were used for benchmarking to determine the optimum cut-offs for the scoring functions used in RaptorX, which were subsequently applied for the assessment of all effector candidates.

**Table 1:** Benchmarking predictions using RaptorX for sequences of effectors with 3D structures available in the PDB

Structural family	Effector (PDB ID)	*p*-value	RaptorX score	uGDT/GDT	uSeqID/SeqID	RMSD (Å)	TM-score
ToxA	AvrL567A (2opcA)	1.60E−13	100	119/93	115/91	0.12	0.99908
	ToxA (1zldA)	3.20E−06	70	96/59	97/60	0.13	0.98892
	Six3 (5od4A)	2.50E−09	96	120/84	123/85	0.12	0.99916
MAX	ToxB (2mm0A)	3.10E−06	60	64/100	64/100	0.16	0.99655
	Avr1Co39 (2myvA)	9.40E−10	60	67/100	67/100	0.19	0.84511
	AvrPia (5jhjA)	2.70E−06	62	65/99	66/100	0.16	0.83321
	AvrPik (5a6wC)	1.60E−12	76	84/91	83/90	0.13	0.99843
	AvrPizt (2lw6A)	1.20E−10	66	77/86	80/89	0.14	0.99805
Zinc-binding	AvrP123 (5vjjA)	7.80E−13	65	65/69	48/51	0.89	0.94371
LARS	AvrLm4-7 (4fprA)	2.80E−10	98	107/87	118/97	0.37	0.93397
RxLR	AvrM (4bjmA)	1.70E−06	243	204/71	226/79	0.39	0.99636
C2-like	PevD1 (5xmzA)	2.40E−09	125	110/81	122/89	0.14	0.99891
Regulatory enzyme	Cmu1 (6fpgC)	1.19E−08	234	235/87	262/97	0.07	0.99987
Hydrolase/lipase	FGL1 (3ngmA)	3.10E−12	325	275/82	293/87	0.65	0.99313
Chitin-binding	Ecp6 (4b8vA)	9.50E−12	158	160/76	189/90	0.10	0.99965
	Avr4 (6bn0A)	1.60E−11	64	66/56	79/68	0.17	0.99718
Necrosis inducing	NEP1 (3gnuP)	6.85E−19	204	174/77	89/39	0.26	0.98383
	NIP1 (1kg1A)	2.63E−09	53	55/88	60/97	0.242	0.99162

The table reports the *p*-value, RaptorX score, uGDT/GDT, uSeqID/SeqID, PDB ID of the template, Cα–RMSD and TM-score of the predicted models

In the benchmarking predictions, RaptorX scores for all predicted effector models were higher than 50 (Table 1), which confirmed the reliability of this scoring function since a model with a score above 50 is highly likely to exhibit the correct fold of the target sequence [22]. The highest RaptorX score was 325 for effector FGL1 and the lowest was 53 for NIP2. TM-score values were all above 0.8, ranging from the highest value of 0.99987 to the lowest value of 0.83321, revealing the high similarity of the predicted models to the respective 3D reference structures.

#### ToxA-Like Effector Candidates

The prediction of the structures of all 33 ToxA-like effector candidates used the structure of ToxA effector (PDB structure 1zldA) as the template in the modelling of the top-ranked model (Table 2). The lowest RaptorX score was 41 and the highest was 63. Twenty-one of the predicted models had a RaptorX score of 50 and above, which was regarded as successful. TM-score values ranged from the lowest of 0.66836 to the highest of 0.93961. The remaining twelve models had a RaptorX score below 50 (41–49) and three of these models had the lowest RaptorX score of 41 (p0de_mRNA10272, p2g1_PZD05769.1 and p2g2_PZC93680.1), which were predicted to have two β-strands missing out of the seven β-strands forming the β-sandwich structure of ToxA effector (Fig. S5 in SI) due to the absence of 28 residues in the C-terminal region. This deficiency was, however, not observed in the models with a RaptorX score of 50 and above since the length of the target sequences were either equal or larger than the length of the template sequence. The sequence length and missing β-strands likely contributed to the low RaptorX scores of the predicted models.

**Table 2:** RaptorX predictions for the top-ranked models of each ToxA-like effector candidate that had a match to a template in the ToxA-like structural family, sorted by alphabetical order

ToxA-like effector candidates	*p*-value	RaptorX score	uGDT/GDT	uSeqID/SeqID	Template PDB ID	RMSD (Å)	TM-score
p05c_mRNA16607	1.9 × 10^−4^	49	71/53	18/13	1zldA	1.12	0.88709
p05d_mRNA9122	1.5 × 10^−4^	49	68/51	17/13	1zldA	0.92	0.89386
p05e_mRNA13670	2.3 × 10^−4^	49	72/53	19/14	1zldA	1.00	0.88914
p05g_mRNA17320	1.9 × 10^−4^	50	71/53	19/14	1zldA	0.87	0.90005
p05k_mRNA3392	1.9 × 10^−4^	50	71/53	19/14	1zldA	0.87	0.90005
p05m_mRNA12409	1.9 × 10^−4^	50	71/53	19/14	1zldA	0.91	0.90067
p05n_mRNA11205	1.9 × 10^−4^	50	71/53	19/14	1zldA	0.99	0.89235
p09v_mRNA10419	1.9 × 10^−4^	51	69/49	18/13	1zldA	1.26	0.87117
p09v_mRNA9195	7.3 × 10^−4^	47	64/46	17/12	1zldA	1.10	0.8552
p0dd_mRNA2255	7.0 × 10^−5^	51	71/53	23/17	1zldA	1.01	0.89151
p0de_mRNA10272	5.4 × 10^−4^	41	62/61	18/18	1zldA	0.84	0.66836
p1ap_mRNA3793	2.2 × 10^−4^	49	72/55	19/15	1zldA	0.87	0.90312
p1b1_EXF72942.1	2.3 × 10^−4^	49	72/53	19/14	1zldA	0.91	0.89719
p1bd_mRNA10016	2.0 × 10^−4^	49	71/53	19/14	1zldA	0.79	0.90687
p1bd_mRNA1147	1.8 × 10^−4^	50	70/53	18/14	1zldA	1.07	0.88942
p1bi_OBR06575.1	1.6 × 10^−4^	48	66/49	15/11	1zldA	1.32	0.89174
p1bo_mRNA4951	2.0 × 10^−4^	49	71/54	18/14	1zldA	0.82	0.90455
p22r_EXK24251.1	8.9 × 10^−5^	51	66/49	20/15	1zldA	1.16	0.88646
p2fk_EMD96331.1	4.6 × 10^−5^	63	96/66	38/26	1zldA	0.39	0.9509
p2fl_ENH98532 .1	4.6 × 10^−5^	63	96/66	38/26	1zldA	0.39	0.95141
p2fn_EUC44184.1	9.1 × 10^−5^	56	67/55	27/22	1zldA	1.19	0.90785
p2fq_EUC36307.1	2.3 × 10^−4^	56	71/57	18/14	1zldA	0.73	0.93061
p2g0_EFQ93895.1	7.0 × 10^−5^	51	71/53	23/17	1zldA	1.01	0.89152
p2g1_PZD05769.1	5.4 × 10^−4^	41	62/61	18/18	1zldA	0.79	0.67131
p2g2_PZC93680.1	5.4 × 10^−4^	41	62/61	18/18	1zldA	0.78	0.67124
p2g3_PZD24241.1	1.0 × 10^−4^	51	74/56	24/18	1zldA	0.85	0.90474
p2g4_PZD32416.1	1.0 × 10^−4^	51	74/56	24/18	1zldA	0.85	0.90474
p2g5_PZD46046.1	1.0 × 10^−4^	51	74/56	24/18	1zldA	0.92	0.89835
p2g6_PWO08528.1	1.0 × 10^−4^	51	74/56	24/18	1zldA	0.85	0.90474
p2g7_PZD04407.1	1.0 × 10^−4^	51	74/56	24/18	1zldA	0.94	0.89685
p2g8_PWO20795.1	1.0 × 10^−4^	51	74/56	24/18	1zldA	0.92	0.89835
p2g9_EDU49735.1	1.0 × 10^−4^	51	74/56	24/18	1zldA	0.85	0.90474
p2gb_RAQ98980.1	1.6 × 10^−4^	52	71/53	21/16	1zldA	0.89	0.91064

The table reports the *p-*value, RaptorX score, uGDT/GDT, and uSeqID/SeqID, as well as the structure used as the reference template (PDB ID). Pairwise structural differences between each model and reference template are estimated by Cα–RMSD and TM-score

**Fig. 2:**
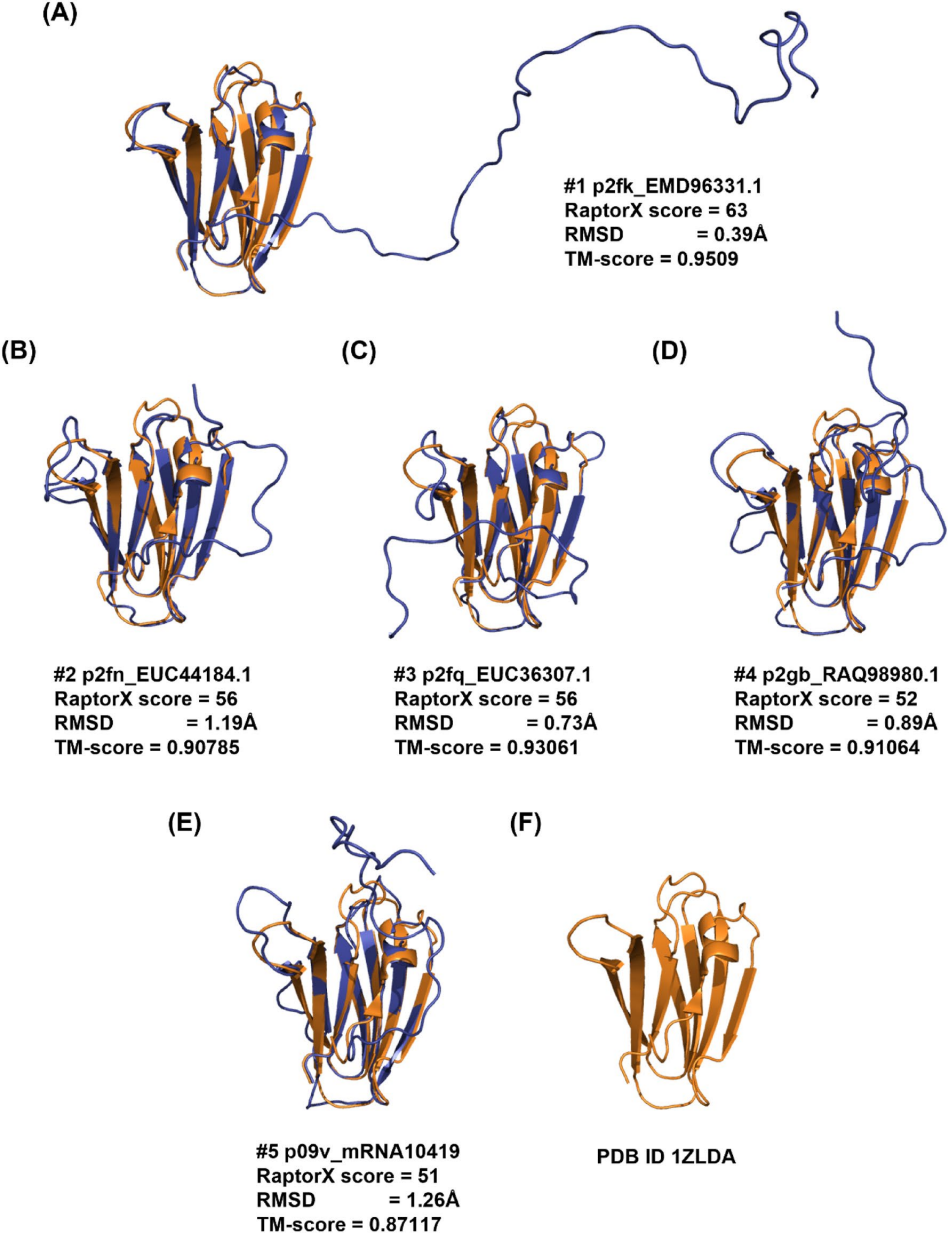
Top five models predicted by RaptorX for different ToxA-like effector candidates sorted according to decreasing RaptorX score (**A**–**E**). Each model (shown in blue) was superimposed onto its corresponding ToxA reference structure (PDB ID 1ZLD) (**F**), shown in gold. The corresponding Cα–RMSD and TM-score values are reported. The remaining ToxA-like effector candidate models are reported in Fig. S5 in SI

The TM-score of all ToxA-like effector candidates was above 0.5, with candidate p2fq_EUC36307.1 having the best TM-score of 0.93061 (Fig. 2) and p2g2_PZC93680.1 having the lowest TM-score of 0.67124, revealing the high accuracy of the predictions made by RaptorX for these effector candidates.

Figure 2 reveals that the ToxA-like effector candidate p2fk_EMD96331.1 had the highest RaptorX score of 63, and its superimposition with the ToxA reference template structure (PDB structure 1zld) resulted in a Cα–RMSD of 0.39 Å and TM-score of 0.9509, indicating its high structural similarity to the ToxA structure. The superimposition of the effector candidates with the template structure show that all β-strands overlap well, including the short α-helix at the N-terminus. An N-terminal tail region, 17 to 47 amino acid residues long, was observed as an extended sequence region that does not overlap with the template structure, which lacks this extension; however, it should be noted that the sequence of the template structure (PDB structure 1zld) has an N-terminal tail 19 residues long that was not visible in the electron density due to the conformational flexibility of this region. This region forms a long-disordered loop and does not have any match to any domain in the public databases. Similar N-terminal extensions with different lengths were observed in all candidate models (Fig. S5 in SI).

#### MAX-Like Effector Candidates

RaptorX generated five models for each MAX-like effector candidate, each using a different reference template structure (Table S3 in SI). These templates were selected and ranked based on their RaptorX scores, as explained in the “Methods” section. A total of 19 out of 55 candidates had a match to a member of the MAX structural family (Table 3). As for the remaining 36 candidates, six of them had at least one MAX template structure listed amongst the top five templates used in the modelling (highlighted in bold in Table S3 in SI). Since they were not ranked as the top template, the RaptorX scores of those templates were below 50, with 25 being the highest and 17 the lowest, indicating that they were not the best template to be applied in the modelling of MAX effector candidate structures.

**Table 3:** RaptorX predictions for the top-ranked models of each MAX-like effector candidate that had a match to a template in the MAX structural family, sorted by alphabetical order

MAX-like effector candidates	*p*-value	RaptorX score	uGDT/GDT	uSeqID/SeqID	Template PDB ID	RMSD (Å)	TM-score
M.BR29.EuGene_00081821	1.20E−02	25	40/36	9/8	2mm2A	0.72	0.92004
M.BR29.EuGene_00085071	7.20E−04	28	41/71	20/34	2mm2A	0.9	0.81525
M.BR29.EuGene_00088411	2.30E−03	26	37/49	10/13	2mm2A	0.47	0.97549
**M.BR29.EuGene_00106461**	**3.70E−05**	**56**	**58/90**	**29/45**	**2mywA**	**0.31**	**0.80389**
M.BR29.EuGene_00107481	1.60E−02	16	24/30	8/10	5a6wC	0.87	0.68531
M.BR29.EuGene_00119491	1.00E−02	21	34/44	19/25	2myvA	1.63	0.73718
M.BR29.EuGene_00119511	4.30E−04	28	41/51	18/23	2myvA	1.13	0.74793
M.BR29.EuGene_00121691	8.70E−03	23	30/49	11/18	5zngC	0.56	0.93489
M.BR29.EuGene_00125811	1.20E−02	17	26/26	12/12	2myvA	0.5	0.82891
**M.BR29.EuGene_00126081**	**5.90E−06**	**54**	**50/74**	**21/31**	**2mm2A**	**0.37**	**0.95371**
**M.TH16.EuGene_00040131**	**5.00E−05**	**54**	**53/79**	**29/43**	**2mywA**	**0.24**	**0.81836**
M.TH16.EuGene_00045871	5.30E−03	22	27/44	7/11	5zngC	0.47	0.95616
M.TH16.EuGene_00099371	5.00E−03	21	24/35	8/12	2myvA	2.5	0.5092
M.TH16.EuGene_00106621	2.90E−03	26	47/74	19/30	2mm2A	0.73	0.85801
M.TH16.EuGene_00124981	1.60E−03	18	25/35	14/19	5a6wC	0.63	0.73633
M.TH16.EuGene_00127871	6.00E−03	18	34/38	20/23	2myvA	0.8	0.7976
M.TH16.EuGene_00134971	5.20E−03	21	25/42	11/18	5zngC	0.63	0.91542
**M.TH16.EuGene_00135161**	**9.10E−06**	**53**	**50/74**	**20/29**	**2mm2A**	**0.36**	**0.95411**
MGG_17132	1.00E−02	22	38/57	12/18	2mm2A	0.9	0.86823

The table reports the *p-*value, RaptorX score, uGDT/GDT, and uSeqID/SeqID, as well as the structure used as the reference template (PDB ID). Pairwise structural differences between each model and reference template are estimated by Cα–RMSD and TM-score. Effector candidates with RaptorX scores above 50 are shown in bold

**Fig. 3:**
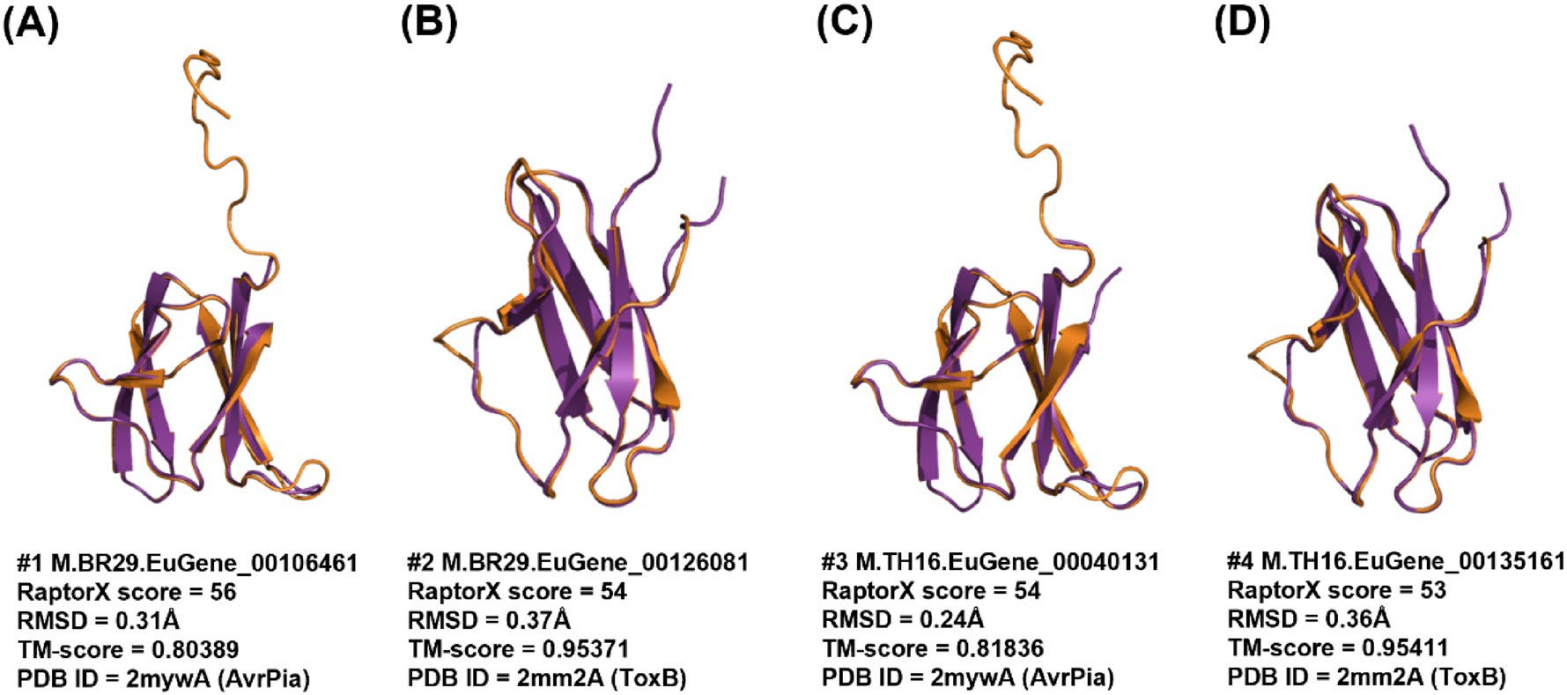
Top models predicted by RaptorX for MAX-like effector candidates using structures of the MAX structural family as template, sorted according to decreasing RaptorX score (**A**–**D**). The predicted models (shown in purple) were superimposed onto their corresponding MAX family reference template structure (shown in gold). The corresponding RaptorX score, Cα–RMSD, TM-score and template PDB ID are reported

The predicted models for the effector candidates each used a structure from the MAX structural family as the best template (PDB ID shown in brackets): ToxB (2mm2A), AvrPia (2mywA), AvrPikD (5a6wC), and Avr1C039 (2myvA and 5zngC). The highest RaptorX score for a MAX-like candidate model was 56, with 16 being the lowest. A total of four models had a RaptorX score above 50 (shown in bold in Table 3): M.BR29.EuGene_00106461, M.BR29.EuGene_00126081, M.TH16.EuGene_00040131 and M.TH16.EuGene_00135161 (Fig. 3). The Cα–RMSD of these models ranged from 0.24 to 0.37 Å, whilst TM-scores ranged from 0.80389 to 0.95411, both indicating a high degree of similarity between the models and their MAX effector reference templates.

The remaining 15 models that used MAX effectors as templates had a RaptorX score below 50 (in the range of 16–28). Model M.BR29.EuGene_00107481 with the lowest RaptorX score of 16 was predicted to have all six β-strands, resembling MAX effector (Fig. S6 in SI). There were no missing strands in this model such as is the case of the ToxA-like effector candidate with the lowest RaptorX score of 41. Nonetheless, the model of M.BR29.EuGene_00107481 does appear to reflect the structure of MAX template, which is supported by a relatively lower TM-score of 0.68531. Overall, the TM-score for all nineteen MAX effector candidates that used MAX structures as templates were above 0.5, with candidate M.BR29.EuGene_00088411 having the best TM-score of 0.97549 and M.TH16.EuGene_00099371 having the lowest TM-score of 0.5092.

Figure 3 reveals that the model for the MAX-like effector candidate M.BR29.EuGene_00106461 had the highest RaptorX score of 56 and its superimposition with MAX structure effector AvrPia (PDB structure 2myw) had a Cα–RMSD of 0.31 Å and TM-score of 0.80389, indicating good structural similarity with the MAX template structure. The superimposition of the model with the template structure shows that all β-strands overlap well. Most of the MAX candidates had an additional loop at the N-terminal region, ranging from 5 to 10 amino acids long, observable in each of the displayed candidate models (Fig. 3). This region does not have any match to any domain in the public database. The remaining 36 predicted models of MAX-like effector candidates can be found in Fig. S6 in SI.

#### Phenotypically Validated Virulence Proteins from PHI-Base

**Fig. 4:**
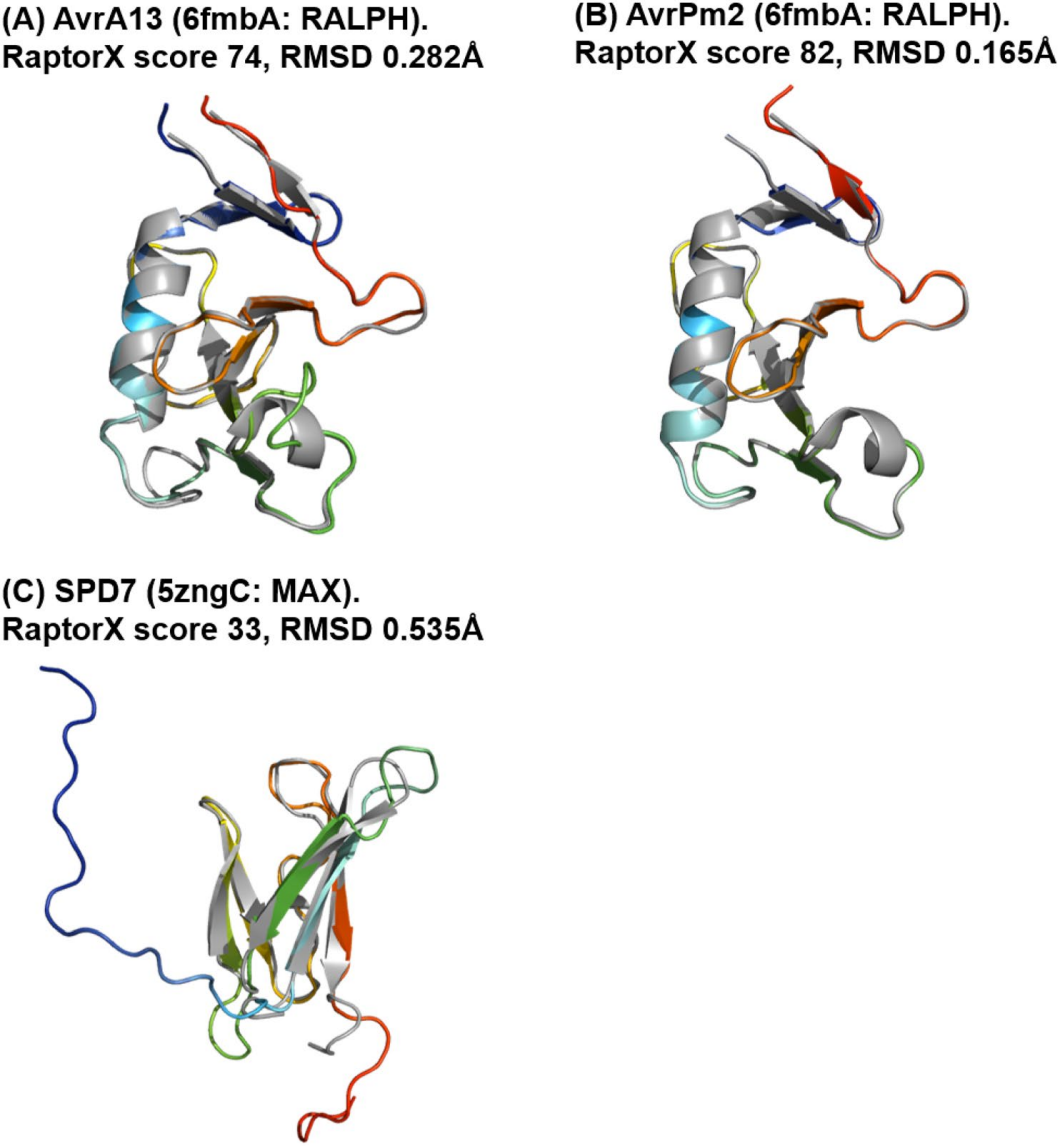
Predicted models (in rainbow) of phenotypically validated fungal effectors derived using a template from a known fungal effector structural family: RALPH (**A** and **B**) (PDB ID 6fmbA) and MAX (**C**) (PDB ID 5zngC). The Cα–RMSD of the superimposition of the RaptorX model with the reference template structure (in grey) is reported.

Only three out of 87 candidates could be modelled using structural templates that belong to two known fungal effector structural families: RALPH (PDB structure 6fmbA) and MAX (PDB structure 5zngC) (Table 4; Fig. 4).

**Table 4:** RaptorX predictions for the top-ranked models of phenotypically validated fungal effector candidates that used a template from known effector structural families, sorted by alphabetical order

Effector name	Template PDB ID	*p*-value	RaptorX score	uGDT/GDT	uSeqID/SeqID	RMSD (Å)	TM-score
AvrA13	6fmbA	1.80E−07	74	64/64	13/13	0.47	0.95356
AvrPm2	6fmbA	4.00E−08	82	69/70	40/41	0.15	0.99823
SPD7	5zngC	3.50E−03	33	21/23	10/11	1.70	0.86893

The table reports the *p-*value, RaptorX score, uGDT/GDT and uSeqID/SeqID, as well as the structure used as the reference template (PDB ID). Pairwise structural differences between each model and reference template are estimated by Cα–RMSD and TM-score

AvrA13 and AvrPm2 were modelled using a template structure from the RALPH family: CSEP0064/BEC1054 (PDB structure 6fmbA), with a Cα–RMSD of 0.282 Å and 0.165 Å, respectively. AvrA13 has six β-strands and an α-helix, whilst AvrPm2 has five β-strands and an α-helix. SPD7 was modelled using a template structure from the MAX effector family (PDB structure 5zngC), with Cα–RMSD of 0.535 Å. SPD7 has a β-sandwich fold similar to MAX, but is missing one β-strand, which might explain why it had a RaptorX score below 50. Both AvrA13 and AvrPm2 are known to belong to the RALPH family based on their sequence alone, with the RALPH effector family being conserved amongst cereal mildews [39], such that the modelling of their structures did not in fact add any advantage for the categorisation of these effector candidates. AvrA13 is secreted by *Blumeria graminis* f. sp. *hordei*, a powdery mildew pathogen of barley. With well-conserved RNAse domains, the sequences of the RALPH family are more conserved compared to the ToxA-like and MAX effector families. AvrPm2 is an RNase-like avirulence effector that is a wheat/rye powdery mildew fungus. There are currently no experimentally determined structures of AvrA13 and AvrPm2, although predicted 3D models have been previously published for both effectors [40, 41]. The model for AvrA13 predicted by Bauer et al. [40] exhibits a similar fold to that observed in our model, except that the lengths of the third and fourth β-strands are shorter in our predicted model, thus generating a longer loop region. Our model is closer to the reference structure BEC1054 except for the missing α-helix between β-strands 3 and 4 (Fig. S7A in SI). In the case of the AvrPm2 models, comparisons were made with predictions reported by Bauer et al*.* and Manser et al. [40, 41]. The third β-strand is missing in the model predicted by Bauer et al*.*, with our model being closer to that predicted by Manser et al., with similar loop regions except that there is an additional 3_10_-helix between β-strands 3 and 4, showing that their predicted structure is closer to the reference BEC1054 (Fig. S7B and Fig. S7C in SI). In the case of SPD7, this is an effector suppressor of plant cell death secreted by *M. oryzae*, for which there are currently no available experimental or predicted 3D structures [42]. Consequently, our model is the first reported predicted structure of SPD7, which had indeed not been previously categorised as belonging to the MAX-like effector protein family.

### Modelling of Fungal Effector Candidates Based on Templates Belonging to Non-effector Proteins

This section describes the prediction of the structure of effector candidates that required the use of a reference template other than structures from known fungal effector families. These template protein structures were found to correspond to non-phytopathogenic fungi, or completely different organisms (plant, animal or even human). This section describes mostly the prediction of phenotypically validated effector candidates, since ToxA-like candidates did not have any matches with template structures from effectors other than from the ToxA structural family.

In the modelling of MAX-like effector candidates, 36 out of 55 effector candidates had a structural match to proteins other than from the MAX structural family. The list of the modelled candidates is reported in Table S3 in SI. These 36 MAX-like effector candidates utilised proteins with various functions as template structure. However, RaptorX scores for these candidates were all below 50, with 31 being the highest for candidate M.BR29.EuGene_00095641 and 16 being the lowest for both candidates M.BR29.EuGene_00091681 and MGG_15207. This is due to the approach used in determining the sequences of MAX-like effector candidates, which is elaborated on in the “Discussion” section. In addition, amongst the list of the top five templates used in the modelling of MAX-like effector candidates, the use of MAX templates was found for six of the predicted models (highlighted in bold in Table S3 in SI). These templates were not the top-ranked template. However, since RaptorX scores were all below 50, the modelled structures are less reliable, which would require validation or the use of an alternative approach for non-template-based modelling.

A total of 61 out of the 66 phenotypically validated effector candidates were modelled with protein templates other than from structures belonging to known fungal effector families. Overall, the best RaptorX score for these effector candidates was 337 and the lowest was 3. Out of the 61 predicted models, 20 have a RaptorX score above 50, whilst the remaining models have a RaptorX score below 50 (Table 5).

**Table 5:**
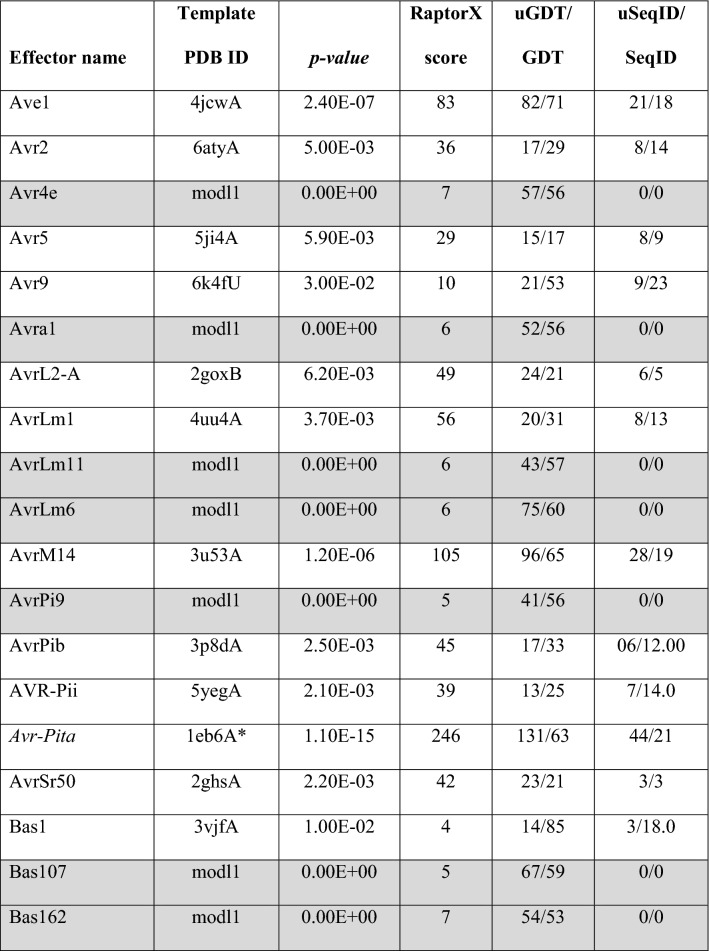

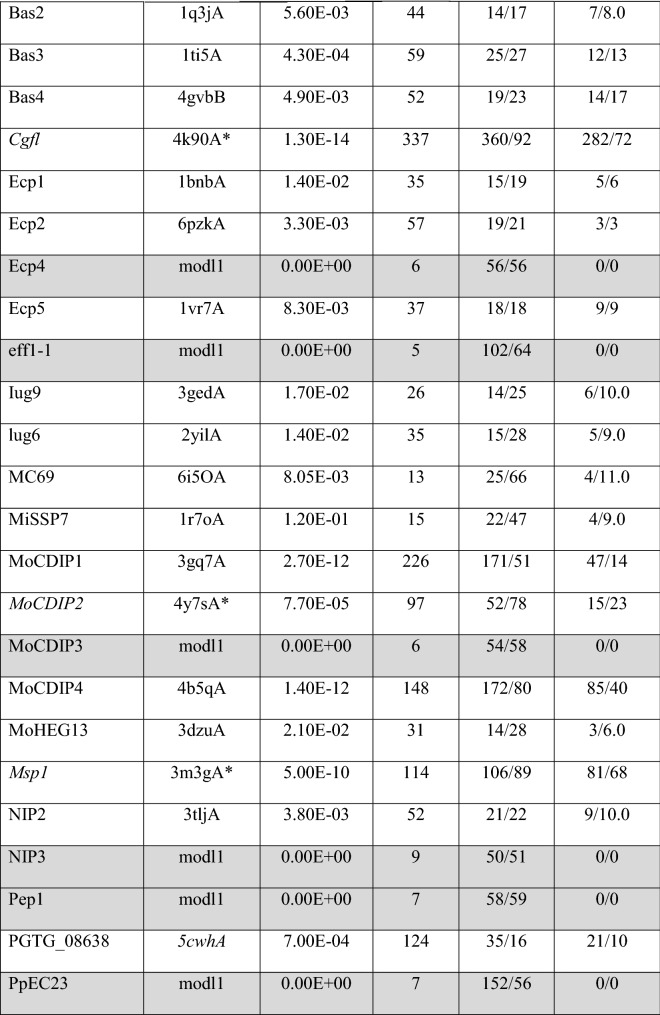

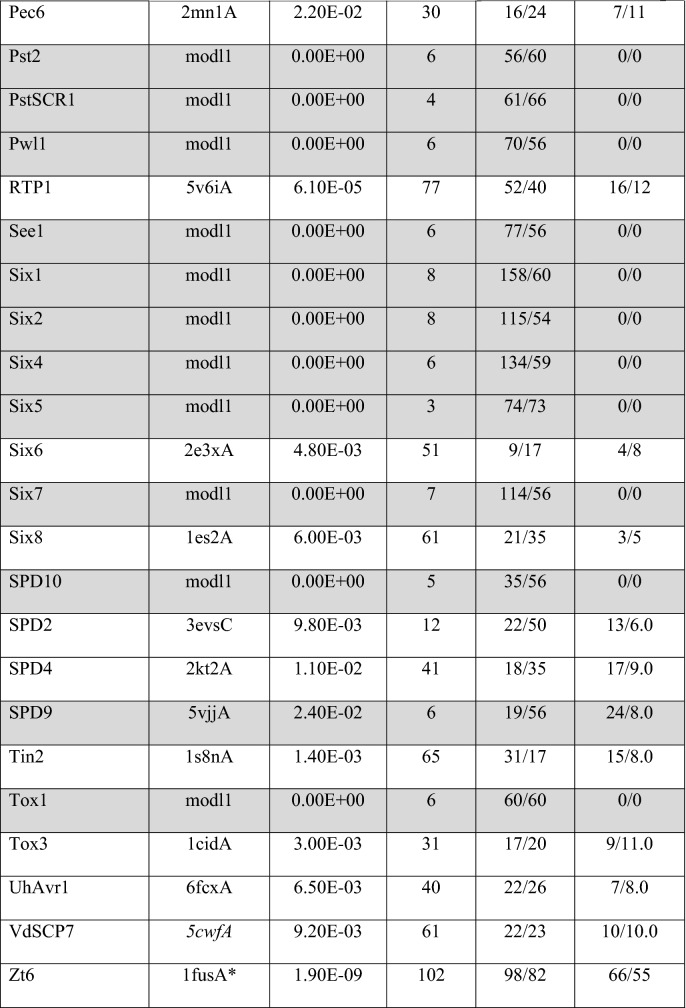
RaptorX predictions for phenotypically validated effector candidates, sorted by alphabetical order

The table reports the *p-*value, RaptorX score, uGDT/GDT, uSeqID/SeqID and the reference structure used as template (PDB ID). The reference name with asterisk (*) indicates the use of a single (high-confidence) template during modelling. Effector names in italics are categorised as conserved sequences. Reference names in italics were modelled using a synthetic construct as reference template structures. Effectors with rows highlighted in grey have no match to any template structure in the public database

The results for this section have been categorised based on the template structures used in template-based modelling: (a) single template, (b) conserved sequences, (c) synthetic constructs, (d) match with any protein in the PDB and (e) no match to any templates (i.e. candidates for non-template-based modelling).

#### Single Template

When a single template is used, the modelling of effector candidates utilises only one template structure during threading instead of a multi-template modelling approach or multi-template threading (MTT), which is used by default in RaptorX. In MTT, RaptorX applies five templates during the threading step for the modelling of protein structures. In this case, five effector candidates were modelled using a single template: Avr-Pita, Cgfl1, MoCDIP2, Msp1 and Zt6, with the corresponding templates being PDB structures 1eb6A, 4k90A, 4y7sA, 3m3gA and 1fusA, respectively (highlighted by an asterisk in Table 5). Theoretically, the use of a single template will result in a high-confidence template compared to having five top templates to assist the modelling. This depends on the scores obtained after template alignment and the scoring step in RaptorX, and it also indicates that template selection prior to modelling has converged (see the Discussion). This is followed by the use of these templates during structure modelling [23]. The RaptorX scores for these effector candidates are all above 50, with the highest RaptorX score being 337, and the lowest being 97. Due to the increase in the accuracy of template selection, the resulting models can be regarded as having high confidence in terms of accuracy.

#### Conserved Sequences

When there are effector candidates that are known to belong to conserved sequence families, their identification or categorisation can be determined by sequence alone. Template-based modelling predictions for these effector candidates would be expected to match specific known effector structures as reference templates if they are available, but not in this case. The effector candidates belonging to conserved sequence families are Avr-Pita, Cgfl, MoCDIP4 and Msp1 (indicated by the effector name in italics in Table 5). The list of effector candidates in this category overlaps with the use of a single template. Avr-Pita and Cgfl have RaptorX scores of 246 and 337, respectively. They matched templates 1eb6A and 4k90A, respectively, and both of these templates are enzymes that are known to be highly conserved.

Modelling of Avr-Pita used the structure of deuterolysin (PDB structure 1eb6A) from *Aspergillus oryzae*. Deuterolysin is an enzyme that catalyses the preferential cleavage of bonds with hydrophobic residues of substrate residue P1. It is a microbial, zinc-containing metalloprotease that exhibits some similarity to thermolysin [43]. Pfam classified it as a deuterolysin metalloprotease m35 family domain, whilst CATH indicated that it is a collagenase domain, and SCOP2 indicated that it is a fungal zinc peptidase domain.

Cgfl is a fungalysin metalloprotease secreted by *Colletotrichum graminicola* that causes maize anthracnose [44]. Fungalysin catalyses the hydrolysis of extracellular matrix proteins, elastin and collagen, and also acts as a virulence factor. Currently, there are no experimental structures of these effector candidates, but a 3D model structure of Cgfl using Phyre2 has been published [44]. Since Cgfl is highly conserved, their predicted model is similar to ours in terms of the basic fold.

#### Synthetic Constructs

The modelling of a number of effector candidates used synthetic constructs obtained from the PDB. Effector candidates PGTG_08638 and VdSCP7 were thus modelled using PDB structures 5cwhA and 5cwfA as templates, respectively (indicated by reference templates in italics in Table 5). Both templates are de novo designed helical repeat proteins DHR14 and DHR8, respectively [45].

#### Match with Any Proteins in the PDB

**Fig. 5:**
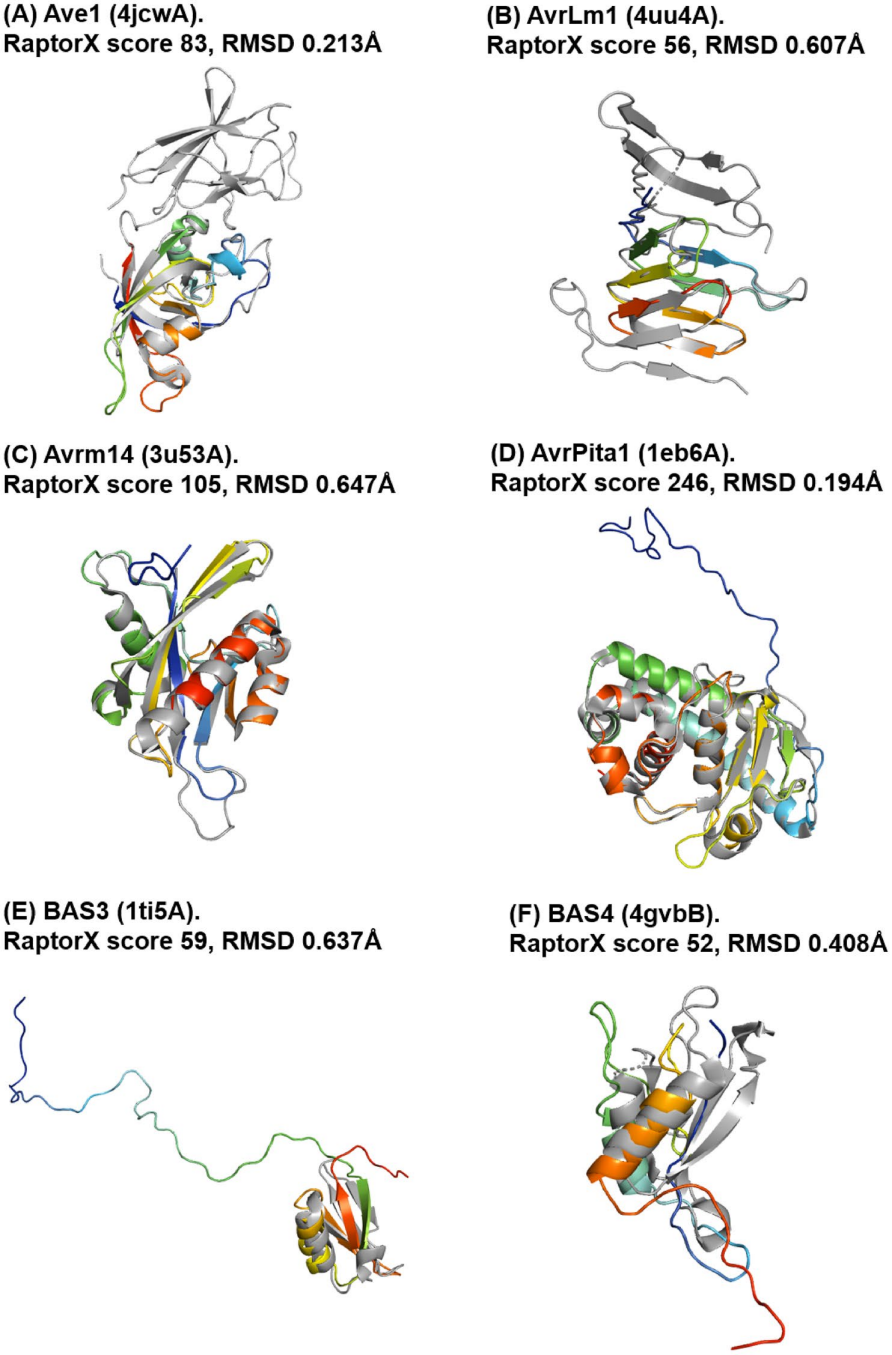

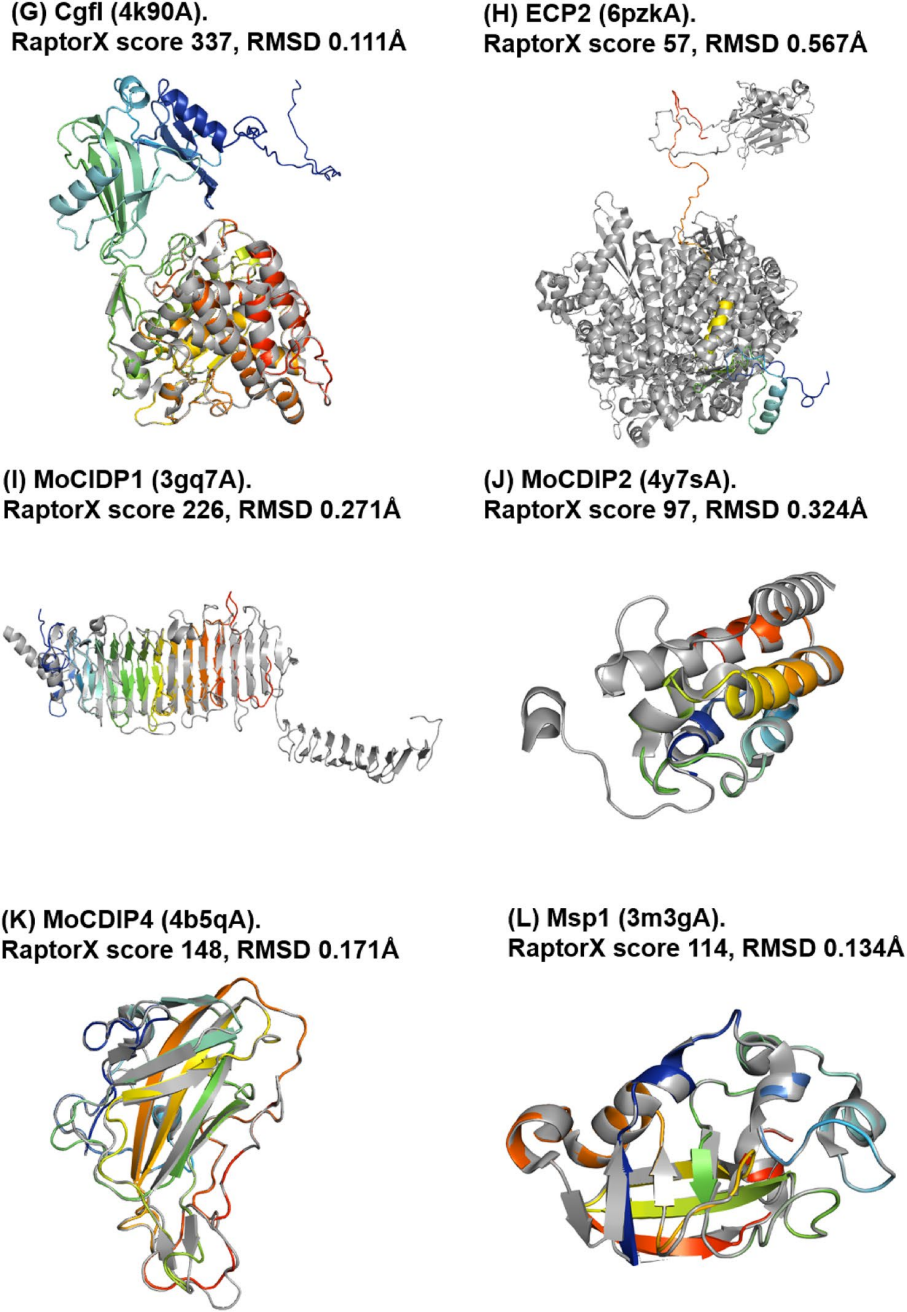

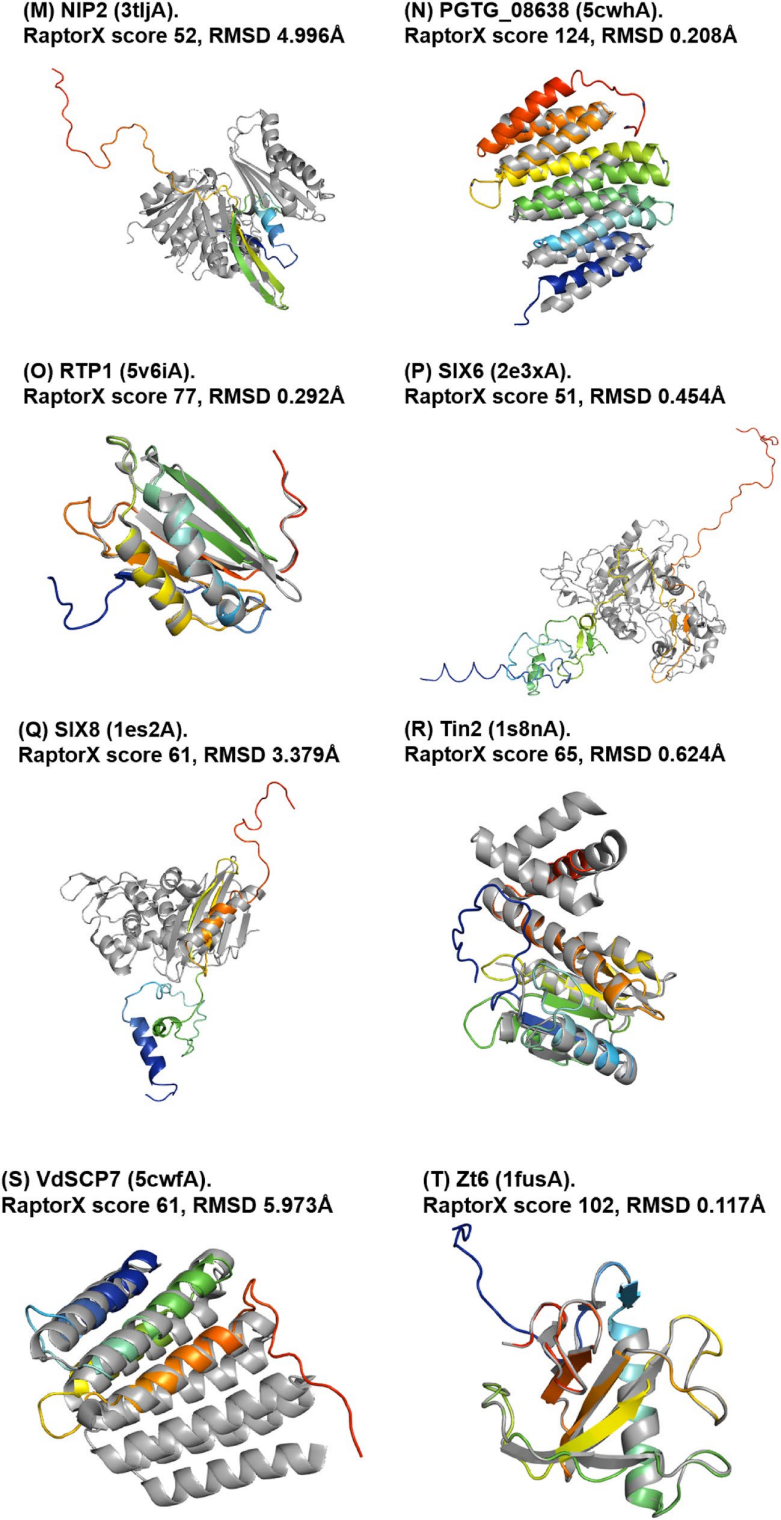
Predicted models of phenotypically validated effectors derived using templates matching any proteins in the PDB and exhibiting RaptorX scores above 50. The Cα–RMSD of the superimposition of the RaptorX model with the reference template structure (in grey) is reported

This corresponds to predictions of effector candidates that do not fall into any of the previous categories. Overall, 20 effector candidates were modelled with RaptorX scores above 50 (Table 5). The predicted models used various template structures from the PDB. None have a match with the same template, reflecting the wide diversity in the sequence of the effector candidates. Only models with a RaptorX score of above 50 are shown in Fig. 5, whilst the remaining can be found in Fig. S8 in SI. The templates used to model the effector candidates are described in more detail in Table 6 and Table S6 in SI.

**Table 6:** Description of the top-ranked templates used in RaptorX modelling of phenotypically validated effector candidates with RaptorX score above 50

Effector name	Template name (PDB ID)	Classification/function/location	Species	Fold
Ave1	Expansin (4jcwA)	Sugar binding protein	*Clavibacter michiganensis*	Beta
AvrLm1	LptA homologous periplasmic component of lipopolysaccharide transport device (4uu4A)	Lipopolysaccharide binding/transport protein, periplasmic space	*Pseudomonas aeruginosa*	Beta-jellyroll
AvrM14	Human Ap4A hydrolase (3u53A)	Hydrolase, GTP binding, ATP biosynthetic process	*Homo sapiens*	Nudix hydrolase family
Avr-Pita	Deuterolysin (1eb6A)	Metallopeptidase activity, proteolysis	*Aspergillus oryzae*	Fungal zinc peptidase
Bas3	Plant defensin 1 (1ti5A)	Plant protein, insecticidal activity	*Vigna radiata*	Knottin like
Bas4	Virally encoded antifungal protein, KP6 heterodimer (4gvbB)	Hydrolase	*Ustilago maydis virus P6*	Killer toxin
Cgfl	Extracellular metalloproteinase (4k90A)	Zinc ion binding, fungal peptidase, extracellular space	*Aspergillus fumigatus*	Cystatin-like
Ecp2	Respiratory syncytial virus polymerase L protein (6pzkA)	RNA-directed 5-3 RNA polymerase activity, transcription/ host cell cytoplasm	*Human respiratory syncytial virus A2*	Helical bundle
MoCDIP1	Bacteriophage Phi29 gene product 12 N-terminal fragment (3gq7A)	Viral protein	*Bacillus virus phi29*	Beta-strands
MoCDIP2	CFEM protein Csa2 (4y7sA)	Haem binding, extracellular membrane protein	*Candida albicans*	Helical basket fold
MoCDIP4	Lytic polysaccharide monooxygenase GH61D (4b5qA)	Metal ion binding/extracellular region	*Phanerochaete chrysoporium*	Mainly beta, distorted sandwich
Msp1	Sm1, elicitor of plant defence responses (3m3gA)	Metal ion binding/extracellular region	*Trichoderma virens Gv29-8*	Beta barrel
NIP2	tRNA:m^2^ G6 methyltransferase Trm14/TrmN (3tljA)	Methyltransferase activity, methylation/cytoplasm	*Pyrocossus furiosus DSM 3638*	Rossmann fold
PGTG_08638	De novo designed helical repeat protein DHR14 (5cwhA)	Synthetic construct	Synthetic construct	Tandem helical repeats
RTP1	Glycan binding protein Y3 (5v6iA)	Sugar binding protein	*Coprinus comatus*	αββ Sandwich
Six6	Viper venom metalloproteinase (2e3xA)	Metal ion binding/extracellular region	*Daboia ruselli*	Hook spanner wrench-like
Six8	DD-transpeptidase/penicillin binding protein (1es2A)	Peptidase activity, proteolysis/extracellular region	*Streptomyces sp. K15*	3-Layer αββ sandwich
Tin2	Putative transcriptional antiterminator, Rv1626 (1s8nA)	RNA binding, phosphorelay signal transduction system/plasma membrane	*Mycobacterium tuberculosis*	Helical bundle
VdSCP7	De novo designed helical repeat protein DHR8 (5cwfA)	Synthetic construct	Synthetic construct	Armadillo like helical
Zt6	Ribonuclease f1 (1fusA)	Ribonuclease T1 activity	*Fusarium vertisillioides*	Alpha beta roll

Modelling of the Ave1 effector used the crystal structure of expansin from *Clavibacter michiganensis* as template. Expansins typically have roles in promoting the loosening of cell membranes. The protein in this structure is found in a complex with cellopentaose at the N-terminal amino acid range 116–202 and contains four disulfide bridges. Based on Pfam analysis, the structure of Ave1 overlaps with the two domains of expansin: in the lytic transglycolase domain (in the range 2–99) and the C-terminal expansin domain (in the range 110–183). At the same time, the amino acid range 1–104 correlates with the rare lipoprotein A (RlpA)-like domain according to CATH. Ave1 is predicted to have a fold superfamily of a six-stranded double-psi β barrel (DPBB) located in a conserved region [46].

Modelling of the AvrLm1 effector used as template the crystal structure of LptH, which is a component of *Pseudomonas aeruginosa* transport device [47]. AvrLm1 overlaps with half of the protein structure of LptH (64 out of 175 residues). Both Pfam and CATH indicate the presence of a LptA/LptD N-terminal domain lipopolysaccharide transport protein in the amino acid ranges 34–146 and 26–170, respectively, for each database.

Modelling of the AvrM14 effector used as template the crystal structure of human asymmetrical diadenosine tetraphosphate hydrolase (Ap4A), which exhibited high structural similarity when superimposed, with a Cα–RMSD of 0.647 Å. Pfam analysis shows that Ap4A consists of a NUDIX hydrolase domain in the amino acid range 12–137, and pyrophosphohydrolase in the range 4–147. These findings are similar to those with CATH and SCOP2b. Ap4A hydrolase is an enzyme found in all living cells and which cleaves the phosphate chain at the fourth phosphate from the bound adenosine moiety. It has a αβα-sandwich architecture, which is common in the NUDIX fold. Ap4A has been proposed to be an intracellular signal molecule that regulates gene expression at transcriptional level and which may play a role in invasion in bacteria [48].

Apoplastic cell death-inducing proteins (CDIPs) have been identified in fungi and oomycetes [49], and there are five well-characterised CDIPs, MoCDIP1 to MoCDIP5, that each cause blast disease in rice [50] but appear to be functionally unrelated. Modelling of the MoCDIP1 effector used as template the crystal structure of the bacteriophage Phi29 gene product 12, N-terminal fragment, which is involved in the autocatalytic assembly mechanism of a bacteriophage tail spike. Pfam analysis shows that MoCDIP1 belongs to the pectate lyase superfamily protein (in the amino acid range 22–251). CATH indicates that it is a single stranded right-handed β-helix, pectin lyase-like enzyme. This family of proteins possesses a β-helical structure like pectate lyase, and this family is closely related to glycosyl hydrolase family 28. Pectate lyases are enzymes involved in plant cell wall degradation and are produced by plant pathogens and plant-associated organisms. The predicted model for MoCDIP1 has a nearly complete fit to the first domain of bacteriophage Phi29 gene product 12 at the N-terminus.

Unlike MoCDIP1, MoCDIP2 belongs to a family of common fungal extracellular membrane (CFEM)-containing proteins involving in host-fungi interactions [51]. MoCDIP2 has a large number of homologues in the sequence genomes of *M. oryzae* and other organisms. Modelling of the MoCDIP2 effector used the crystal structure of CFEM protein Csa2 from *Candida albicans* as its template, a secreted protein promoting haem uptake (haemophore) that scavenges iron to deprive the host [52].

Modelling of the MoCDIP4 effector used as template the structure of lytic polysaccharide monooxygenase GH61D from *Phanerochaete chrysosporium*. MoCDIP4 is a cell death-inducing effector [53]. Pfam analysis indicates that it corresponds to auxiliary activity family 9, AA9 (formerly GH61) domain, and CATH indicates an enzyme accession EC1.14.99.54, which corresponds to lytic polysaccharide monooxygenase. AA9 is a copper-dependent oxidative enzyme.

Modelling of Msp1 used as template the structure of Sm1, an elicitor of plant defence responses from *Trichoderma virens*. Pfam analysis indicates that Sm1 consists of a cerato platanin (CP) domain, whilst CATH indicates that it is a RlpA-like domain. The CP family of proteins includes the phytotoxin CP produced by *Ceratocystis platani*, which causes canker stain. CP occurs in the cell wall of fungi and is involved in host–pathogen interactions, inducing both cell death and phytoalexin production and the N-terminal region resembles cerato-ulmin, a hydrophobin [54].

Modelling of the RTP1 effector used as template the structure of glycan binding protein Y3 from the mushroom *Coprinus comatus*, resulting in high structural similarity when superimposed, with a Cα–RMSD of 0.292 Å. Y3 is a 130-amino acid long cytotoxic protein that possesses a unique mode of glycan binding and specificity, as well as anti-leukemic activity [55].

Tin2 in *Ustilago maydis* stabilises the maize protein kinase TTK1, which regulates anthocyanin biosynthesis [56], resulting in the reduction of host lignification to support fungal spread during infection. Modelling of the Tin2 effector used as template the crystal structure of Rv1626 from *Mycobacterium tuberculosis*, with high structural similarity between Tin2 and Rv1626. Both Pfam and CATH analyses indicate that the modelled Tin2 has a response regulator receiver domain in the amino acid ranges 16–126 and 11–142, respectively. A response regulator domain is involved in bacterial two-component systems, and it consists of an unique helix-turn-helix motif that can be observed in the predicted model (Fig. 5R), and which is required for DNA binding [57]. SCOP identified a CheY-like domain, which is also a member of response regulator family [58].

Biotrophy-associated secreted 3, Bas3 is an effector secreted by *M. oryzae* that accumulates in the biotrophic interfacial complex (BIC) and which might be involved in cell-to-cell movement of invasive hypha [59]. Modelling of the Bas3 effector used as template the structure of plant defensin *Vigna radiata* (mung bean). To our knowledge, there are no studies that relate Bas3 with plant defensins or toxins except a HMM–HMM-based sequence clustering study where effector Bas3 and its clustered homologues were putatively functionally annotated as similar to scorpion knottin toxins [3].

Other predicted models include effector candidates that are categorised as enzymes that contribute to virulence and which includes candidates Bas4, Ecp2, Six6, Six8 and Zt6.

#### No Match to Any Templates (i.e. Candidates for Non-template-Based Modelling)

A total of 24 out of the 66 experimentally validated effector candidates could not be modelled using template-based modelling and are thus candidates for non-template-based modelling (rows highlighted in grey in Table 5). Sequences with no match to any of the known effector structural families and to any protein structure in the PDB database have been used in ab initio modelling in a separate study [60]. These phenotypically verified effectors are Avr4e, Avra1, AvrLm11, AvrLm6, AvrPi9, Bas107, Bas162, Ecp4, eff1-1, MoCDIP3, NIP3, Pep1, PpEC23, Pst2, PstSCR1, Pwl1, See1, Six1, Six2, Six4, Six5, Six7, SPD10 and Tox1.

### Comparison with Other Template-Based Modelling Programs

**Fig. 6:**
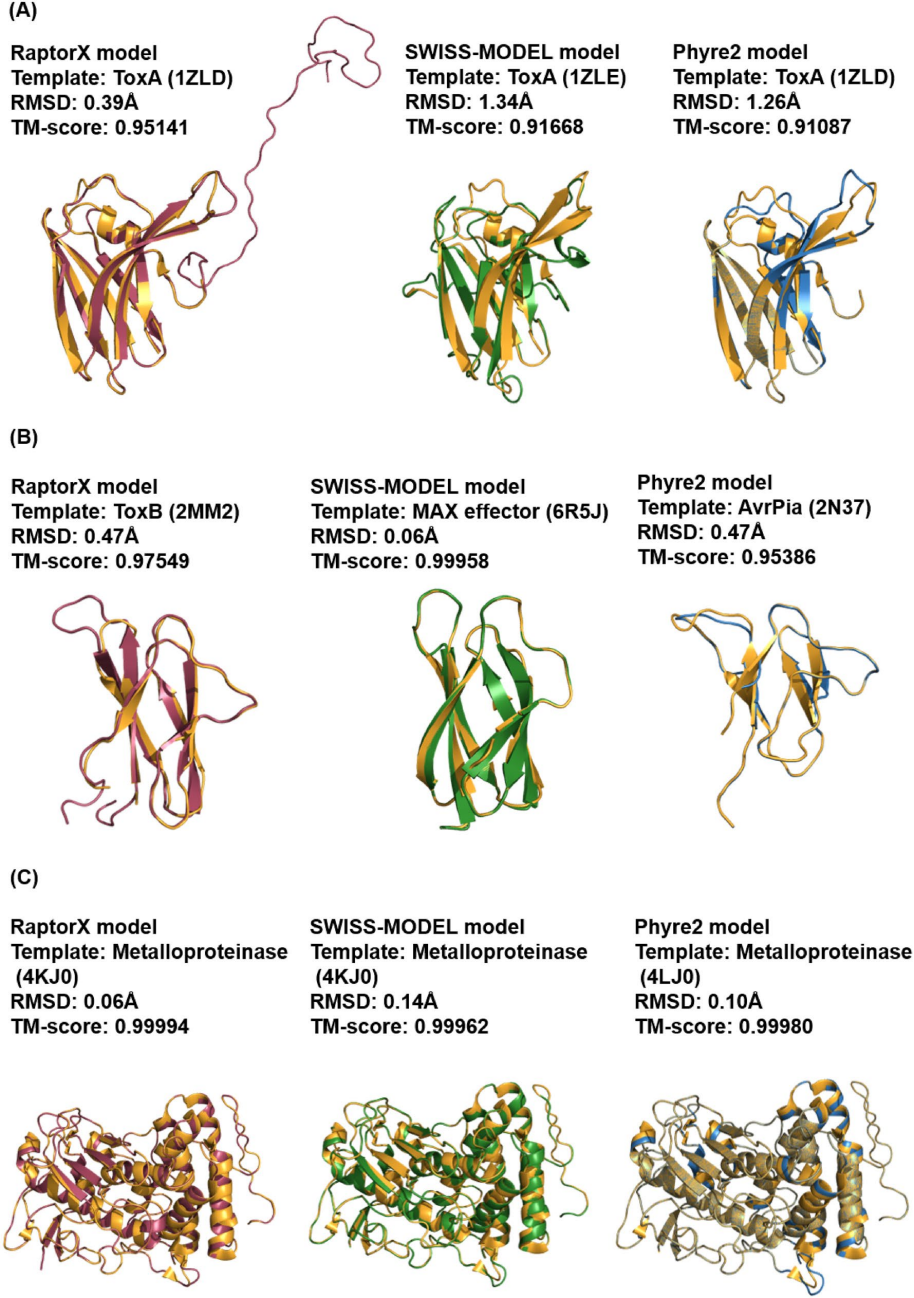
Comparison of template-based models with the top TM-score for ToxA-like (**A**), MAX-like (**B**) and phenotypically validated (**C**) candidates, modelled using RaptorX (maroon), SWISS-MODEL (green) and Phyre2 (blue) superimposed with their respective templates (gold) and the corresponding Cα–RMSD and TM-score values are included

The models predicted by RaptorX were compared with those predicted by other commonly used template-based modelling programs, in this case SWISS-MODEL and Phyre2. In the modelling of ToxA-like sequences, RaptorX was able to model all sequences (Fig. 6A; Table S5 in SI) using the ToxA effector (PDB structure 1zld) as the template structure. The best TM-score was 0.95141 for p2fl_ENH98532.1 and the lowest was 0.66836 for p0de_mRNA10272. These values correlate with their respective Cα–RMSD values of 0.39 and 0.84 Å.

In the modelling of ToxA-like sequences using SWISS-MODEL, four different templates were used, with the ToxA effector template (PDB structure 1zle) used in most cases, followed by SAP-1 (PDB structure 1bc8), which is a member of the Ets transcription factor, synaptotagmin-like protein (PDB structure 3bc1) and type 9 secretion system component (PDB structure 6h3j). TM-scores ranged from the highest value of 0.91668 for p2fl_ENH98532.1 to the lowest value of 0.16959 for p09v_mRNA10419. These values correlate with their respective Cα–RMSD values of 1.34 and 2.55 Å. Similar to RaptorX modelling, the model with the top TM-score for SWISS-MODEL is also p2fl_ENH98532.1, with a shared TM-score of 0.95141 with p2fk_EMD96331.1. The reason for a very low TM-score using SWISS-MODEL modelling is likely to be the use of a non-ToxA-like effector template for some of the models. In the case of models that use ToxA effector template, SWISS-MODEL selected PDB structure 1zle instead of 1zld, with the only difference being the crystal forms [61]. PDB structure 1zld has a better resolution of 1.65 Å compared to a resolution of 1.90 Å for structure 1zle; however, the residue and atom count is higher in structure 1zle, which might be the reason why it was selected as the top template by SWISS-MODEL. In the case of Phyre2 modelling, 13 different templates were used, with the ToxA effector template (PDB structure 1zld) being applied in the modelling of three of the candidate models, with two of them (p2fl_ENH98532.1 and p2fk_EMD96331.1) sharing the best TM-score of 0.91087. The lowest TM-score was 0.04336 and is also the lowest TM-score value amongst the three protein modelling programs compared. Cα–RMSD ranged from 0.23 to 5.11 Å.

When comparing the three protein modelling approaches, RaptorX had the highest usage of the ToxA effector structure (PDB structure 1zld) as template for the majority of the ToxA-like effector candidate models (Table S5A in SI): 34/34 for RaptorX, 31/34 for SWISS-MODEL and 3/34 for Phyre2. This relates to the efficiency of the programs during template ranking and selection (see Discussion). The TM-score of the top models for these three programs was different even though a similar ToxA effector template (PDB structure 1zld/e) was used. A slightly decreasing trend in the best TM-score values was observed, with a TM-score of 0.95141 for RaptorX, followed by 0.91668 for SWISS-MODEL and 0.91087 for Phyre2, showing that the RaptorX top model has the closest resemblance to the ToxA effector template compared to SWISS-MODEL and Phyre2 (Fig. 6A). The lowest TM-score values also followed a descending trend for RaptorX, SWISS-MODEL and Phyre2, with 0.66836, 0.16959 and 0.04336, respectively. This shows that RaptorX can model better a significant structural fold with a TM-score above 0.5 compared to SWISS-MODEL and Phyre2. This relates to the use of a different types of template in both SWISS-MODEL and Phyre2 modelling, as discussed later.

In the modelling of MAX-like sequences, RaptorX used 38 different types of templates with ToxB (PDB structure 2mm2) being the more common one (7 out of 55), followed by template Avr1CO39 (PDB structures 2myv and 5zng) (Table S5B in SI). TM-scores ranged from the highest value of 0.97549 for M.BR29.EuGene_00088411 to the lowest value of 0.04478 for p0de_mRNA10272. This correlates with their respective Cα–RMSD values of 0.47 and 4.99 Å. Similar to RaptorX, modelling of MAX effector sequences using SWISS-MODEL utilised a variety of 43 template structures. As with RaptorX, the more common template used by SWISS-MODEL was ToxB (PDB structures 2mm0/2), which was applied in the modelling of 6 out of 55 of the candidates. TM-scores ranged from the highest value of 0.99958 for M.TH16.EuGene_00135161 to the lowest value of 0.03779 for MGG_16058. This again correlates with their respective Cα–RMSD values of 0.06 and 2.39 Å.

Similar to RaptorX modelling of ToxA-like sequences, the model with the top TM-score was not the one with the top TM-score when using SWISS-MODEL (M.BR29.EuGene_00088411). The top models had a TM-score of 0.97549 in the case of RaptorX and 0.75221 in the case of SWISS-MODEL, even though the same ToxB effector template structure was used. In Phyre2 modelling, 47 template structures were used, with Avr1CO39 (PDB structure 2myv) being the template structure used in the majority of cases. The highest TM-score was 0.95386 for M.TH16.EuGene_00040131 and the lowest was 0.03266 for M.TH16.EuGene_00099371. The Cα–RMSD values ranged from 0.47 to 1.59 Å.

When comparing the three modelling methods, RaptorX exhibited the highest usage of MAX effector structures as template for the majority of the structurally unconfirmed effector models: 19/55 for RaptorX, 17/55 for SWISS-MODEL and 11/55 for Phyre2. This again relates to the efficiency of the programs during template ranking and selection, similarly to the modelling of ToxA-like effector sequences. However, in the predicted MAX effector models, there was no decreasing trend such as that described above for ToxA-like models when a similar MAX template was used for modelling. For example, in the case of M.BR29.EuGene_00085071, the ToxB structure (PDB structure 2mm2/0) was used as template, with resulting TM-scores of 0.81525, 0.83773 and 0.83949 with RaptorX, SWISS-MODEL and Phyre2, respectively. A similar situation was observed with other effector candidates (Fig. S6 in SI): M.BR29.EuGene_00106461, M.BR29.EuGene_00119491, M.BR29.EuGene_00119511, M.TH16.EuGene_00040131, M.TH16.EuGene_00106621 and M.TH16.EuGene_00135161. In these cases, Phyre2 models provided the best TM-score values, probably due to the use of a more updated PDB database during template selection.

When modelling experimentally validated effectors, models that were involved in benchmarking were omitted from this analysis. In the case of modelling using RaptorX, TM-scores ranged from the highest value of 0.99994 for Cgfl to the lowest value of 0.07516 for MoHEG13. These correlates with their respective Cα–RMSD values of 0.06 and 0.72 Å. In the case of modelling using SWISS-MODEL, the TM-scores ranged from the highest value of 0.99962 for Cgfl to the lowest value of 0.01960 for PstSCR1, correlating with their corresponding Cα–RMSD values of 0.06 and 2.85 Å. In the case of modelling using Phyre2, the highest TM-score was 0.99980 for Cgfl and the lowest was 0.01459 for AvrA13, with the latter being the lowest TM-score obtained amongst the three modelling programs. Cα–RMSD values ranged from 0.10 to 0.13 Å.

In the case of RaptorX, 24 effector candidates could not be processed with template-based modelling due to the lack of suitable experimental structures that could be used as templates (Tables S4, S5C in SI). A similar situation was observed for eight effector candidates when using SWISS-MODEL and with one candidate using Phyre2. Pst2 was the only effector candidate for which no suitable structural template could be identified in any of the modelling programs. Whilst modelling with Phyre2 resulted in the lowest number of candidates that had no suitable template, it also provided the highest number of effector models (18) with TM-score values below 0.0, indicating its low stringency in template selection and scoring compared to RaptorX (two models) and SWISS-MODEL (five models). Sequences with no appropriate structural template would thus be suitable for non-template-based (ab initio) modelling.

## Discussion

Separate topics related to the above-described template-based modelling studies are discussed below: (1) important factors affecting template-based modelling of fungal effectors, (2) benchmarking of template-based modelling of fungal effector proteins, (3) methods for sequence-based prediction of effector candidate sequences, (4) modelling of phenotypically validated fungal effector proteins, and (5) comparison of predictions made by template-based modelling programs.

### Important Aspects Involved in Template-Based Modelling of Fungal Effector Proteins

Our findings suggest that there are a number of factors that may affect the quality of the predicted structural models of fungal effector candidates that can be accounted for: the use of scoring metrics to assess structural similarity, the choice of template and the quality of the target–template alignment, the number of templates used to model a single structure and the presence of multiple domains in the target sequence.

Let us first focus on the use of specific scoring metric for the assessment of structural similarity between predicted models and the reference template structure. In this approach, TM-score is clearly preferable to Cα–RMSD because the latter does not give an accurate assessment of the structural similarity between the modelled and template structures. The TM-score used in this study is based on TM-align, which is sequence-independent as it first identifies an optimal alignment [33]. A good example of this can be seen in the phenotypically validated effector candidates, where the best model of MoHEG13 has a TM-score of 0.07516, indicating poor agreement in fold structure and, yet, the Cα–RMSD is 0.72 Å, suggesting high similarity with the reference template (Fig. S8M in SI). In this example, there is clearly no visible protein fold in the model and thus no significant match to the template structure, except for a loop region that aligns well to a small portion of the reference structure. This suggests that TM-score is a more reliable metric compared to Cα–RMSD when comparing fungal effector candidate models with their respective reference template structures, especially in cases where the length of the predicted model and template structure is different.

The second aspect to consider is the choice of template used in template-based modelling, which relies on the ranking generated by the quality or assessment of the target–template alignment. In the case of RaptorX, the likelihood of finding a good template is based on the use of a novel non-linear context-specific alignment potential and probabilistic consistency algorithm [22], since inaccuracies in template selection have a high impact on further model interpretations. The choice of template used correlates with the quality of the target–template alignment, which in turn affects template ranking. Template-based modelling thus also depends strongly on the quality of the target–template alignment. For example, in the modelling of MAX effector candidates, the candidate with the best RaptorX score, M.BR29.EuGene_00106461, has a score of 56 using AvrPia (PDB structure 2myw) as template. Four other templates were listed and ranked alongside the top-ranked, namely AvrPia (PDB structure 2n37), RadA intein (PDB structure 2lqm), YXIM BACsu (PDB structure 2o14) and a protein of unknown function (PDB structure 1yll). Amongst these five templates, the top two are MAX structures, AvrPia (PDB structures 2myw and 2n37), and PDB structure 2myw was chosen since it resulted in a higher RaptorX score of 56 compared to 43 for PDB structure 2n37. Other quality metrics were similar, with uGDT/GDT being 58/90 and uSeqID/SeqID being 29/45. An important difference, however, was observed in the *p*-value, being 3.07 × 10^−5^ with PDB structure 2myw and 4.60 × 10^−4^ with PDB structure 2n37, which is reflected in the sequence alignment (Fig. S9 in SI). Furthermore, differences in the latter indeed led to the predicted model being different when PDB structure 2n37 was used as template, with the protein model having a β-strand missing and incomplete β-strands (Fig. S9B in SI), reflecting that RaptorX weighs the alignment differently depending on the alignment score of the specific target–template used. This is crucial since fungal effector proteins tend to lack sequence conservation and identifying remote template sequences is important, especially in cases where there are no homologues with available experimental 3D structures. A profile entropy scoring method is applied in RaptorX to determine the best modelling mode, and this depends on the sequence profile, such that non-existence of a close template results in a low entropy score, and more weight will be given to structural rather than sequence information [22] in the modelling of the effector candidates. Target–template alignment depends on the optimal alignment used in threading, which involves the transfer of atomic coordinates from the template structure to the target sequence, as well as by the use of a non-linear alignment scoring function used for template ranking in RaptorX [22].

The third aspect that merits consideration is whether a single or multiple templates are used in the modelling of a single structure. By default, RaptorX uses multiple (up to five) templates to model a single structure [23]. Multiple template modelling is applied only if the model generated by multiple templates is better than the model generated from a single template, which applies to the majority of cases. However, we observed that there were several instances where only a single template was ranked and used for modelling of the phenotypically validated candidates, such as the first domain of effector MoCDIP2, indicating that the sampling of template selection prior to modelling had converged. This is also confirmed in the initial benchmarking studies, where the majority of the models were derived from a single template structure, as expected (Table 1; Table S4 in SI). The exceptions were Avr1CO39 (PDB structures 2myv and 5zng), AvrL567A (PDB structures 2opc and 2qvt) and AvrPia (PDB structures 5jhj and 2n37), where two different templates with similar structure were ranked for each model during benchmarking. Increased accuracy was observed with the use of a single template compared to multiple templates, since a reduction in accuracy can occur if one less accurate template is ranked amongst the top five templates. Depending on the target sequence, the use of multiple templates may provide better target coverage and better individual pairwise target–template alignments that can improve the final models. Multiple template threading is probably not beneficial in the case of limited sequence profiles, but may increase accuracy for models for which non-effector structures are used as templates (depending on the scores).

The fourth aspect to consider is the presence of multiple domains/domain parsing in the modelled structures (marked with an asterisk in Table S4 in SI), such as in the following seven effector candidates: AvrLm1, Ecp2, effl-1, MoCIDP2, MoCIDP4, Six6 and Six8. For all of these candidates, dual domains were observed within a single model, except for Six8, which has three domains. These domains were modelled independently of the overall structure based on the Pfam database, with an *E*-value of < 0.001 as cut-off to indicate an independent domain [22].

### Benchmarking of Template-Based Modelling of Fungal Effector Proteins

The first part of this template-based modelling study was conducted using sequences for which experimentally determined 3D structures are available (Table 1). Benchmarking was conducted to assess the reliability of the template-based modelling approach for predicting the structure of fungal effector proteins, as well as to validate the use of TM-score, Cα–RMSD and RaptorX score metrics. All effector templates were successfully modelled, including cases that we have categorised as difficult, such as effectors AvrPik and Avr1CO39, in non-template-based (de novo/ab initio) modelling of fungal effector proteins due to the presence of long-disordered loops [60]. Our findings using template-based modelling thus demonstrate its reliability compared to non-template-based approaches. Availability of a suitable template for structure modelling can indeed overcome the limitations of modelling protein regions that contain highly flexible loops or disordered regions. However, in the case of fungal effector proteins, the number of available experimental effector template structures remains limited, preventing the full application of template-based modelling to predict the structure of fungal effector protein candidates.

### Methods for Sequence-Based Identification of Effector Candidate Sequences

This study also provides validation of the accuracy and reliability of sequence-based bioinformatics approaches for predicted template matching prior to template-based modelling, such as in the case of ToxA-like and MAX-like effector candidates. Input candidate sequences had been pre-determined to belong to certain effector families even though, in general, template-based modelling could be applied to uncharacterised sequence candidates. The accuracy of template-based modelling relies on the input protein sequence, which in turn depends on the accuracy of the sequence-based bioinformatics approach applied for the prediction of those effector candidate sequences. This involves determining how close the predicted sequences are to a specific template that belongs to known effector families.

Bioinformatics sequence-based prediction of fungal effectors may utilise ‘remote homology’ (profile-based) approaches instead of conventional sequence homology searches due to the low sequence similarity of fungal effectors with other proteins [3, 11]. Based on the top template ranked in the template-based modelling of each effector candidate, we observed that sequence-based approaches used for predicting effector sequences were more accurate for ToxA-like candidates than MAX-like candidates, since the same structural template for ToxA (PDB structure 1zld) was used as the top template for all ToxA-like candidates. In the identification of ToxA-like candidates, a remote homology clustering approach (termed RemEff) using MMSeqs2, HMM-HMM searches and HHBlits had been applied in a previous study [3]. In addition, ToxA-like effector candidates are an easy target for template-based modelling because of the robust and efficient sequence-based bioinformatics approach implemented to predict these sequences. This is demonstrated by the success in modelling each candidate with high structural similarity to the experimentally determined ToxA effector (PDB structure 1zld).

In the case of the modelling of MAX-like effectors, the sequences of candidates that are homologues of AvrPia, Avr1CO39 and AvrPizt had been identified in a previous study using PSI-BLAST iterations in the genomes of *M. oryzae* and *Magnaporthe grisea* isolates [11]. This sequence-based prediction of MAX-like effector candidates led to half of the listed candidates having MAX templates used for structural modelling. The remaining candidates had templates that matched to non-MAX effectors (Table S3 in SI), creating uncertainty as to whether they are true MAX-like effector candidates. The structure-based modelling approach using RaptorX indicated that not all PSI-BLAST-predicted candidates were MAX-like effectors, due to the absence of suitable matching MAX-like templates. However, it is important to also consider the limitations of the template-based modelling approach, such that either downstream (determination of 3D structure) or upstream (effector activity) experimental validation is required. This also points to the challenge in predicting the structure of a sequence that does not have similarity with other protein sequences, including within MAX effector family.

The overall match of effector candidates to corresponding fungal effector family structural templates was found to be ~ 35% for MAX and ~ 64% for Tox-like. The identification of ToxA-like effector candidates using the RemEff [3] clustering method appears to be more advantageous and efficient compared to the profile-based iteration approach which was applied for the prediction of MAX effector candidates. Differences in the outputs of the two approaches depend somewhat on the availability of homologous sequences for creating sequence profiles at the time, the different selection and evolutionary histories of these two structural families, and the fact that the two studies are several years apart. Nevertheless, the application of sequence-based bioinformatics approaches for effector candidate prediction prior to template-based modelling is an important part in the design of a more accurate pipeline for the identification of fungal effectors.

### Comparison of Predictions Made by Template-Based Modelling Programs

The modelling predictions made using RaptorX (a threading-based program) were compared with those of other publicly available template-based modelling programs, in this case SWISS-MODEL (a comparative modelling program) and Phyre2 (a fold recognition program) to try to determine which approach is the best for predicting the structure of fungal effector candidates. Most template-based modelling programs search for evolutionary-related template protein structures to identify close and remotely related templates. Secondary and disordered structure prediction are also included in all programs, which could improve modelling predictions since most fungal effector proteins are known to possess regions with long-disordered loops [62]. The main difference in the above programs is their modelling approach, the use of different scoring metrics for the ranking of the top templates prior to modelling, and the strategy for target–template alignment.

Due to low sequence identity (< 30%) of effector candidates, such as is the case of ToxA-like and MAX effector candidates, threading would be expected to be the best protein modelling approach [63]. By contrast, comparative modelling would be expected to provide more accurate models for effector candidates with more conserved sequence families (> 30% sequence identity), such as RALPH and chitin-based. This relates to the underlying methods in these approaches, where more weight is placed on structural information in a threading-based approach to assist modelling, such as is the case of RaptorX [32], compared to comparative modelling, which relies more on sequence information. The following comparison likely reflects how these programs have been designed to work.

A different strategy in target–template alignment was observed in the modelling of the ToxA-like candidate p2fl_ENH98532.1 using ToxA (PDB structure 1zld) as template. All programs predicted a model with the best TM-score compared to other ToxA-like candidates. However, their TM-scores were not exactly the same, and the final model generated differed in the residue length even though the starting target sequence was of the same length of 143 amino acids. Depending on the algorithm for target–template alignment in each program, different alignment coverage was generated: RaptorX had 100% coverage, meaning that all residues were being counted in the alignment with the target sequence, whilst SWISS-MODEL had 80% coverage and Phyre2 had 68% coverage (Fig. S10 in SI). These differences in alignment coverage affected the length of the final structural model generated, as shown in Fig. 6A. In this case, it is clear that RaptorX is the preferred method since no residues were omitted from the final models regardless of the absence of favourable secondary structures in certain regions, or the absence of regions that may consist of disordered loops, especially at the N- or C-termini of the starting sequence. It is important to retain these regions in the final model for further studies, since they might have a functional role and adopt a given secondary structure (e.g. helix) during binding with other macromolecules or the internalisation of fungal effector proteins into host cells. Missing residues at the N/C-termini were also observed in other effector candidates modelled using SWISS-MODEL and Phyre2, which relates to the target–template alignment approached used in these two programs. By contrast, in RaptorX regions that do not align to any template region are subjected to domain analysis and, if there were no matches to any template, these regions are modelled as disordered depending on the type of residues. Consequently, obtaining a precise model of a disordered region requires a more complex modelling algorithm that is usually not applied in template-based modelling.

There is also an obvious trend in the predicted top TM-scores for ToxA-like effector candidate models. TM-scores reflect how close the models are to the reference template and, in the majority of cases, RaptorX models had the highest TM-scores, followed by SWISS-MODEL and Phyre2. The lowest TM-scores amongst the three programs also show that RaptorX models exhibit the highest ‘lowest’ TM-score compared to SWISS-MODEL and Phyre2. This reveals that the worst models produced by RaptorX are still acceptable compared to those produced by SWISS-MODEL and Phyre2. Another interesting observation relates to the number of phenotypically validated effector candidates that could not be modelled due to the lack of an available template. RaptorX had the highest number of candidates with 24, compared to SWISS-MODEL with 8 and Phyre2 with only 1. However, even though Phyre2 was able to model nearly all candidates, it produced the highest number of models with TM-scores below 0.0, making these models unusable for further analysis. This relates to the stringency of the choice of template used in RaptorX, which again depends on the quality of the template alignment and the scoring metric. If a template can be found for a candidate, a target–template alignment will be created, but the scoring generated from the alignment might not satisfy the cut-off in RaptorX, which is stricter than the other programs. This results in a higher level of reliability in the choice of template and overall quality of the model of fungal effector candidates predicted by RaptorX.

Overall, RaptorX was found to be the most successful program at predicting the structure of fungal effector candidates, followed by Phyre2 and SWISS-MODEL. The success of RaptorX might be attributed to the accuracy of the template selection step, which uses customised weights and a non-linear scoring function for template ranking, an approach not used by the other programs used in this study. Phyre2 is a fold recognition-based program that takes advantage of the finite number of protein folds in nature, exploiting secondary structure information in modelling; however, it is limited in its search space because not all folds will have an exact match when combined together. SWISS-MODEL benefits from the speed of its sequence alignment step prior to modelling; however, the quality of the resulting target–template alignment decreased to below 40% sequence identity, such as in the case of most fungal effectors, which affected the final predicted models.

We can conclude that the choice of method used in template-based modelling of fungal effector protein candidates is important for obtaining a model that is reliable for categorisation into certain effector structural families based on the underlying choice of template. This also shows that the application of template-based modelling to the structure of different effector structural families depends on the availability of templates for that specific effector family.

### Modelling of Phenotypically Validated Fungal Effector Candidates

Datasets of fungal effector proteins with experimentally verified virulence phenotypes were collected from the literature, with most of these lacking sequence homology, and having previously been applied as a positive training dataset for machine-learning-based effector prediction methods [28]. Since the input sequences are a mixture of different effector families (assumed based on lack of sequence homology), we tested whether template-based modelling could be used to validate and categorise a small proportion of them into known effector families, similar to the case of the ToxA-like and MAX structural families. In this study, three of the predicted models of these phenotypically validated effectors matched the RALPH (Avra13 and AvrPm2) and MAX (SPD7) effector families. The majority of predicted effector models had matches to other proteins in public databases, with a minority not having any matches to any templates (but making them suitable for non-template-based (ab initio) modelling [60]). RaptorX succeeded in predicting the structure of about half of all candidates.

Intriguingly, several structural models were derived from non-fungal effector templates, which suggested a common theme of cytotoxic and membrane-interacting functions that may have been broadly conserved across distant kingdoms of life. These models hint at the possibility of broadly conserved folds or structures not yet characterised within effector structural families, possibly suggesting similar functional roles in fungal effectors based on their structural similarity with proteins in the PDB. For example, the predicted structure of Bas3 matched to the cytotoxic plant defensin 1 (PDB structure 1ti5), which has a knottin-like fold and is known to have insecticidal activity [59]. Six6 also matched a cytotoxic viper venom/metalloprotease (PDB structure 2e3xA). Similarly the predicted structures of some effectors matched to structures with known membrane-degrading or modifying functions, including Ave1, which matched to the membrane-loosening expansin protein (PDB structure 4jcwA), and AvrLm1, which matched the lipopolysaccharide-binding/transport protein LptA (PDB structure 4uu4A). Several effectors also matched to structures with proteolytic functions, activities which are often associated with fungal effectors (Table 6; Table S6 in SI).

On the basis of the matching structural templates used during modelling, some of the models of phenotypically validated effector candidates were not observed to have the typical properties of fungal effector proteins, i.e. being small secreted proteins with less than 200 amino acids and high in cysteine content [4, 15]. Although not mutually exclusive with effectors, some predicted structures were annotated with other functions not typically linked with membrane disruption or cytotoxicity. These may still play crucial roles in the infection process but are less well understood, but their predicted structures could be used for the study of interacting proteins using molecular docking simulations or experimental methods to further support this. In this study, several effectors were shown to have matches with structural templates with uncertain virulence roles, such as was the case for candidates that have more than 200 residues: PGTG_08638, MoCDIP1, MoCDIP4 and Cgfl. The predicted structure of PGTG_08638 was derived on the basis of a synthetic construct template consisting of an α-helical fold (PDB structure 5cwh). The predicted structure of MoCDIP1 used as template the structure of a viral protein containing β-plated sheets (PDB structure 3gq7). The predicted structure of MoCDIP4 used as template an extracellular metal ion binding protein consisting of a β-strand sandwich fold (PDB structure 4b5q). The predicted structure of Cgfl used as template the structure of a zinc ion binding fungal peptidase containing an α-helical fold (PDB structure 4k90) (Fig. 5).

## Conclusions

Template-based structural modelling was applied to predict the structures of fungal effectors belonging to the well-defined ToxA-like and MAX effector families. This approach was extended to predict novel functional predictions based on structural homologies between other phenotypically validated effectors.

The threading-based program RaptorX was found to perform better in the modelling of the structure of fungal effectors compared to comparative modelling approaches, such as those in SWISS-MODEL and Phyre2. This appears to be due to the intrinsic limitations of comparative modelling methods when dealing with the low sequence identity that effectors exhibit with other proteins. Target–template alignment quality was found to be the most important aspect in obtaining an accurate predicted model.

Knowledge generated by this study can be applied to future effector identification. Template-based modelling has indeed the potential to be incorporated into the pipeline of fungal effector protein discovery. This work identified the best parameters required for the generation of an effector identification pipeline using template-based modelling, which is aided by the fact that the constituent modules in RaptorX may be customised in an application focused on fungal effector protein candidates. This approach, however, may not be as high throughput as sequence-based approaches due to the nature of the physicochemical properties of protein structures; however, it could be a downstream add-on to a sequence-based bioinformatics approach, filtering ‘true’ effectors from candidate sequences that might include false positives.

Template-based modelling is an approach that should be considered before applying ab initio modelling because it is in general more reliable in spite of its limitations. The challenges that might arise during the application of template-based modelling include the use of template structures that are not related to effectors and/or have non-relevant functions in phytopathogenic fungi, as well as the limitations of template-based methods, which can become unreliable when there are no homologues with experimentally solved structures. The latter certainly applies to the case of fungal effector proteins, which can result in an absence of models being generated and, consequently, necessitating a different approach, such as ab initio modelling.

Since the sequences of effector protein candidates were obtained from the literature and public databases, this study contributes to the validation of candidate effector families defined by effector sequences and also provides more information regarding the structural folds that may be present in these validated effectors. This may allow the discovery of new fungal effector protein structural families or folds. In this study, several effector candidates were found to have a match with template/reference structures outside of known fungal effector families, and based on this study, all 33 ToxA-like candidates are considered ‘true’ ToxA-like effectors (structural-based), whilst 19 of the MAX-like candidates are structurally homologous MAX effectors. Amongst the experimentally validated effector candidates, effectors AvrA13 and AvrPm2 were found to belong to the characterised RALPH effector family and effector SPD7 to the MAX effector family. The remaining phenotypically validated effector candidates matched with templates of other non-effector-like proteins, some of the effectors modelled were found to exhibit folds that have not yet been identified amongst effector structural families. Compelling examples included effector Bas3 which has a knottin/defensin-like fold similar to plant defensin 1, cytotoxic peptides with integral roles in the plant immune response. Additionally, the Six6 effector was structurally homologous to viper venom metalloproteinase, a cytotoxic peptide from the animal kingdom. These and other examples of known fungal effectors with cross-kingdom structural homology observed in this study may represent the first reports of ancestrally conserved structural folds with common roles in cytotoxicity, cell membrane disruption and proteolysis.

### Supplementary Information

Below is the link to the electronic supplementary material.

Supplementary file1 (PDF 4578 kb)

## Data Availability

The data supporting the findings of this study are available within the article and its supplementary material. The authors would be happy to provide any additional data upon request.
