# Supplementary material for: Template-Based Modelling of the Structure of Fungal Effector Proteins
Source: Mol Biotechnol. 2023 Mar 20;66(4):784–813. doi: 10.1007/s12033-023-00703-4 (PMC11043172; doi:10.1007/s12033-023-00703-4)
Supplement: Supplementary file 1 — Supplementary file1 (PDF 4578 kb) [file 12033_2023_703_MOESM1_ESM.pdf]

Template-based modelling of the structure of fungal effector proteins

Authors: Lina Rozano[1,3], Darcy A.B. Jones[2,3], James K. Hane[2,3], Ricardo L. Mancera*[1,3]

[1] Curtin Medical School, Curtin Health Innovation Research Institute, GPO Box U1987, Perth WA 6845, Australia.

[2] Centre for Crop and Disease Management, School of Molecular and Life Sciences, Curtin University, GPO Box U1987, Perth WA 6845, Australia.

[3] Curtin Institute for Computation, Curtin University, GPO Box U1987, Perth WA 6845, Australia.

*Corresponding author: Ricardo L. Mancera; E-mail: R.Mancera@curtin.edu.au

Supplementary Information

Fig. S1 ToxA-like effector candidate mature sequences in FASTA format.

>p05c_mRNA16607
TALSNMDVREQGCFTHILPVLRINRDNRRDIQGDLDARRSIEWNPNRNVRAIIDFSSTTTRNRQLRYQITNRSQLFGAAIIFSNYQ
DTITGTVRETIRVPIGRATGMSLSPTPTTGAGCIVLPRLAGTWYAQLE
>p05d_mRNA9122
TALSNMDIREQGCFTHILPVLRINRDNRRDIQGDLAARRSIEWNPNRNVRAIIDFSSTATRNRQLRYQITNRSQLFGAVIIFSNYQD
TITGTVRETIRVPIGRATGMSLSPTPTTGAGCIVLPRLDGTWYAQLE
>p05e_mRNA13670
TALSPLDNSRQGCFTHIIPVLRINRNNRRDIQGDLDARRSIEWNPNRNVRVIIDFSSTTTRNRQLRYQITNRSQLFGAAIIFSNYQD
TITGTVRETIRVPIGRATGMSLSPTPTTGAGCIVLDRLDGTWYAQLE
>p05g_mRNA17320
TALSNMDVREQGCFTHILPVLRINRDNRRDIQGDLDARRSIEWNPNRNVRAIIDFSSTTTRNRQLRYQITNRSQLFGAAIIFSNYQ
DTITGTVRETIRVPIGRATGMSLSPTPTTGAGCIVLPRLDGTWYAQLE
>p05k_mRNA3392
TALSNMDVREQGCFTHILPVLRINRDNRRDIQGDLDARRSIEWNPNRNVRAIIDFSSTTTRNRQLRYQITNRSQLFGAAIIFSNYQ
DTITGTVRETIRVPIGRATGMSLSPTPTTGAGCIVLPRLDGTWYAQLE
>p05m_mRNA12409
TALSNMDVRRQGCFTHILPVLRINRDNRRDIQGDLDARRSIEWNPNRNVRAIIDFSSTTTRNRQLRYQITNRSQLFGAAIIFSNYQ
DTITGTVRETIRVPIGRATGMSLSPTPTTGAGCIVLPRLDGTWYAQLE
>p05n_mRNA11205
TALSNMDVRRQGCFTHILPVLRINRDNRRDIQGDLDARRSIEWNPNRNVRAIIDFSSTTTRNRQLRYQITNRSQLFGAAIIFSNYQ
DTITGTVRETIRVPIGRATGMSLSPTPTTGAGCIVLPRLDGTWYAQLE
>p09v_mRNA10419
APLSSSPELYEDSETGLYPRQPGQCRVINQDVPPQVDVQQVGRISIDYAIIHRYRRVYYPNNFLTVVIDARRLSRIHPFIDIEITNTE
SRNSAITLTRREFGSRTYNDIVSILVPKRIGENKGEMYICLQLFKLSGHFYISA
>p09v_mRNA9195
APLSQPAAHIQDHNNLFKRQYLSCFNTQPPNHVSSSNVGWVYVSRAIRDRNELEYQINNQVDFKVDASQLTEENRQVTITVKNF
ETNPILIVFARRRFRPAIIEEYDRFFVPRRVGAIAGEVTTCVHMRKISGEWYVA
>p09x_mRNA3934
VPQEKPQSVSRPSNDAIFERAALDCFATRPTGFLEPDAPQADITGAINARAQVRYPIGAHLSVDVDARGLTRYNLQVVIAITYTGT
EGGVLATSRR
>p0dd_mRNA2255
GAVSYLDNRPTGCLANIFALLTINQIGRVDIQNEINRRTGIRRDFNGNINVNLDFSQTYEPLRFLRFSIQNRSQFISNALILSNYEDTL
IGSPRATIRISIPRAGGVAIGTAPSTYTGCIELPRLDGTWYAQLE
>p0de_mRNA10272
GVALPLDSRDTSCLANVFALLVINRVGRIDIQDQINNLNTWRRDFNNNINVNLDFTQSFEPLRFVRYSIRNTSQFISNALILSNYRD
TAVGTPRETIRIPI
>p1ap_mRNA3793
SPLEARQGCFTHIIPVLRINRNNRRDIQGDLDARRSIEWNPNRNVRVIIDFSSTTTRNRQLRYQITNRSQLFGAAIIFSNYQDTITGT
VRETIRVPIGRATGMSLSPTPTTGAGCIVLDRLDGTWYAQLE
>p1b1_EXF72942.1
TALSPLDNSRQGCFTHIIPVLRINRNNRRDIQGDLDARRSIEWNPNRNVRVIIDFSSTTTRNRQLRYQITNRSQLFGAAIIFSNYQD
TITGTVRETIRVPIGRATGMSLSPTPTTGAGCIVLDRLDGTWYAQLE
>p1bd_mRNA10016
TALSPPDNSSCFSHILPVLRINRDNRRDIQGDLDARRSIEWNPNRNVRVIIDFSSTTTRNRQLRYQITNRSQLFGAAIIFSNYQDTIT
GTVRETIRVPIGRATGMSLSPTPTTGAGCIVLDRLDGTWYAQLE
>p1bd_mRNA1147
TALSPPDNSSCFSHILPVLRINRDNRRDIQGDLDAHRSIEWNPNRNVRVIIDFSSTTTRNRQLRYQITNRSQLFGAAIIFSNYQDTIT
GTVRETIRVPIGRATGMSLSPTPTTGAGCIVLDRLDGTWYAQLE
>p1bi_OBR06575.1
LAVDKLDTRQNSCIGATYPVLSVNHLDRKEFQGEIDQRGSLGWLATPDVSVIFDFGATTPANPQLNFRILNKNSATWKAVILSNY
VDTITTPARETVRISIDAARKVGTDSIPTILDGCVQLPRLDGTWYVQMES
>p1bo_mRNA4951
TALSPPDNSAIGAILPVLRINRDNRRDIQGDLDARRSIEWNPNRNVRAIIDFSSTTTRNRQLRYQITNRSQLFGAAIIFSNYQDTITG
TVRETIRVPIGRATGMSLSPTPTTGAGCIVLPRLAGTWYAQLE
>p22r_EXK24251.1
AALPEESQLDTRTITESQALSIARRQVDRAHQTNFQWVIREEGRTDWNPNNHVHVLIDAQTTEFDHRLTVEITNRSQRFGNAVIL
SRYAGDTNTGPIQDQIRIPVGTAEGRTAAFVSRCIRLPSLQGTWWWQLEN
>p2fk_EMD96331.1
KPLSLNARELSLDGRELSLDARSVLHKRQGSCVSISRPANNPSINNIGQIDITSVVNGGPGMTWDLNNLVRVHVSREVDGSLSFD
WTNTGSANRLIVTEWNSSSSTSGNGYVLLGSYGLPTGTGNICFPVGSGQGRTWKMQLED
>p2fl_ENH98532.1
KPLSLNARELSLDGRELSLDARSVLHKRQGSCVSISRPANNPSINNIGQIDITSVVNGGPGMTWDLNNLVRVHVSREVDGSLSFD
WTNTGSANRLIVTEWNSSSSTSGNGYVLLGSYGLPTGTGNICFPVGSGQGRTWKMQLED
>p2fn_EUC44184.1
LSPRQGSCLSALSAPNPVNTIGRLSFQWAIDQRTQEAAQASWSPSNFVDFHMTVPTDQNVNVTVINRSSRSNAIILTNYQDTRTS
TARAQVRVAVPAVRRLGRTETCLYLPRLDGTWYFQLE
>p2fq_EUC36307.1

LEPRQGACLSSLVRPNPVNNVGRISFQRYIDEPRTTGERADASNLIDVHMTVPTNQNINVYVTNRSTRGNAIILTNFQDTLSTQPR
AQIRITVPGQTRANGVVRPGMASPCVHLPRLDGTWYFQVE
>p2g0_EFQ93895.1
GAVSYLDNRPTGCLANIFALLTINQIGRVDIQNEINRRTGIRRDFNGNINVNLDFSQTYEPLRFLRFSIQNRSQFISNALILSNYEDTL
IGSPRATIRISIPRAGGVAIGTAPSTYTGCIELPRLDGTWYAQLE
>p2g1_PZD05769.1
GVALPLDSRDTSCLANVFALLVINRVGRIDIQDQINNLNTWRRDFNNNINVNLDFTQSFEPLRFVRYSIRNTSQFISNALILSNYRD
TAVGTPRETIRIPI
>p2g2_PZC93680.1
GVALPLDSRDTSCLANVFALLVINRVGRIDIQDQINNLNTWRRDFNNNINVNLDFTQSFEPLRFVRYSIRNTSQFISNALILSNYRD
TAVGTPRETIRIPI
>p2g3_PZD24241.1
GVALPLDSRDTSCLANVFALLVINRVGRIDIQDQINNLNTWRRDFNNNINVNLDFTQSFEPLRFVRYSIRNTSQFISNALILSNYRD
TAVGTPRETIRIPIPRALAIGTPAPSTYVGCVELPRLDGTWYAQLE
>p2g4_PZD32416.1
GVALPLDSRDTSCLANVFALLVINRVGRIDIQDQINNLNTWRRDFNNNINVNLDFTQSFEPLRFVRYSIRNTSQFISNALILSNYRD
TAVGTPRETIRIPIPRALAIGTPAPSTYVGCVELPRLDGTWYAQLE
>p2g5_PZD46046.1
GVALPLDSRDTSCLANVFALLVINRVGRIDIQDQINNLNTWRRDFNNNINVNLDFTQSFEPLRFVRYSIRNTSQFISNALILSNYRD
TAVGTPRETIRIPIPRALAIGTPAPSTYVGCVELPRLDGTWYAQLE
>p2g6_PWO08528.1
GVALPLDSRDTSCLANVFALLVINRVGRIDIQDQINNLNTWRRDFNNNINVNLDFTQSFEPLRFVRYSIRNTSQFISNALILSNYRD
TAVGTPRETIRIPIPRALAIGTPAPSTYVGCVELPRLDGTWYAQLE
>p2g7_PZD04407.1
GVALPLDSRDTSCLANVFALLVINRVGRIDIQDQINNLNTWRRDFNNNINVNLDFTQSFEPLRFVRYSIRNTSQFISNALILSNYRD
TAVGTPRETIRIPIPRALAIGTPAPSTYVGCVELPRLDGTWYAQLE
>p2g8_PWO20795.1
GVALPLDSRDTSCLANVFALLVINRVGRIDIQDQINNLNTWRRDFNNNINVNLDFTQSFEPLRFVRYSIRNTSQFISNALILSNYRD
TAVGTPRETIRIPIPRALAIGTPAPSTYVGCVELPRLDGTWYAQLE
>p2g9_EDU49735.1
GVALPLDSRDTSCLANVFALLVINRVGRIDIQDQINNLNTWRRDFNNNINVNLDFTQSFEPLRFVRYSIRNTSQFISNALILSNYRD
TAVGTPRETIRIPIPRALAIGTPAPSTYVGCVELPRLDGTWYAQLE
>p2gb_RAQ98980.1
ILSDNPLPIDAAAEEAGMALVRRQVDTAYQTNWSWVLREETRTYWDLNNNVNVLIDAQTTEFDGRVIVQITNRSQRYGNAVILTR
YAADTNTGPIQDQIRIEVGLAVGRTAAFTRRCIRLPSLQGSWWWQLEN

Fig. S2 MAX-like effector candidate mature sequences in FASTA format (de Guillen *et al.*, 2015)

>M.BR29.EuGene_00004921
AKKEKDADTDPDTKMCDFVIKKSGKKITSGSVWPGATENFMVDGQWPVVSATQQCRITMSGLSDIDQYTITLCDKKEDETVSSD
GRNTTVISSHGEWC
>M.BR29.EuGene_00041131
NPHFCEVSVSYENANKQKITTATKYVVPPGSVDIAVGSQMYAVTVDETCKRTDSISIPGRFKIDTQGKKLEGNPTENYIRSVVLQL
NHRGLRMVSRPAP
>M.BR29.EuGene_00043011
CDDAHAVEDTNNNRRSCYYTIFHRAGREWRQIRQTITQITPNCVVNNIRGIPKFWVDDDCTPNYQKSEEVYIRVTAEGAKNYPEP
PGFPSGCSLRGRPGSKWKSWGELTPLKQPMRRG
>M.BR29.EuGene_00060181
GKSYKAQKICHFTILKMYKNTWTFVRKDSAPADQTTTIEGIKVFFSKDCIPQHDYRGLYIEYNGFHHDVSQS
>M.BR29.EuGene_00081821
GGGRKPEPWTAEQKANMIALSRSTLSRPRCNIIIYGLPSGRNQDRPVMGEADTALNSEVFITVRSGEKYRCTVKDDCDKPTCHE
LPPHLTYAGTDMHAIPLVTLRSLGVEIN
>M.BR29.EuGene_00082031
RRDDCWVSLFQYKANGEREAIYVDYSPPNSLVIFVHDGWRLNVQLDQHCRSLFPNDIRKQLVEGFGVHSYSKMNEVAGQKPLA
RSQ
>M.BR29.EuGene_00085071
IGCKFTVLDGNQKDQAEGCCIGPGTIKEGGLVIKCTDACGLSVTGGDSGYSVQSNGRC
>M.BR29.EuGene_00087671
KGCPYSIKRYGEEVKWDIIEAGTSHIRVVNGIGATIIAKKNCKLSLKGLPRFEYTISRGDSINRQS
>M.BR29.EuGene_00088411
QVNNGCVIAVIKSAKNLSDEIDLGEACIKEGTTGTVEFGNKSSVVVDLKSPCVPTVHEGSVLPAGTYLTPVAGCYMK
>M.BR29.EuGene_00091361
KNCEITILQGESRIGFVNVPSSGKVRENIRNWWYTISCDEDCNPKISGFTYTVEKAC
>M.BR29.EuGene_00091681
NTPPGSPSSNTISDKGDCYLTLDLGKYRYETWPKLREPFRFAGYVCKMGNTCADIMCKNFPQLRIWEFKSRKYPDGPWGDSYN
EGPVVQVRPWTQR
>M.BR29.EuGene_00095641

TRPSKGRLKRVVICEVFIRMSGGLDLPIGKDFVPAGETTKIHAYTCETDTMCGAECDTPSNYRYSSWVLERELRDWEKARLRAR
LALASSPESRERANMLVKLLC
>M.BR29.EuGene_00106461
AAPEFCAYFTGSNKSRRQVGVVRIGEIDTIIADGTELVVHAQDSRCQVILANGKPGPEWLSADPV
>M.BR29.EuGene_00107481
SCTYSVYDSIGKMVVEDKDLPTNRRDYYTIGKKKYTFYFKENCDFDYSKPPLPRCWHVSGIPKGLYDQVQGMEQPCPAQ
>M.BR29.EuGene_00112111
ASDPYFSPPSESQKCFYTIYRVESINGEKVLVREGVDHSYTGWDAEIKKINVHFDKECNPQYQSSQWVFFKGVRKERHIYPDR
>M.BR29.EuGene_00113041
KYGDCFIGLYEKGGRNPNHIAGTEADESGTFEVLHENYKIRVKIFKCVKQHVYDSTPSGTEIRVIPSVGYDPNKRQTAPRPAPRP
APRPAPRRTQNAAGHR
>M.BR29.EuGene_00118801
NPSDGPEAPEELCFTTIIRRRDGFKYAEKGPIGQKIAFLAGLNCTVQKGCKLACTPAHYGLKYKVSCRPA
>M.BR29.EuGene_00119491
SFDCYIERLQKEHMLSGMKAEPNKEAWIDGFEIWVYSNCRIHPTILPDGSIANGRFWSELDWFHTCEESESKSKSE
>M.BR29.EuGene_00119511
HKCVIKRVFSDGSSVKIPAQPAQSTTISKQAVWVYGNCQPFPVVLNDGSKLSGEIETPAVTPVESVDEDDCFELDISGQR
>M.BR29.EuGene_00121691
VGECHITIWEGKTPIGTEIAIEGGEYVTIAGYQCNAGAGCTAHCIGLPKNLKERGVYHGEM
>M.BR29.EuGene_00125811
VGPCVITIDGPYNPKYNFYPSHGRGVTGPHGQVVIGDFTCTVGATCNQLTCNAPQPEYSILGVDSSKWGLSDTESDSDSDSNSK
QAIMPHNRKQVAPVSHY
>M.BR29.EuGene_00126081
QGTGCNVEILNQNQVSVGSGCARINSVTNISDNQGRRWGVNANSSCGLSTTQTLPSGWSLTHNGFCNA
>M.TH16.EuGene_00000541
SRPYHGRIQDLRRPLLCDVYIEYPSGQVFDQALGHDIVPAGETAEISGFKCKTVRGTCVILPTCDTPPEYRVTSWKFEREMSDPE
RTRFIARFPNVRI
>M.TH16.EuGene_00027191
ELCTIDILFRGRRVTFRNIPSGSFQQTEVQGKRCIISVDDSCQPTQVGLDQNYWSVKLREEALGL
>M.TH16.EuGene_00027411
LKCLIQVWQFGPGMRLVSFKRVEAGEIGTISEDDVCGSVSVQTEEHTCQGTIVMYSLREGFYVTLDKDPRSIEKDQSKSQSSDK
HS
>M.TH16.EuGene_00034081
SRCVIFKAKRGLNQTPEQVFMKAPSDSFCIDGVLCRSSDTCYITCDRVDHYYKIAGGLDPTRKSKPREEKQSC
>M.TH16.EuGene_00040131
ARPNFCEYYIKTPEGWQLLGWVKIGGQDELTWKGDSIGVKAEDTNCKVVLVNGQRAPVWLTAVPSTH
>M.TH16.EuGene_00045871
VWECHVTVWEGNTPRGTEMLKEGGDDKTIAGYTCRAGAGCKAECDGMNPPFRVLGTPHSEWC
>M.TH16.EuGene_00079081
NGRGWCKVKVLYRHNRKEAQATYAEIDKETHWNLDGFPIVIKPDGDCIIKLVSGWVDHETYYFKGETHKVTTYSK
>M.TH16.EuGene_00079311
GKESYKVKKICHYSIQKLHGKTWYQVGTGQADAGKDTHIEGTKVFFSNQCLPQWNNRRLYIKLDSEEVVPV
>M.TH16.EuGene_00099371
DDVKKDCYYTVWTSNGNSWLRSIRDKTWSDHTLGIHGASVYFDEDCYPHYHSKEILVRKGCHEETTIPK
>M.TH16.EuGene_00101881
APATPAPSKPAAVLPQLCKVEMFDNNARFVGMARGNWDKDFVIKGHTIKCNTRCETKDVVLPKNWSVKGYMIKV
>M.TH16.EuGene_00106621
ADPKNCKFNVLNGSGINVANGCCDVTKKQVTVSPFTVSCTESCGLAIVSGGDPTFSLQNAGRC
>M.TH16.EuGene_00120731
MFGRRRDGRMPPWEWVTWKDFCRLTLKKGDEIQSQSKWVAKPGPIGILIQPGVYESFKTDKDCNPKIRRGKLPDGYTLHGQLK
YITPDLASMYNRVLDDTKWWPFDSQEAPQTSTTVEKSL
>M.TH16.EuGene_00124981
GLGCTYSLYNNIGRQLVSYAPVSPGGTIDYKIGRTTHKLKFADTCEFEKSVPRLPDYWKVFGHVEGQMMPVE
>M.TH16.EuGene_00127871
AFDCTIERRLGTQTKARARTSAKAMPEMIPAESGKHTVIAGTRVWVYGDCRTYPTHLPDGSTISGRPFQASDVEEDCIELDVHG
KPKQ
>M.TH16.EuGene_00134971
GKWRCNVKIYNDKRKYQGQASDDWGETLTIRGYTCYTDSYCKASCNGLPAGWTAEGTQLN
>M.TH16.EuGene_00135161
QGTGCSVEIINSNQVSVGSGCARINSVTNIGDNQGRRWGVLANSSCGLSTTQNLPSGWSLRQTGFCNA
>MGG_00821
ESDPHFCKVSVYYQDENTPRYQSTKKFAVPPAKVPILVGSRMHEVTVDKACKRTGWNGWFGLPRGYTIETEGWRLKGAPEEN
EILSLVLQLNEGVLRLSSGIATRS
>MGG_04384
VHECFTTIHAKTPFRSSYSPEYYKASLLQMRPGEDYYFKNPSPFEGCLVKFDEECTQLIWERLFDFPETTACPPADAKVDVPTRK
EVAPPGTKTKLN
>MGG_08482
GRGSYLTVKPSELDKHIAQLEEYLRNPPCKVTIYAPPPKKGRTQLRTEMGFASAGKNEDVPVKIASTGHTYQCRTDATCEEPVC
HGLPNGWTYDGVYMHTFTELSLEQLKAQKAAQTSRH
>MGG_08944
APTPVPGIFDIFKKPVPTSCKLALTNGNKREVDAMLLPPSGTTILSDTSGAGVFTAKVNSKCEFTSVSPALPTGFKIEGSVDKEGK
PPKSSTKPDTAAGTKPSTKPSTKTGSKTGTKTGTKAQA
>MGG_10120
NNPDSNDSDCHIWIKDNSDIVVGYSKAVTGQTAVVEISQGPHKKEQFKFATAADQTNKKDCTAGLAGERSFPAGWRIKTFKTEP
WVTLTRNYQPIAFKGYNRDPQTQSGSLKFFEIPYGEMKSGKDCNVAQTEKASSVKQPAIQERDEQQI

>MGG_14793
VCDFIIKQNGTQITSGNIWAGATANFLVSGKWAVVSATSECKLSLSGLPNNESYTILPTQDEETEGEDIEQADTPEAS
>MGG_14834
IRDCRLYYCWGPGSDNRRVVGTVDKGGSLKDYVNGETITVKAGKDCKAEIIGGKTCDMMPSFSWCLVNASHATSPTRGARELP
DWGSNWTTGTQPPLRDMSAQDSQTALTRVMYANCYKFPGVAKAMQILKSETAHDAAEAGVADFNKD
>MGG_15207
INEDCIVSLVPDGGSFARKTLRSSPGQQHDLQWGKYAFTIRVENGCAGKKTAGTIPPAHFVSVGRAASLKPTSILRDGDPRPPTA
VYPPRQPPRTEGRRTRYGSSGGAVTVGAR
>MGG_15459
GEGDYYFTPPPATEKCAYTIYRVASKNGLKALVREGDYMAYKNWDQEIHGVQVHFDGECNPKYKSDVYVFKKGVREEKHIWLS
PPPNKSSGVH
>MGG_16058
LLWGCNPTTVNVTFHSVPGGQELSSTELIIIFSFYLFLFPGGLLPWFILPPGLILIANWFLHDTSPWSTALPRLTASAATRLSHPWA
NEPLNHICSFTVRALAARKLC
>MGG_16113
IYECYVSLKINGIEHYKPEGRMEFPGATTVYDIGGYGTVVIVLGDTCEPIYKTELKLPQAFDFSSRIFKKRKQSEGQPSGREEVKH
QRIEAQEWKPGNV
>MGG_16175
ADCTLGCKYLENNRWVSVSKSANIGDTLYIMGHSTKIGRGCKPETTEWSDAEIYSW
>MGG_16619
GGKNGLKDCQVSLTATSEARRSFWDTQYADYNDDVEISAGRHKFVIHVEQDCGGSKVSGTIPPHHLVILSPMSGGGRRTIINSA
GEVRLAAT
>MGG_17132
LGCEVLISKKSGGDGPVKSSCIPKSGSKVIVINGKTVTVSADGSCKFSSKDLDPSLAMKFEGDCIGI
>MGG_17255
KCVIGLTFNGNWVPNNRFVISPGGKFSFIANYVIIPVRLDESCHWHQDLAENAKPVIIPSGYGIKSCDEGDFVNNQCQEPPKRTSI
GGTKVYAFS
>MGG_18019
MEDGCSASILKYDANGGQETYYGSALPANGSIAFQPTEFSTEVVISVDANCNPTNTDDVKKSVPKGWGLHVYKKVTAIAGES
>MGG_18060
KGCPYSVRRYDKEVKWDIVEAGKSHIRVIDGYPATISADQDCKLKIKGLARLKYTIAPGDSRPDRCKGNSIGTS

Fig. S3 Phenotypically-validated effector candidate mature sequences in FASTA format (PHI-BASE).

> Ave1
DLGTASYYNPPYLPTACGGSNPSQFPSGNLFVAVSDGLWDNGAACGRRYRIKCLSGARGSCKDGMIDVRVVDRAKTTVTKAA
HKATMILSQDSYDAIVNQWKGTRHKAVNIEFRQI
> Avr1-CO39
WKDCIIQRYKDGDVNNIYTANRNEEITIEEYKVFVNEACHPYPVILPDRSVLSGDFTSAYADDDESC
> AVR2
KKLPGCDKDPCKVKEKSGKYKLKIGAKCSATCDGKLTRGGTCENVQGNHLCCFGLCG
> AVR4
QPTNPPAKTPKKAPKTQPYNPCKPQEVIDTKCMGPKDCLYPNPDSCTTYIQCVPLDEVGNAKPVVKPCPKGLQWNDNVGKKW
CDYPNLSTCPVKTPQPKPKKGGVGGKKASVGHPGY
> AVR4E
DFSRDCPPGSGVGNQAEWSARGVDGTAIPRELDAHSLCDCFKPFLNVLGCSVTSVVTERAVFVKGHLNYCARMSESVAGISPC
KEWEIEVGGAHPERMANR
> Avr5
SYDALPINCRDTTNYCFNGNGRHEVCSYCNQAKEEPLKLGRRGGQRDCGVAGSQCNDVDHQQCDARCCSKIGSPTFYGVRC
PYPY
> AVR9
AALPVGLGVGLDYCNSSCTRAFDCLGQCGRCDFHKLQCVH
> AVRa1
RTWQCPSGDILYEEDVYSHTNMNSFEHFPFKTIEDEVYENTSLSRGVVYKVLDVHPSSSSATTFAVFTSNKRVAVVYQQGYRNN
RKFIEECT
> AVRA13
GDGYITLGMGSIHKNDIYRVAEHMWTIDAYSVPSNNHGSYPIFGEEINGSVTRIFPIVYNGDDWRSGDFYYSVESTEDLSYIKLRY
NGARYETCMVSSPE
> AvrL2-A
LPALSSKVELSAQKIKVQARVNQFVRENNRPPRRSELDVILSQENLKLKAGYLPPQSSTSHEDNWETLDKEIEEYRNGKSFKVED
LPKEEELVKYKADEVPPPRYDDYFIKTPK
> AvrL567-A
MEHVPAELTRVSEGYTRFYRSPTASVILSGLVKVKWDNEQMTMPLFKWIGGEQAEELHFCVHIAHSSGPKLNRARSLGTVNSN
MDQHWAQAQRNSGATRRTIEGFHLFENDIPNFPDYIKIKLVPKT
> AvrLm1
SPATKNNVNQPLDNISRRSEWKSVQISPVKEHSAKTADNTENNHNLEKRVFTSPHMKRTFTLALENTFYAMAWLIDFSFSDDGE
PHFSYKLQSFNHEDNPPKILADVVVPLITTSYNSANQYRGKANVELLCHLAKEYVHVYFSVEVFASGASFVIGKIIDYPTVYVNNQ
FRKVVKFDIAGAI
> AvrLm11
FEGRPCCQKWIEQCKLEGAVGCMLADYNYCVVPSSTCQEQCNGFFGRNGRSTHGRDTEYVYQIHNMYTANEKDPTC
> AvrLm4-7
CREASISGEIRYPQGTCPTKTEALNDCNKVTKGLIDFSQSHQRAWGIDMTAKVQCAPCITTDPWDVVLCTCKITAHRYREFVPKI
PYSSFSSAPGVIFGQETGLDHDPEWVVNMKARTRGCD
> AvrLm6
QPHLLCACESGRRDGVDDTRTLKVVKGTGGRFVFSSRYWTKAEGAPHEGNYAHAINGTITKKGTNIQAHDDGLIGGEEMNSLC
PEHSTCFSPNLKAKSTHSCGPDGKYGCVSAWLSVNWEGQIQ
> AvrM
HPMNSAKLAEEVKDGVNQPEFDRGFLRPFGAKMKFLKPDQVQKLSTDDLITYMAEKDKNVRDLAIKLRDAKQDSTKNGTPEIKQ
KYDKAYEKTKAAAEKLVSEESLTRDALLELTEEQYVEKAALFDKDVYRNNLQRQTYERLLRSETDVSYREVARTFIAREGEPALN
AKIERLALTLENNADTRSKLDYLAIAADFLKNQANLHADDPELNLYKAETKAREIKANRAMKEALEGADKLFERNKILKSPDMRYK
SAGFQAFIDKMMAWLSKIMTTKSYIKSLAKP
> AvrM14
AGNNDLKNVKAIAIVYRELSDGSQTVLLAKMKGLADEKGWMFVRGHVREDEEADPGVAAIRETQEETGFTGMVKKSGAPFTQP
GSKDTQRVITIHPHIVQVQEASKSKDTEDTVKRQFLWVHPSEVRSKLQRAEMIQAWDQLHSFF
> AvrP123
QYVVDPGFGEIECMCGQIARLTQRPFDVECEATPSCSCDYRGDCPGPAAEYVYRCPTCGPRSHVGCFGVHQGTCEEVHPGIA
RVQYQNSDSESE
> AvrPi9
LPAGGLPGSPGSAVQRCHCPPRGSHAHGSLAAREEAPEAEGDAKISARYTCPNCHKTGKGCDDGWCQVEKTHW
> Avr-Pia
APARSCVYYDGHLPATRVLLMYVRIGNTATITARGHEFEVEAKDQNCKVILTNGKQAPDWLAAEPY
> AvrPib
TQVTILKKGERITWVEVPKGESREFNIRGKYFTVSVSDDGTPSISGSKYTVE
> AVR-Pii
LPTPASLNGNTEVATISDVKLEARSDTTYHKCSKCGYGSDDSDAYFNHKCN
> AVR-Pik
ETGNKYIEKRAIDLSRERDPNFFDHPGIPVPECFWFMFKNNVRQDAGTCYSSWKMDMKVGPNWVHIKSDDNCNLSGDFPPGW
IVLGKKRPGF
> Avr-Pita
FTNIGTFSHPVYDYNPIPNHIHGDLKRRAYIERYSQCSDSQASEIRAALKSCAELASWGYHAVKSNNRLFKLIFKTDSTDIQNWVQ
NNFNEIYKECNRDADEISLTCHDKNVYTCVREEVHNLAYALINEKEIVICPPFFNNPVNSREITAGNQDTIILHEMVHIILKEWKDYG
CEWDGIHKLDSTESIRNPDSYAIFAQCARYKYC
> AvrPiz-t
SFVQCNHHLLYNGRHWGTIRKKAGWAVRFYEEKPGQPKRLVAICKNASPVHCNYLKCTNLAAGFSAGTSTDVLSSGTVGSIGN
DPQAQRQ

> AvrPm2
ESYWDCKGIPILFRTVHAAVELAFTSQPGSISGYPSICRTTPLRTGPDERRQFPLTDTGARWQGGGITYYVEATRDKRHCEVFGT
AGGVYKCTLVLRD
> AvrSr50
ARSLVKIDWSGSEYTILGANHYEEPNTGAAAQFPGTMTVDDGRSPYIVRKLRNSSGKRFYVFTGHPQQPIVWNPHEEIEIQFNRK
FLIAVLTEFEADSQVFNHFARRQHR
> BAS1
ADQGSNTFDQRYQGYPWRPANGPIREEKQENVGHRRGGAEYFTSGSPSIAAEDYSAKNAPSRFEQWKAQQKERAERKQDRG
LNGIRRVENYYP
> BAS107
IPTPINLPAALTSQLEPRAEGGASAGDANNIQPQSQEKSPFICVWCGKDKGGSSALIGHEIHNHQDDEMVAWTGRDRRLNKYET
DKEGYRYFKKYPGVRLPNRTWKGKGKRPE
> BAS162
GGGTYIPKDPALAHRAPEKFPWSGLLNNPFMTKPVIWYQIGQIKIWTDTRDVTYVRDGTKFDTYDQCLSHCLKYSVFNKSKARS
SGSQGQTSEYRGRRMHH
> BAS2
NVTPNDAGAKNVGTGNGQQFITGGCVNGTDCQSRCCAGNGENKGVCSNEVAANQNGKTGCGFEDPNKAQTVKEAKEQVKK
QGF
> BAS3
MPAETSPVPKPALPVFEELCPDAERQKCAESTDNLKRCLQINGASICVIDCGSQTTCRTQCKQQLKNEKANGFCTVGDNPCICN
LNGAANSAH
> BAS4
DSHQNLVCVRTYPDNNSAENLEATRCACDWLKKNGKCDDCTIWENRLCHSDAKSLDGNEFEDACVRKCPNLGATGSSIPPA
> Cgfl
HPATGHAHNIGRRGVDIEAFRLPQVGSYTNATEIETTPPIALLKRESYVETATELVKKLAPNSEFRLVGDHYVGTNGIGHVNFKQT
VHGLDIDNGDFNVNIGKDGKVFSYGNNFFKGEAPEASPLKKRDFKDPVSALKGAKDVLQLPVEAASASAEPKEGTEVYSIKGTS
GTVSDPEARLVYFVKADGSLALSWRVETDITENWLLTYVDAETGTDVHGVVDYVSDLANYRVYPWGVNDPTEGDRVLVTDPW
DISASPLTWQSDGTTNYTTTRGNNGIAQSNPSGGTAYLNNYRPTSAARNFDYQYTTSLTTPSSYIDASIAQLYYTANHYHDLLYT
LGFTESAGNFQINNNGKGGVGNDFVVLFAQDGSGTNNANFLTPPDGSNGRMRMYLWTMSTPRRDCSFEAGVVIHEYTHGLST
RLTGGPANSNCLNALESGGMGEGWGDFFATAIRLKPTDTRATDYSMGAWVYNNPAGIRSVLYSTSMTTTPNTYSTINGVSSVH
RIGETWATTLYEVLWNLIDKHGKNDGPRPEFDSNGVPTDGKYLTLKLVLDGMALQPCSPNFIQARDAILDADKALTGGDNLCEL
WTAFAKRGLGSNAVYSSSNRRDGFNVPAGVC
> Cmu1
AAVSGKSEAAEIEAGDRLDALRDQLQRYETPIIQTILARSALGGRAPSEQDEVRAALSRNAFEPSEVISEWLQTESGARFRSTRPL
PPAVEFITPVVLSRDTVLDKPVVGKGIFPIGRRPQDPTNMDEFLDTSLLSLNQSSTVDLASAVSLDVSLLHLVSARVLLGYPIALAK
FDWLHDNFCHILTNTTLSKSQKLANIIQQLTDHKQEVNVLSRVEQKSKSLSHLFRNDIPYPPHTQDRILRLFQAYLIPITTQIEAAAIL
DHANKCT
> ECP1
TVQGGAPVDDLKFAKKFNQNCQQISGGPNGAICPDGDLYWCKDGRAIFCQTCQTGCTADENGKVGYCNEGPTNPKCL
> ECP2
RNAGNSPGSNRCDASTFNNGQDFDIPQAPVNDCRQMVENINRDSQFSVSHSWARPFGGYGDCAFNVRVIAGWRNGLVGGAD
AVDLLTDSVKNFGEANKVSSKGTYNQIVSAEGEVTCDSVDRGGQVRVQWIVASSSYNPSNDD
> ECP4
DPSFRFSEYGNGDTTCSPDNLISPSVSIDDGFGGCKQFPANTNSVQYALLEGSRAGCIVKLFTNNACNESADSEHVPAVGETKC
QPPIGGGWQSVQVTCP
> ECP5
RGDNKPGQFNYICEDIPCDEEGVDSWILQACIGIGGTELTAHGGIYDTTGELVGRTAICLCARGQTKAHDYNISGDYPPGVAHLTF
DVRAPYWCQSSG
> ECP6
FVLPRTDDPDCETKATDCGSTSNIKYTVVKGDTLTSIAKKFKSGICNIVSVNKLANPNLIELGATLIIPENCSNPDNKSCVSTPAEPT
ETCVPGLPGSYTIVSGDTLTNISQDFNITLDSLIAANTQIENPDAIDVGQIITVPVCPSSQCEAVGTYNIVAGDLFVDLAATYHTTIGQ
IKALNNNVNPSKLKVGQQIILPQDCKNVTTAVA
> eff1-1
WPYPSGNHPDNADGARRGEQSPLPGFSVAHPSSPQNEFDPDLLRSLLAKIESEASSHPHVAFQGDGHYYPNVNQPSSLYQAS
QHGFGSLSRDKGQVGSSQARESFSDTQGLSSSAEPAFKYRKTVQRVADALRQFQANNRLEGAQALRDIIQRHPQLDTTSGSQS
QGFRNNFGAAPSVPSRESQGGTIHHGPGDTKVVDIPSDRIEAQTTAEESHESQPLAGHGDGRYRSLPASRADELRKIPTTMLAL
PPPSRTRYIYDQVDDPAIRDNINNQVFAGKLVWIDRARIPASRIVSSRKQMFRPHRVLPMTNFPEIHLSDGKGSIKDVRFTFHGGG
SYRLMSWPEGYNLVEGQNYLAFWGIPEGRQTGRPMLMQNYGYAFLPPQHRLEVNEHLWALKKEIADKASEATWMHA
> FGL1
SPLSVEEYAKALDERAVSVSTTDFGNFKFYIQHGAAAYCNSEAPAGAKVTCSGNGCPTVQSNGATIVASFTGSKTGIGGYVATD
PTRKEIVVSFRGSINIRNWLTNLDFDQDDCSLTSGCGVHSGFQNAWNEISAAATAAVAKARKANPSFKVVSVGHSLGGAVATLA
GANLRVGGTPLDIYTYGSPRVGNTQLAAFVSNQAGGEFRVTNAKDPVPRLPPLIFGYRHTSPEYWLSGSGGDKIDYTINDVKVC
EGAANLQCNGGTLGLDIDAHLHYFQATDACSAGGISWRRYRSAKRESISERATMTDAELEKKLNSYVEMDKEYIKTHARPLIIV
> Iug9
LTSGLMRRESGEVSTNYIFTDDPAEGGKTTQKKKRESGEVSTNYIFTDDPAEGGKATQ
> lug6
NTVAPADVAAVDAPRAPLLVRRNCEGKNTQAECERFKWLTGCTWLRGSKYCSST
> MC69
WVVITPITKEMVVDRSPDDCFFGVVTPQGCGPLRGPAK
> MISSP7
SPVPGEVGLVERGPIPNAVFRRVPEPNFFKDLLRALGQASQGGDLHR
> MoCDIP1
QCGAGNPDARVTGSGNNFQAVRGSNTVYSGSDYRAAIQAALDSIGSGQRVAVIASGSIGANTISISSGKTFEGCGTINVGNRNG
RGAIESLNTQGVKIPYLTMTGNPYFGLRFYGTRDLTLGQITMNLSGGLGIRFDRDNPDNFNVRMGTITVTGAGSHAVETWNIDGL

VIDRVIARNVGESGLLVQKTRNAQIGIVDGNNVGTGTGYATLRFANNNGQNPNGNYNTNIWVDQVISRGGGRGVFCVSQSGAA
VIRTVDLAKNGNNAILIENCYNLSIRGGTVNGGGEVRVAARSEFPNNRDLWITLRVDNTSVRESPCGTNVNWSLTGNGQRALC
> MoCDIP2
QDCISVALSAIPSCAQPCFLNGAPTIGCSGTDFKCQCQQQAKMFAAVESCVQKSCPESEFQKTIDGSDKVCTCASGGPASNNA
GGAGNTVNPSSFIPGPTSTASPTTTVAAPTGTPSGRPSAVPTAAANMAAVECSIVVGAVGGALWVALGL
> MoCDIP3
FRCAIGSAGSCTFRKMPTEELTRCAITCNVRGLFAECSCPLNFPNRAEWIDTMRNECITQTQFSFRSQCQNADPNRVIRIKRPAE
LDSVSGLEE
> MoCDIP4
HYIFSIVFVNGVQKGGDYTYIRKNSNTYMPSFTNDIVNSPDLRCNTGARANSGTQTLTVKAGDKIGFKLWYNEFIEHPGPGFVYM
SKAPGSVASYDGSGDWFKAYETGLCGGQPNSDGAWCTWQKDRIEFTIPPRTPPGEYLVRVEHLAIHESHVGKGQFYMECAQF
KVEGPGGGSPGPLVKIPGLYKASDPGIAYNKWTNNPARYVMPGPAVWADNGSSNPSNPPSNPPSNGGGGGGGSGSGAPLYG
QCGGEGWTGPKTCAQGTCKVSNQYYSQCL
> MoHEG13
HPLDNGGLEPRQQKICSPNGHTDGDLTNPNSLCTLCNSGCQRVAGQPACCS
> Msp1
VSVSYDTGYDDGSRSLTAVSCSDGANGLITKYGWQTQGQIRNFPYIGGVDAVGGWNSPSCGTCWQLTYNGKSINVLAIDHASG
FNIGLAAMNDLTNGQAGSLGRIEAQSQQVGLNACGL
> NEP1
APIEESTIQARAVVPHDSINPWGENVPGNALGNTLKRFEPYLHIAHGCQPYSAVDGNGNTSGGLQDTGNVSAGCRDQSKGQTY
VRGGWSGGRYGIMYAWYFPKDQPAAGNVVGGHRHDWEYVVAWVNNPEVANPTLIGAGASGHGSIKKTTNPQRQGDRLKVEY
YVSFPTNHELQFTNTLGRDLPMMWYDFLPAVSKTALQNTNFGKANCPFNDANFNNNLAKARI
> NIP1
TPDRCRYTLCCDGALKAVSACLHESESCLVPGDCCRGKSRLTLCSYGEGGNGFQCPTGYRQC
> NIP2
YYVVVCVPRDGAEIGDVEWAIQNRRHDLALGGKGFWRGHSTSCHRNANAVVDVVALCRSDPYIGAHPTVLKYGASVLCQASG
APDWPTCTVNC
> NIP3
ACGDVYTIKGNCTIRDYKCKYPRVPSCNSKGACPYLHLFDVPIFCDQDKDCVTSYKCNKNYKRSPLNLGPFYEDGDLVAKEEGE
GGEGDSELVEEDDE
> Pep1
DAAGAVPLPNFKVDPQPLASTFYWFSSVEVGVCYNPQARVGSIKGALHCTHQENYDRDNNSYTLPQTCVALKPLGKAFSSNVR
DSCTNAKGIFNVIVPASSNALGSQAYDAVQAKGGTGGTGTDDDTSAPDSNDQEKKGGGLLGGIGSMFGM
> PevD1
APASPGSTGAPPDPNMYENIDIADFNVRKGEDGTIKYVNFKLSGDDADGLLCEAQNPGLPSNVITCGESKYRFALSSGKQYEFAL
SLYHELGLAVGFYGTGEIFTHCRAGGLGDFICQQQNPTTIVIDSLPDAPAEA
> PGTG_08638
RSIPTVSDPLEPIVRLVTTPHEVLGAAPDLKETEKAAIQQSPYLYSHQIAAGLAETTDSLHYLSSEPQTAFSSLMAGSLLGYYRTIM
QDLPKETKESRLQIYRNSFYSRTRDVANHMGPEVWNTHATLAKKSLENGLQDVVLEFQKSQMTPAGDEKILNRIEILAQKFKNSK
KHQEIMEEISKAVAHMKTHLPYNVNLGEMKYLSAEEVESALKGTNIGGHPDAIPVNYFLVHHEPATHQISPAKTPITNSRQAQLPP
SQPKVATAPTAAAAA
> PpEC23
ASTSEPSGQTLDCSHYTGANTKEATCNEFPGRICHGGCTGAVVASNCTLNPGEEPKDQTYTIAFGKSSATISICRNEKGSYSCT
GPIKGSAKCSVCVDPPTGSDQSPTTPAPAPGNNGSTSKEGQTLQCTHFTGANTQSATCNEVPGRVCNKGCTSSVVATKCTLNP
GDQESQQNCTQAFGKSSAAISICINDKGSFSCTGSVSGNATCSGCTDQSSSGSSEYPGSSGTTKPPTDPTGGENKDQDKKAEA
TSLQFAMSSFCLALSLMIGVAVL
> PST_Pec6
SGASTPKKCKKTIMHEKDKCWTIGCTEEVSTAKWRLQCDDKNCAHPVCSGDWTPTKAICFVCLGNR
> PstSCR1
FKCPGLHGTPSQTHGYCTRSITDEERKAKKIGKEFTMWKEEIKTVDGKFSCDKVDLNGSVATDSFCCDVAGRIGEVEKSKQAM
WTNNCSKAS
> Pw11
GGRKWTNKVIYNDKGEREGSISIRKGAEGDFNCGPGYPGGPDRMVRVHEDNGNIRGMPPGYRLGPDDKEDKRDNQYYSRNG
YHVGDGPAEYQNHGGGQWGDGYYGPPGEITNQHGKRQGDQGCHIM
> RTP1
QLVGSDVVLVRTSHQSMGKASVSYCREMTRQHHKRELDQDANPGHRRHKSEPEGVKPSNHTSAPTPSSPPLTTVDLTPAKLN
TACYPGTFQAPLLEDCEVVIRAQLYNSTGSLQVSPGDYVFVSYGTCATVFQNPQYSKYSLQYNWAELGYVGGKLAGRCLLPED
HSMGGTAVFDTYLGRTYPDVIISLQRFDDRDFIIPE
> See1
HPLQSFRSSSAIGKQKHKIKSRQFEEEIAQGAEDSIELFEFPRVHDSSEQIHERTEQQNITTKNIILAINKNSRKHGGLHRLPAQVQ
GEGEFTYDRQRNAVGSYRYGDSHGNSREAEYSVADHQSASGEYKFGPTT
> SIX1
QEAAVREPQIFFNLTYTEYLDKVAASHGSPPDKSDLPWNDTMGSFPGNETDDGVQTETGSSLSRRGHIVNLRKREPFGEESRN
DRVTQDMLQALHDLCVERFGTGYRAVSGLCYTDRRATRKIECNKPSVRERDRSVTRACPKGQECTTFNAYNFRNRHHQVTFP
VCGPRIEVKDRHDIGIHTEWQGTWYPESPKSPGTYDYFAQMAGTLNGYFGYDGVYSDGYKTSSHGYGHSWSCINCPRGKVTIT
NTYRATWAFGYTSPH
> SIX2
NPAGDSLPDDAHLPDRRLSPSEVQALKKAQIYPPGYIHKRVTFGEGKDAVEVPIVEADVEMLLNNEKGVKARSLAPRGSCFSFP
TPARGSCMIDYCWRDDNGVIYSRGITITGSNGASNPTSMRSNDPANLSLNSVFNDGYNGWFPHGHACSNSDTQIYTNHRLLQG
VNGVAYVDHVRCENCNFRNVNCLSDVLKNNLIAYSNGVASQSRCT
> SIX3
LPVEDADSSVGQLQGRGNPYCVFPGRRTSSTSFTTSFSTEPLGYARMLHRDPPYERAGNSGLNHRIYERSRVGGLRTVIDVAP
PDGHQAIANYEIEVRRIPVATPNAAGDCFHTARLSTGSRGPATISWDADASYTYYLTISED
> SIX4

LPKGEEGDIIGTFNFSSSDSQPLKIHWVDTPDSSGSNLVKRSAHTESVCVHAGTATGADLHWLNAICTGKSTYTVNCAPAGNKN
AGSTHTGTCPAGQDCFQLEQVGNFWGDREPDATCSPSNTVFDAVDDKEATHVNGKVVTRAGKPGIGRKLIRLKAQVYRRDGH
YGQTSRMGFFRNGKEVYHIDNVASMEPTWNFDPSSDQSFSFFFTPGPNAFRIQGTLNLA
> SIX5
RDHQYCACQSGSGDSIDIDATTQLQNDNSKSYLWAQTSPAYWFADRHKPGPRFAGIYLKAANGKIDGDTFYNLCINNGGADST
CFDCSKSHQVRNVIYCDAA
> SIX6
GPLAQTESESADVAEHTINYIDIAPEEFEPPKANLSSLVSRDTLPVSTCPAGQKYDRSVCYKADKIRSFCVANPRSNREKITDTPC
QPREICVQRNLSNGKSFAKCIPIVDLVEWKTSANGNKEGCTTTSVNPAGYHHLGTIVYDINKNPIEVDKISYFGEPGNVNEGIGGS
TSYFSSDNFQFSKSRYMKSRDMRTFMT
> SIX7
IPMLDLFPRQGQCFSTTGSTPPRPPPAAREVTFDITQNVNTFTSAASTPWTEGVGLSNIRYQWRAYYSTRQRTTFVEVRVFGTA
EAQVVLLPDAPGTSRYRAIDSNVFRPNEEVTGGGLAGWGQVTTVCLQTWGRRGDITYRLRIQSKPKHSYPLEVIPERQYANRLM
NARCYAKTHQMIYKPRGLADFRFHRLTQSVVNL
> SIX8
TPIDKSLDQAATIEETVHQPHSHDERALVERDTSGILLACITGAGSAFQAYAGCYLTAFRNDPRTLTLRMDKTRGERISNVLVILSG
GALSHAVEEVVQIAPGAVRNLATLGASTVQFLHNFR
> SPD10
QCFDDLDCLFSGIENAKCAKATNTDLLGYETPPPCLLFTPKILGISADLFHNQSASRTCKGPA
> SPD2
QSCRQLSADFCQCGTYCYRTRDGGCNPAGTFYGLCPLKRETTFEA
> SPD4
LYTHHLTVDSHCPSLRSAVASRPGRQEVLEHNPCRKSDALVVITKNDESVPP
> SPD7
ATDLTETDTIAGADTRLVPGCLVKLYRPPKYIGCFDRSILVGAKCIPVQTTWKIKGHDISVWNGCSTVKGINLPKGWFVKGESAQC
KVWHRKQ
> SPD9
MPAPKTQPFQCPDGFSVVKNACFPDLGPDGDHS
> SsSSVP1
APLEPRQSIWGKPTDVSVQYCYNDFPCINAREAWQSCQVSSLPGSYSQITDPEARNTVVAQCLCDNKIVNETASCISCLSYASG
NFLVLDTVEQHAQDMCDKKTTLSQYENYVYQFGGLLQFPLILPSTWLSGTKVNLPDQILIKD
> Tin2
TGGFDYENLASSSSSSGHHDYRLESSPMGRFQTKDYANLLNSRLKDMGWGSEPLAKGLLVPREDLESFVSSVQDVLKTESGQ
KGLLYLGNTPDSRKVHAVLLKGAQSRSPNIAIISTPKVWRSLNQKIQLHTFAQIDNSNLFDLHQLLQHDFNYSQLDSLARNALPKT
PQSEFDNLLPRFPL
> Tox1
NEGILTFEGLGLARRQTICHTPGGSGCRASISGDQCCCTSCTPEDCSDLCKNGKQAAHEAQNQKKCAKCCNAGGESHELCCSI
ASAGIDCNPCPAGLRMC
> Tox3
LEPRGPGDIQLTREEHEAIFNGSPSDWTEDPNFKPDVPEQQRPATANDLSKRYIKANDINFGTRSVHDCRERTGIQRDVKVRADI
PFETDDGPNQVLRVTWSNALNVDRFDPLPIVTVPGNAASTTITAIHDFCLMNPTTSPPTRCLYQLRQPFTLGFDRTRMHNNIYLT
PPNPQRPTMHEVCIRADECPAGRVFLECSTRTYGAIPRGE
> ToxA
APTPEADPGYEIVKLFEAANSSELDARGLSLDWTLKPRGLLQERQGSCMSITINPSRPSVNNIGQVDIDSVILGRPGAIGSWELNN
FVTIGLNRVNANTVRVNINNTGRTNRLIITQWDNTLTRGDVYELFGDYALIQGRGSFCLNIRSDSGRENWRMQLEN
> ToxB
NCVANILNINEAVIATGCVPAGGELRIFVGSSHSYLIKATSSCGLSLTNQVFINGESVQSGGRC
> UhAvr1
PGDKASSSAAPAQQHQPSFKLEIAENPNVDPFLEKISKLGNSHDLYPHVALMRTTLYGKDKLTTNLGAYPDFRRFIYLGNSPGVP
EMYFAVPLHLNPHGVDRNLAWSLIYAHSDQPKTLVHHGFVSASGGHLVLDKVKKTNYPSSRSFEIGDVLTLREILDIELPALRFAG
> VdSCP7
APAMRTSMDAPMMEMANSRPMDMDMGSSTPAMRNTMNMKDASSSMGMAKRERVEMGNMAPTMKKTKGMKMGSMAPAMK
TGMATRSTMDMDMANRNSATKGMEKNKDVDAIVAAMLMEMAHRRPVAGMVRRQSEEAFQGVVEECKTKLASGEVTSLDNCV
LDTLGINRRQVTGDQDQLAQITQECTEKTPNGKSTRIQGVHY
> Zt6
APVAEPAELEIRQQATYCGNQYYSASQVSAAVNKGYNYYANGQQVGSGNYPHQYNNREGFSFAVSGPYQEFPILASGSTYSG
GSPGPDRVVFNTRGQWGGTITHTGASNNNFVGCSGTS

Fig. S4 RaptorX models generated from the benchmarking study superimposed onto their respective templates, sorted by alphabetical order as per Table 1. RaptorX models are shown in rainbow colours and the template structures are shown in grey.

(A) Avr1Co39 (2myvA).
RaptorX score 60, RMSD 0.19Å,
TM-score 0.84511.

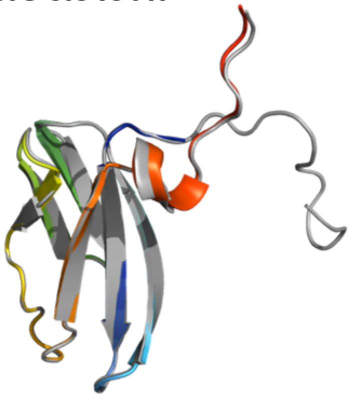

(B) Avr4 (6bn0A).
RaptorX score 64, RMSD 0.17Å,
TM-score 0.99718.

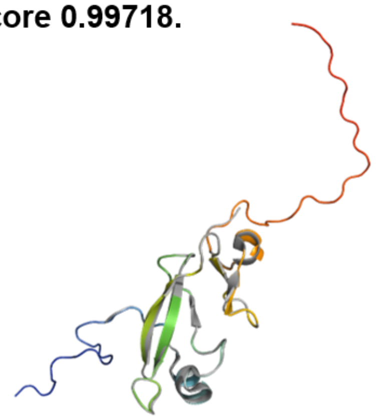

(C) AvrL567A (2opcA).
RaptorX score 100, RMSD 0.12Å,
TM-score 0.99908.

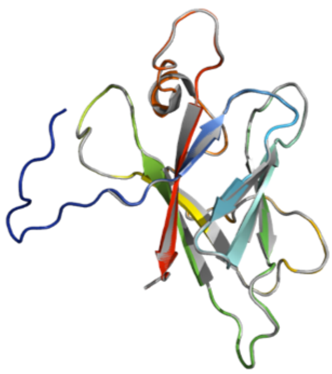

(D) AvrLm4-7 (4fprA).
RaptorX score 98, RMSD 0.37Å,
TM-score 0.93397.

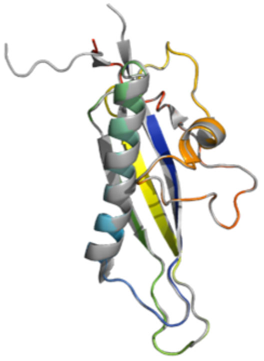

(E) AvrM (4bjmA).
RaptorX score 243, RMSD 0.39Å,
TM-score 0.99636.

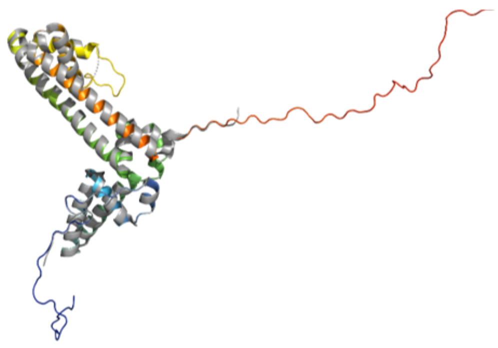

(F) AvrP123 (5vjjA).
RaptorX score 65, RMSD 0.89Å,
TM-score 0.94371.

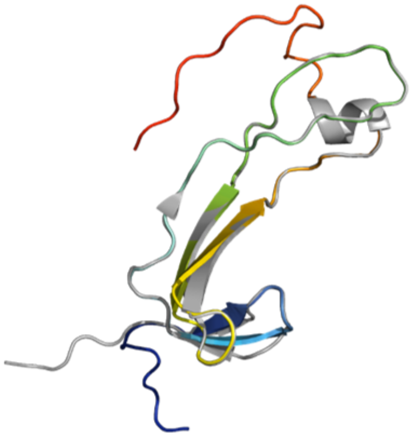

(G) AvrPia (5jhjA).
RaptorX score 62, RMSD 0.16Å,
TM-score 0.83321.

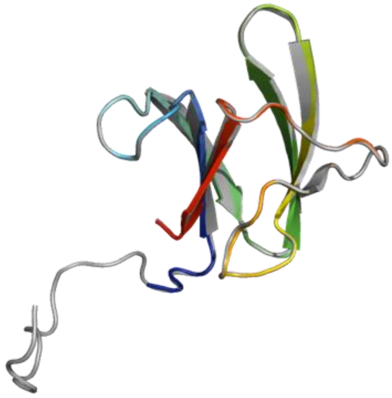

(H) AvrPik (5a6wC).
RaptorX score 76, RMSD 0.13Å,
TM-score 0.99843.

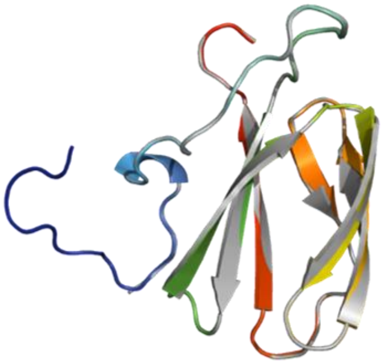

(I) AvrPizt (2lw6A).
RaptorX score 66, RMSD 0.14Å,
TM-score 0.99805.

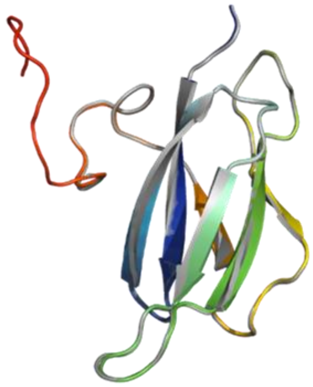

(J) Cmu1 (6fpgC).
RaptorX score 234, RMSD 0.07Å,
TM-score 0.99987.

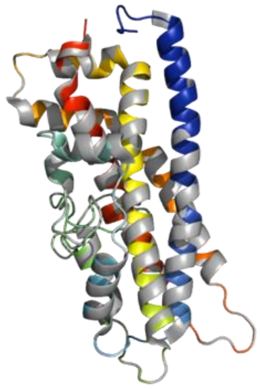

(K) Ecp6 (4b8vA).
RaptorX score 158, RMSD 0.10Å,
TM-score 0.99965.

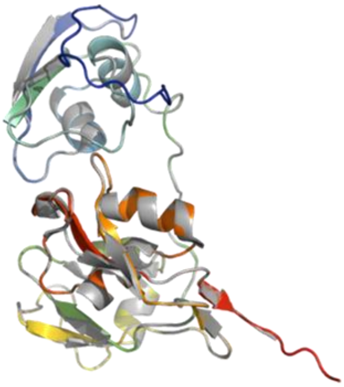

(L) FGL1 (3ngmA).
RaptorX score 325, RMSD 0.65Å,
TM-score 0.99313.

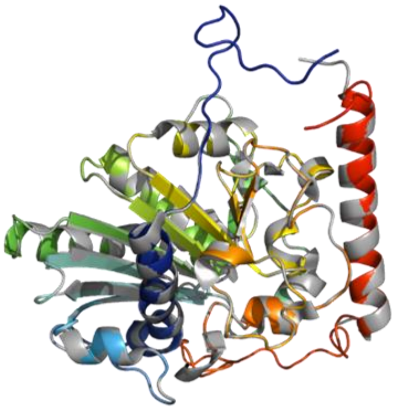

(M) NEP1 (3gnuP).
RaptorX score 204, RMSD 0.26Å,
TM-score 0.98383.

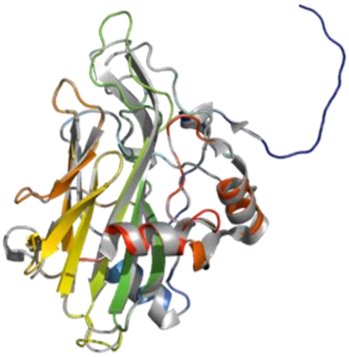

(N) NIP1 (1kg1A).
RaptorX score 53, RMSD 0.242Å,
TM-score 0.99162.

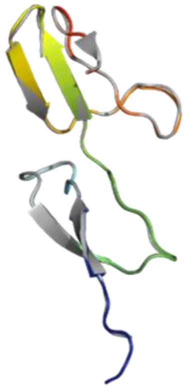

(O) PevD1 (5xmzA).
RaptorX score 125, RMSD 0.14Å,
TM-score 0.99891.

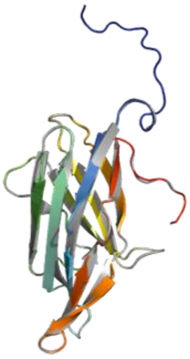

(P) Six3 (5od4A).
RaptorX score 96, RMSD 0.12Å,
TM-score 0.99916.

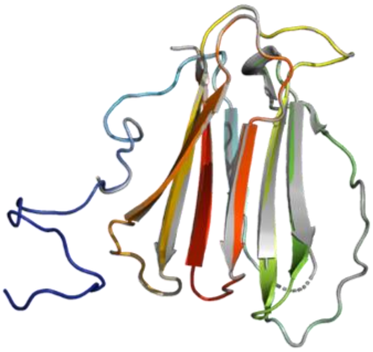

(Q) ToxA (1zldA).
RaptorX score 70, RMSD 0.13Å,
TM-score 0.98892.

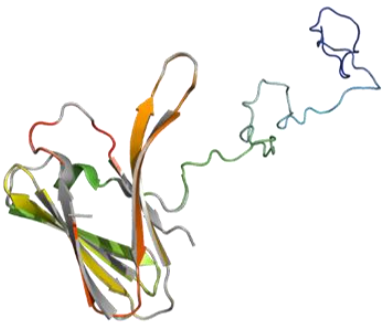

(R) ToxB (2mm0A).
RaptorX score 60, RMSD 0.16Å,
TM-score 0.99655.

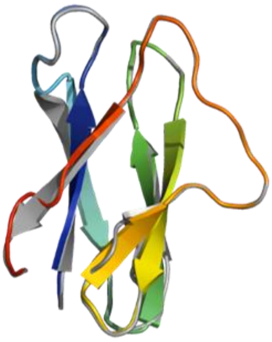

Fig. S5 RaptorX models of different ToxA-like effector candidates sorted by alphabetical order as per Table 2.

(1) p05c_mRNA16607

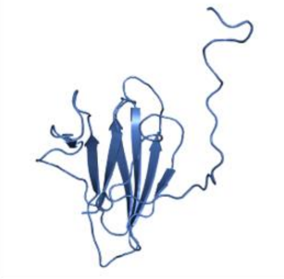

(2) p05d_mRNA9122

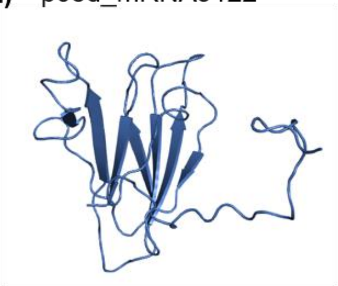

(3) p05e_mRNA13670

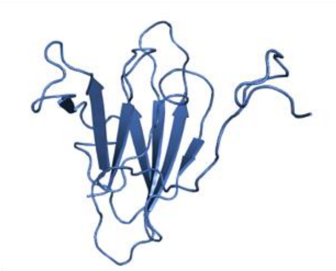

(4) p05g_mRNA17320

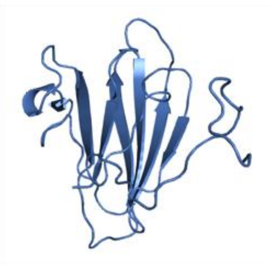

(5) p05k_mRNA3392

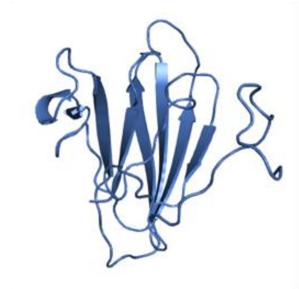

(6) p05m_mRNA12409

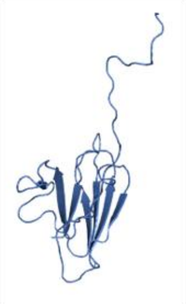

(7) p05n_mRNA11205

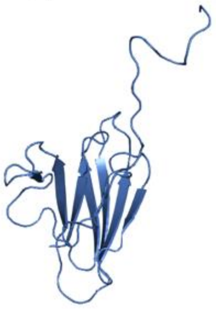

(8) p09v_mRNA10419

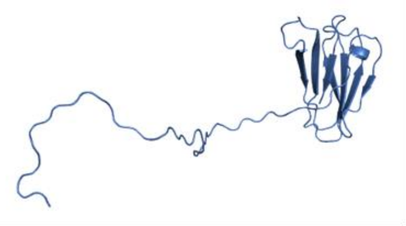

(9) p09v_mRNA9195

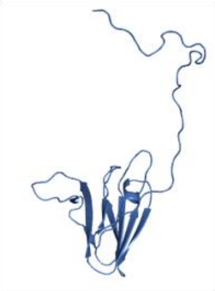

(10) p0dd_mRNA2255

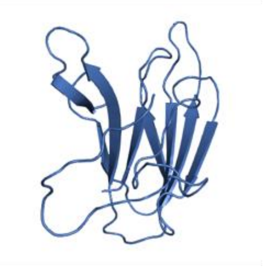

(11) p0de_mRNA10272

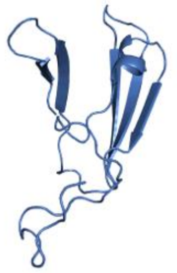

(12) p1ap_mRNA3793

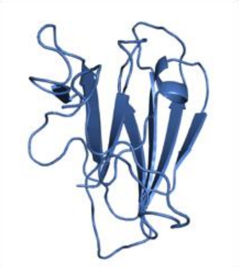

(13) p1b1_EXF72942.1

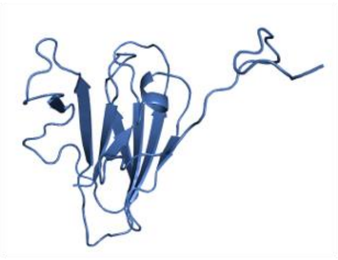

(14) p1bd_mRNA10016

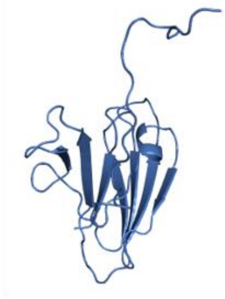

(15) p1bd_mRNA1147

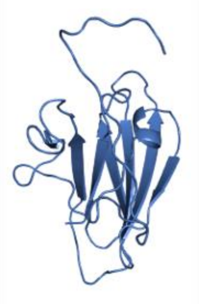

(16) p1bi_OBR06575.1

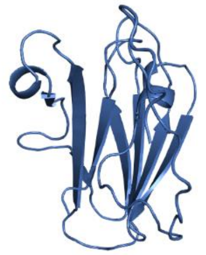

(17) p1bo_mRNA4951

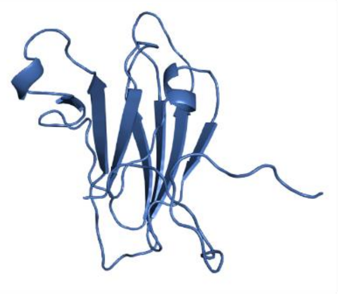

(18) p22r_EXK24251.1

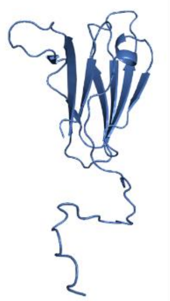

(19) p2fk_EMD96331.1

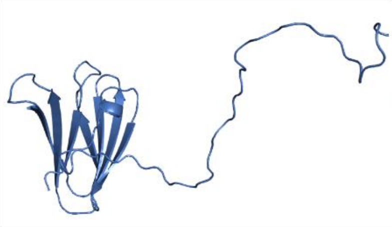

(20) p2fl_ENH98532 .1

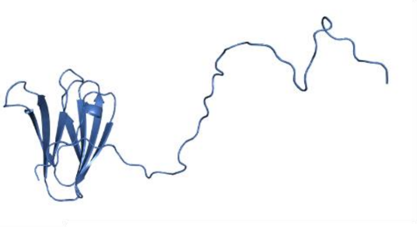

(21) p2fn_EUC44184.1

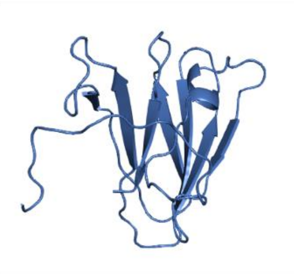

(22) p2fq_EUC36307.1

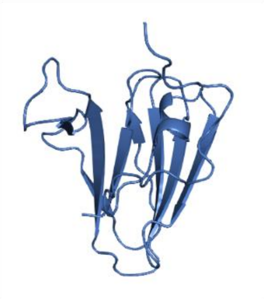

(23) p2g0_EFQ93895.1

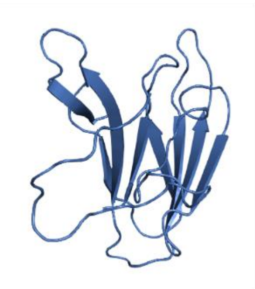

(24) p2g1_PZD05769.1

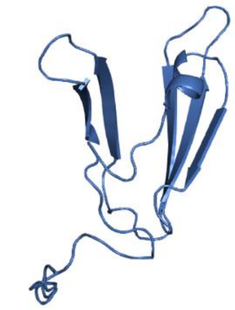

(25) p2g2_PZC93680.1

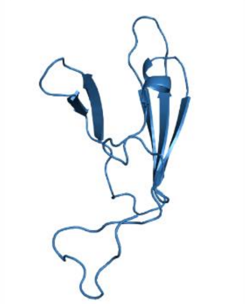

(26) p2g3_PZD24241.1

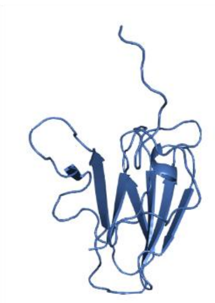

(27) p2g4_PZD32416.1

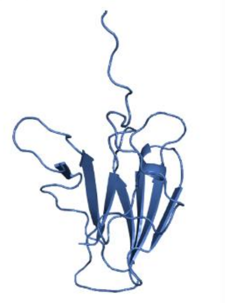

(28) p2g5_PZD46046.1

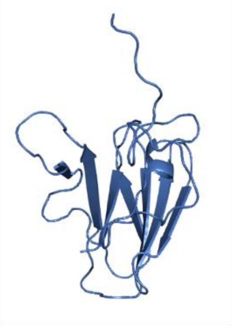

(29) p2g6_PWO08528.1

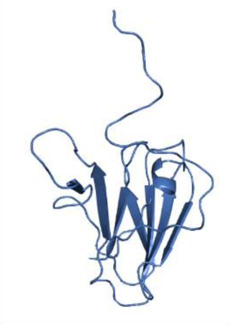

(30) p2g7_PZD04407.1

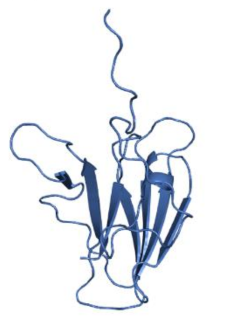

(31) p2g8_PWO20795.1

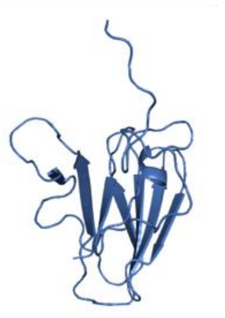

(32) p2g9_EDU49735.1

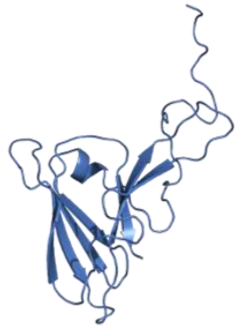

(33) p2gb_RAQ98980.1

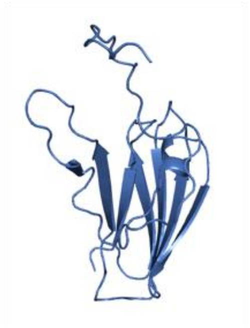

Fig. S6 RaptorX models of different MAX-like effector candidates sorted by alphabetical order as per Table 3.

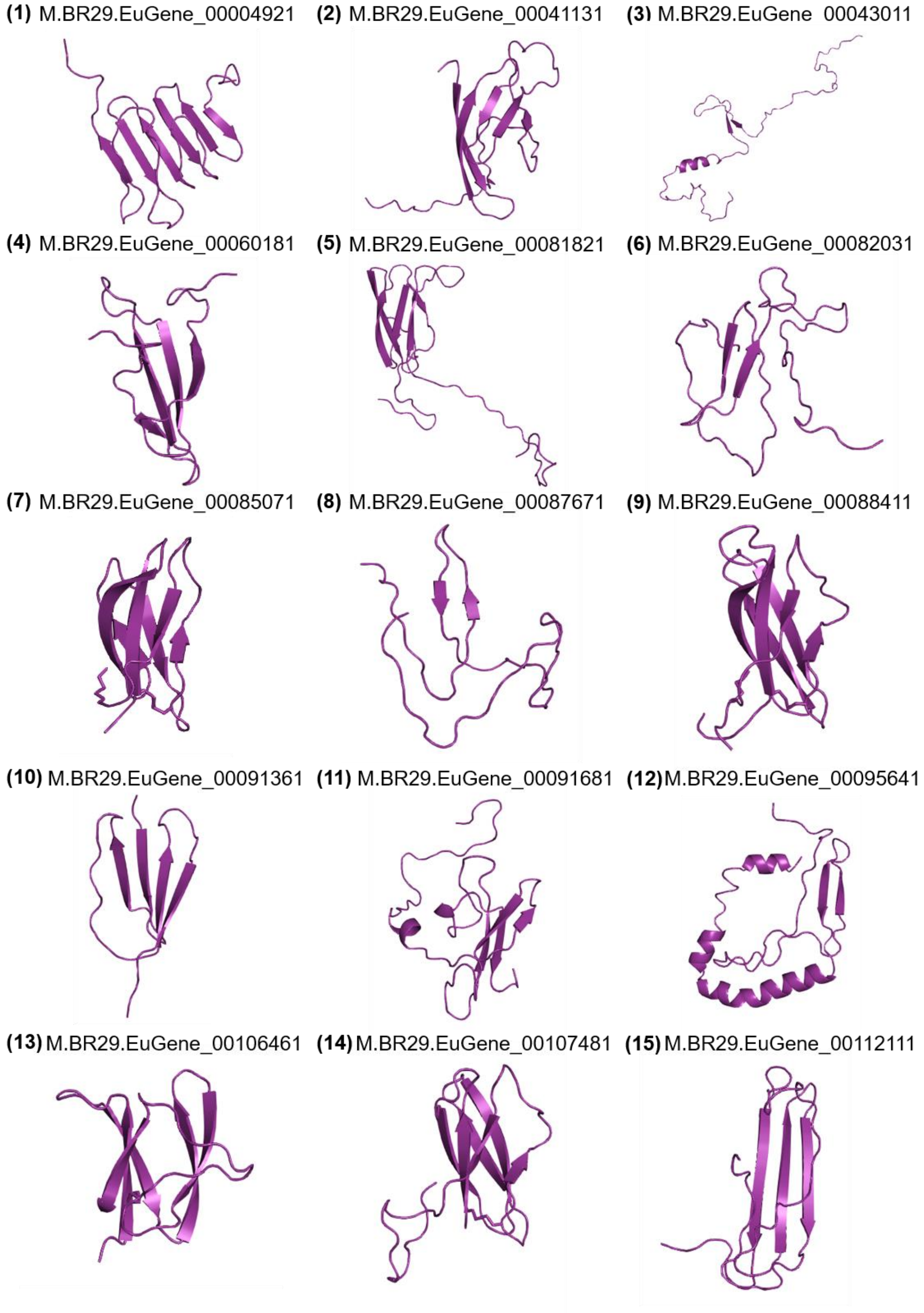

(16) M.BR29.EuGene_00113041

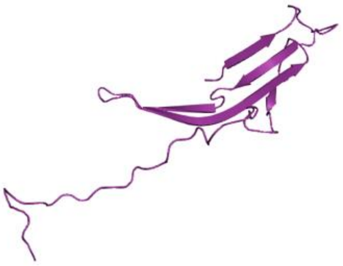

(17) M.BR29.EuGene_00118801

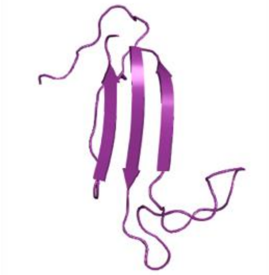

(18) M.BR29.EuGene_00119491

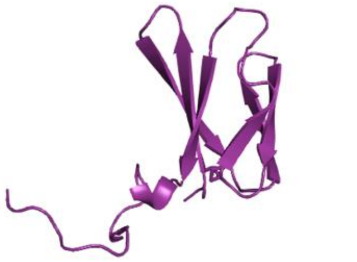

(19) M.BR29.EuGene_00119511

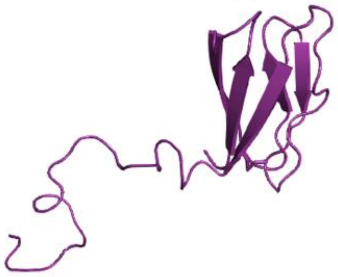

(20) M.BR29.EuGene_00121691

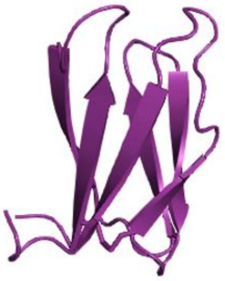

(21) M.BR29.EuGene_00125811

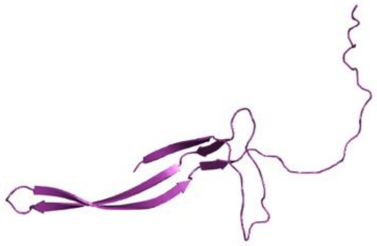

(22) M.BR29.EuGene_00126081

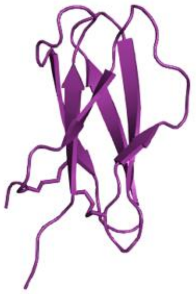

(23) M.TH16.EuGene_00000541

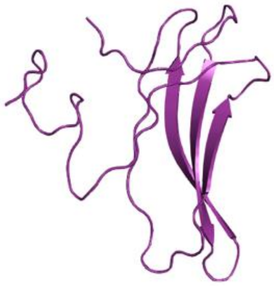

(24) M.TH16.EuGene_00027191

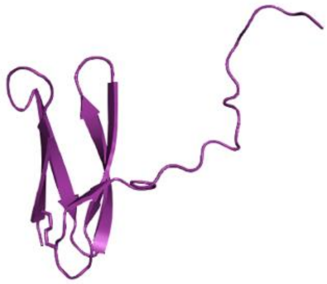

(25) M.TH16.EuGene_00027411

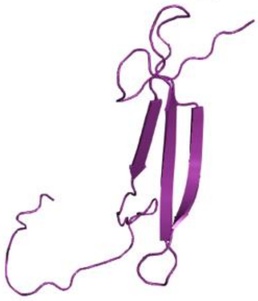

(26) M.TH16.EuGene_00034081

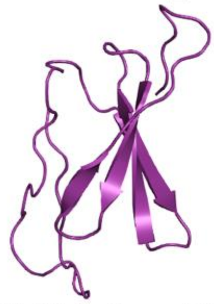

(27) M.TH16.EuGene_00040131

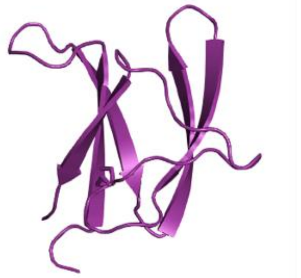

(28) M.TH16.EuGene_00045871

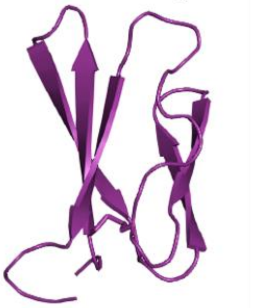

(29) M.TH16.EuGene_00079081

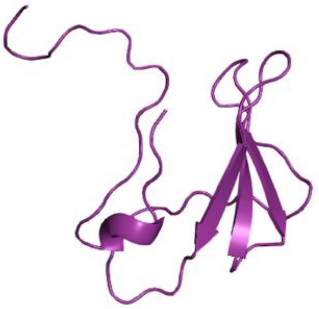

(30) M.TH16.EuGene 00079311

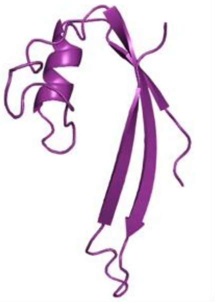

(31) M.TH16.EuGene_00099371

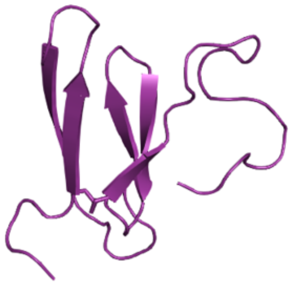

(32) M.TH16.EuGene_00101881

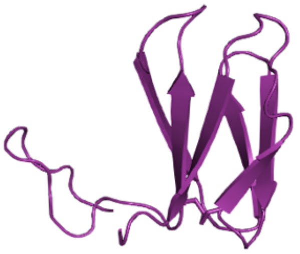

(33) M.TH16.EuGene_00106621

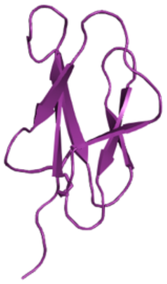

(34) M.TH16.EuGene_00120731

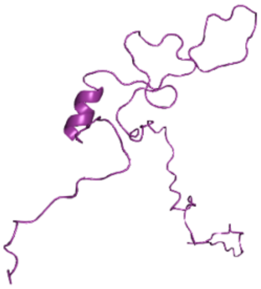

(35) M.TH16.EuGene_00124981

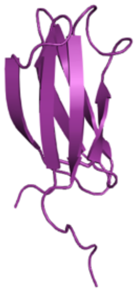

(36) M.TH16.EuGene_00127871

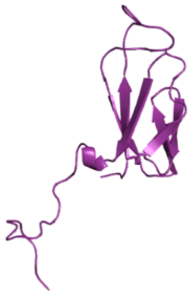

(37) M.TH16.EuGene_00134971

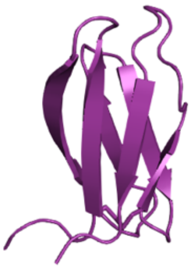

(38) M.TH16.EuGene_00135161

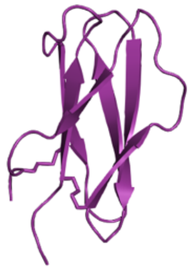

(39) MGG_00821

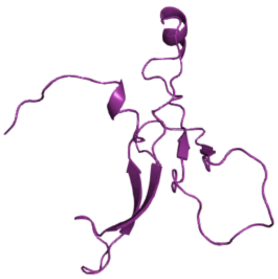

(40) MGG_04384

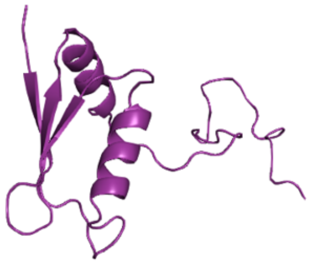

(41) MGG_08482

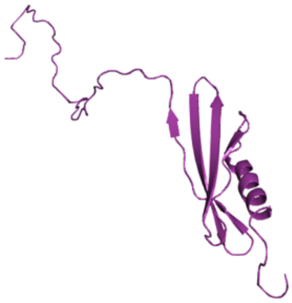

(42) MGG_08944

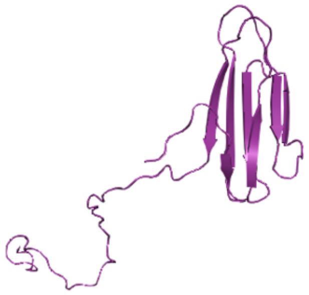

(43) MGG_10120

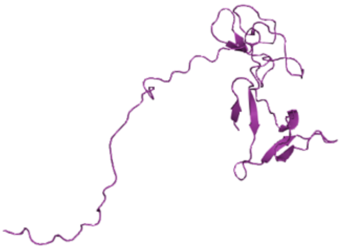

(44) MGG_14793

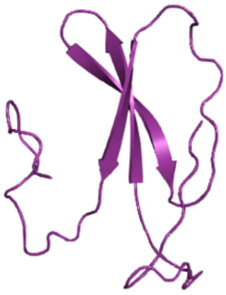

(45) MGG_14834

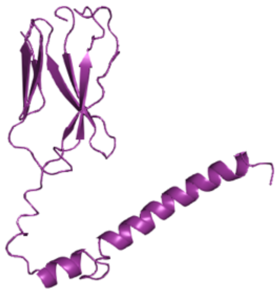

(46) MGG_15207

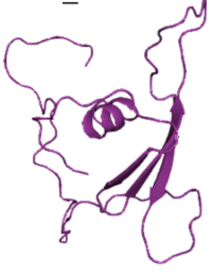

(47) MGG_15459

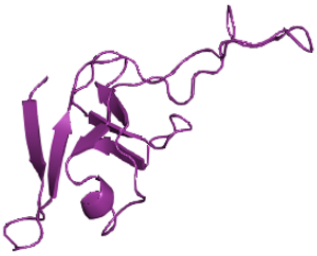

(48) MGG_16058

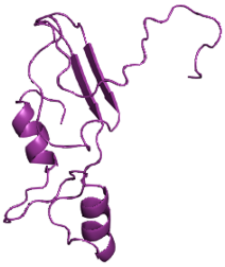

(49) MGG_16113

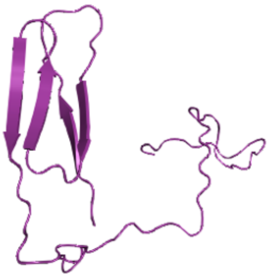

(50) MGG_16175

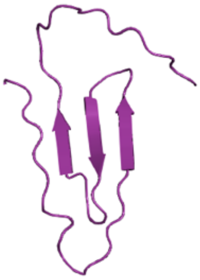

(51) MGG_16619

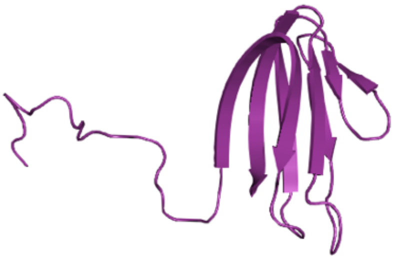

(52) MGG_17132

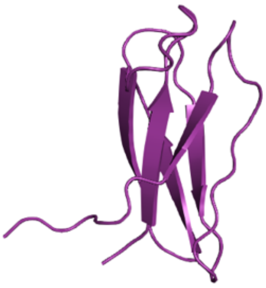

(53) MGG_17255

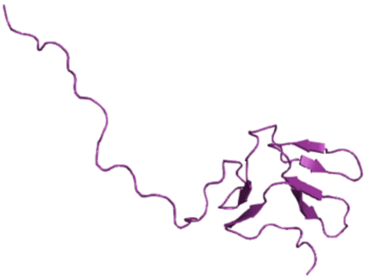

(54) MGG_18019

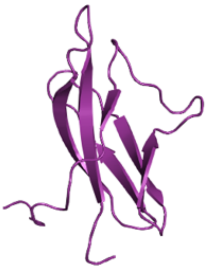

(55) MGG_18060

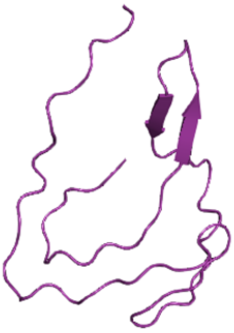

Fig. S7 Comparison of models predicted for AvrA13 and AvrPm2 by Bauer S. *et al*. (2021) and Manser B. *et al.* (2021) (far left) and this study (middle) with respect to the RALPH structural family member BEC1054 (PDB ID 6FMB).

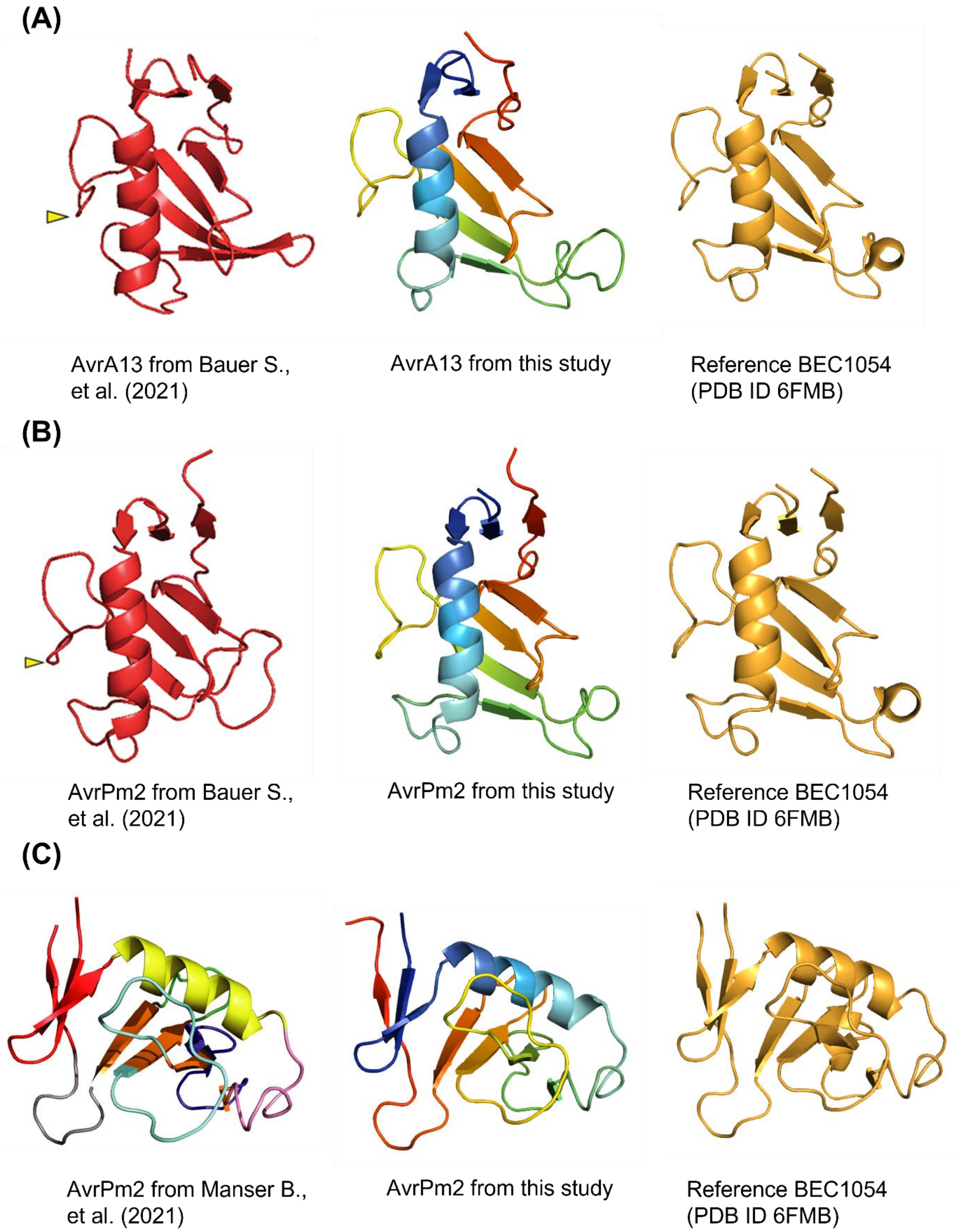

Fig. S8 Predicted models of phenotypically-validated effectors (shown in rainbow colours) with a RaptorX score below 50, superimposed onto their respective template structures (shown in grey).

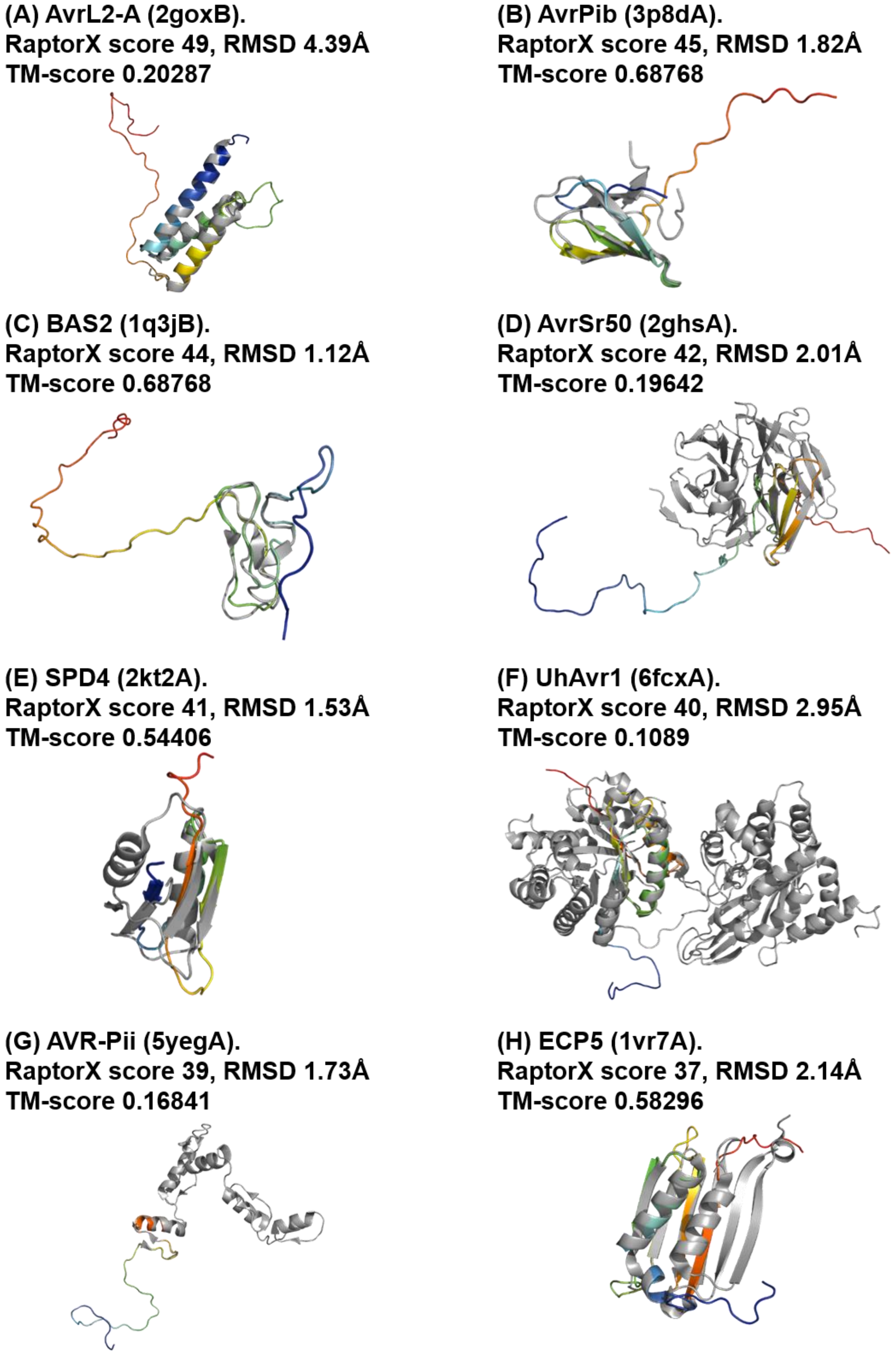

(I) Avr2 (6atyA).
RaptorX score 36, RMSD 2.03Å
TM-score 0.54893

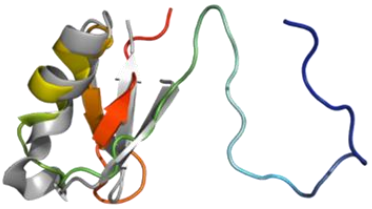

(J) ECP1 (1bnbA).
RaptorX score 35, RMSD 1.45Å
TM-score 0.60125

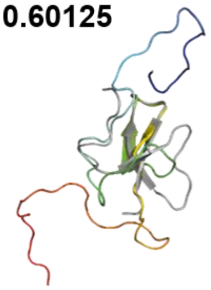

(K) Iug6 (2yilA).
RaptorX score 35, RMSD 1.73Å
TM-score 0.25163

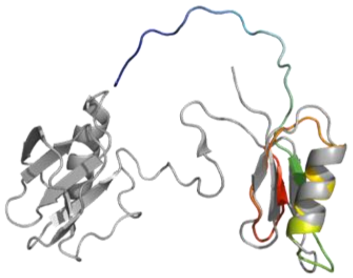

(L) SPD7 (5zngC).
RaptorX score 33, RMSD 3.59Å
TM-score 0.30259

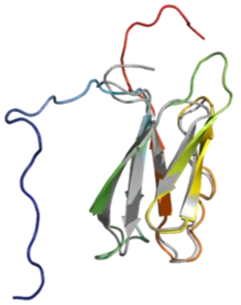

(M) MoHEG13 (3dzuA).
RaptorX score 31, RMSD 0.72Å
TM-score 0.07516

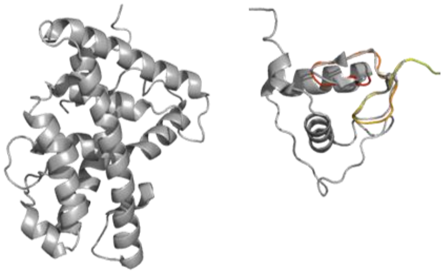

(N) PST_Pec6 (2mn1A).
RaptorX score 30, RMSD 0.95Å
TM-score 0.75574

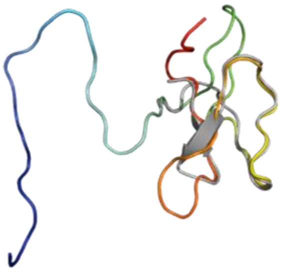

(O) Avr5 (5ji4A).
RaptorX score 29, RMSD 0.68Å
TM-score 0.90329

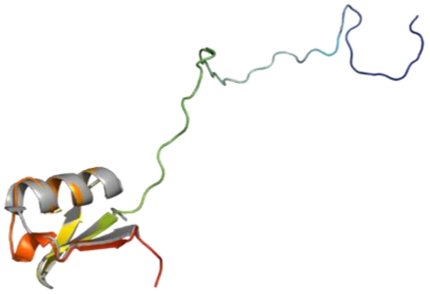

(P) Iug9 (3gedA).
RaptorX score 26, RMSD 2.13Å
TM-score 0.16718

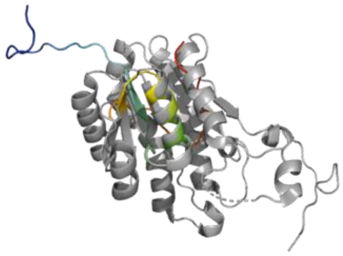

(Q) MISSP7 (1r7oA).
RaptorX score 15, RMSD 1.47Å
TM-score 0.12803

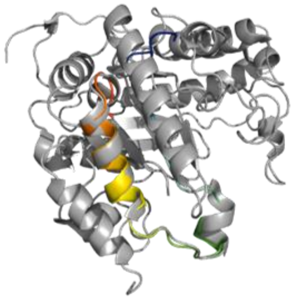

(R) MC69 (6i5OA).
RaptorX score 13, RMSD 3.69Å
TM-score 0.15567

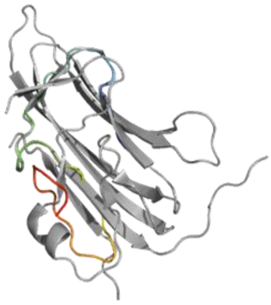

(S) SPD2 (3evsC).
RaptorX score 12, RMSD 2.37Å
TM-score 0.1994

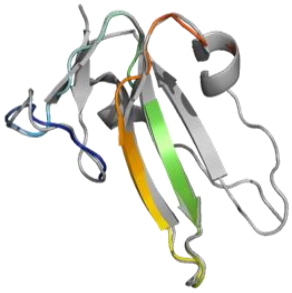

(T) Avr9 (6k4fU).
RaptorX score 10, RMSD 2.04Å
TM-score 0.23841

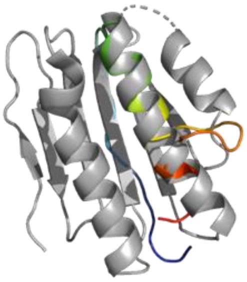

(U) SPD9 (5vjjA).
RaptorX score 6, RMSD 2.29Å
TM-score 0.25539

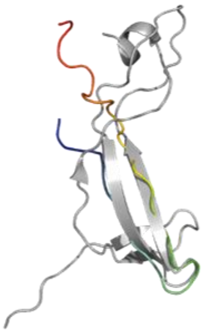

(V) BAS1 (3vjfA).
RaptorX score 4, RMSD 1.23Å
TM-score 0.16638

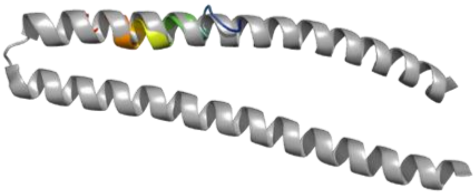

Fig. S9 RaptorX model of MAX-like effector candidate, M.BR20.EuGene_00106461 in purple, superimposed onto structural templates 2MYWA (A) and 2N37A (B), shown in yellow. The target-template sequence alignment and alignment RMSD are shown below each structure.

(A)

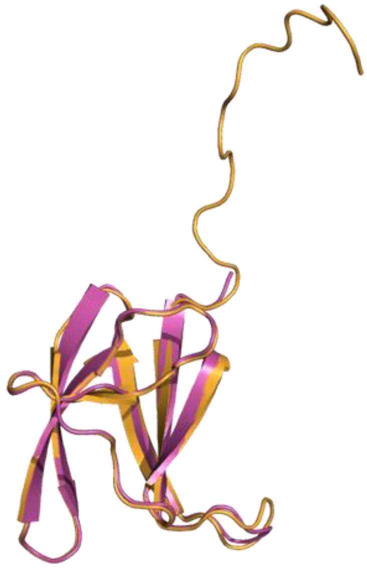

```
>M.BR20.EuGene_00106461
----------APEFCAYFTGSN-KSRRQVGVVRIGEIDTIIADGTELVVHAQDSRCQVILANGKPGPEWLSADPV
>AvrPia (PDB ID 2MYWA)
NLYFQGHMAAPARFCVYYDGHLPATRVLLMYVRIGTTATITARGHEFEVEAKDQNCKVILTNGKQAPDWLAAEPY
>Alignment RMSD
----------611000001245-772111100000000000000001000000000000000000000000000117
```

(B)

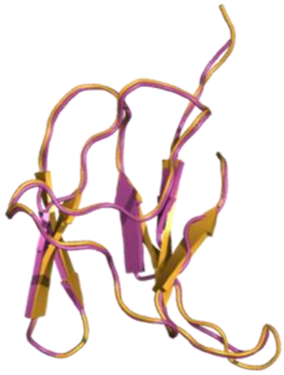

```
>M.BR20.EuGene_00106461
-APEFCAYFTGSNKS-RRQVGVVRIGEIDTIIADGTELVVHAQDSRCQVILANGKPGPEWLSADPV
>AvrPia (PDB ID 2N37A)
APARFCVYYDGHLPATRVLLMYVRIGTTATITARGHEFEVEAKDQNCKVILTNGKQAPDWLAAEPY
>Alignment RMSD
-61000000124577-722110000000000000001000000000000000000000000000117
```

Fig. S10 Target-template sequence alignment of ToxA-like candidate p2fl_ENH98532.1 modelled using RaptorX, SWISSMODEL and Phyre2.

```
>RaptorX model: p2fl_ENH98532
--KPLSLNARELSLDGRELSLDARSVLHKRQGSCVSISRPANNPSINNIGQIDITSVVNGG--PGMTWDLNNLVRVHVSREVDGSLSFDWTNTGSANRLIVTEWNSSSSTSGNGYVLLGSYGLPTGTGNICFPVGS-GQGRTWKMQLED
>Template ToxA (PDB ID 1ZLDA)
MG---------------------------------------------NIGQVDIDSVILGRPGAIGSWELNNFITIGLNRVNADTVRVNIRNTGRTNRLIITQWDNTVT-RGDVYELFGDYALIQGRGSFCLNIRSDTGRENWRMQLEN
>Alignment RMSD
-----------------------------------------------70000000000124--63210000000001011122211111000000000000000012346432100000000010000001113577620000000 1

>Phyre2 model: p2fl_ENH98532
KPLSLNARELSLDGRELSLDARSVLHKRQGSCVSISRPANNPSINNIGQIDITSVVNGGPGM--TWDLNNLVRVHVSREVDGSLSFDWTNTGSANRLIVTEWNSSSSTSGNGYVLLGSYGLPTGTGNICFPVGSGQGRT-WKMQLED
>Template ToxA (PDB ID 1ZLDA)
---------------------------------------------NIGQVDIDSVILGRPGAIGSWELNNFITIGLNRVNADTVRVNIRNTGRTNRLIITQW-DNTVTRGDVYELFGDYALIQGRGSFCLNIRSDTGRENWRMQLEN

>SWISSMODEL model: p2fl_ENH98532
KPLSLNARELSLDGRELSLDARSVLHKRQGSCVSISRPANNPSINNIGQIDITSVVNGGPGM--TWDLNNLVRVHVSREVDGSLSFDWTNTGSANRLIVTEWNSSSSTSGNGYVLLGSYGLPTGTGNICFPVGSGQGR-TWKMQLED
>Template ToxA (PDB ID 1ZLDA)
----------------------------QGSCMSITINPSRPSVNNIGQVDIDSVILGRPGAIGSWELNNFITIGLNRVNADTVRVNIRNTGRTNRLIITQWDNT-VTRGDVYELFGDYALIQGRGSFCLNIRSDTGRENWRMQLEN
```

Table S1 ToxA-like effector candidates obtained from in-house bioinformatics identification.

**ToxA-like effector candidates**	**Description**	**Species**
p2fk_EMD96331.1	hypothetical protein COCHEDRAFT_1019653	*Bipolaris maydis C5*
p2fl_ENH98532.1	hypothetical protein COCC4DRAFT_35205	*Bipolaris maydis ATCC 48331*
p2fn_EUC44184.1	hypothetical protein COCMIDRAFT_6505	*Bipolaris oryzae ATCC 44560*
p2fq_EUC36307.1	hypothetical protein COCCADRAFT_23897	*Bipolaris zeicola 26R13*
p2gb_RAQ98980.1	hypothetical protein DDE82_008711	*Stemphylium lycopersici*
p09v_mRNA10419	_joined_ _translated_	
p0dd_mRNA2255	_joined_ _translated_	
p22r_EXK24251.1	hypothetical protein FOMG_19011	*Fusarium oxysporum f. sp. melonis 26406*
p2g0_EFQ93895.1	hypothetical protein PTT_08588	*Pyrenophora teres f. teres 01*
p2g7_PZD04407.1	hydrophobin-like protein	
p2g8_PWO20795.1	hypothetical protein PtrARCrB10_10699	*Pyrenophora triticirepentis*
p2g9_EDU49735.1	predicted protein	
p2g3_PZD24241.1	hypothetical protein A1F96_09523	*Pyrenophora triticirepentis*
p2g4_PZD32416.1	hypothetical protein A1F97_09587	*Pyrenophora triticirepentis*
p2g5_PZD46046.1	hypothetical protein A1F99_08479	*Pyrenophora triticirepentis*
p2g6_PWO08528.1	hypothetical protein PtrM4_10348	*Pyrenophora triticirepentis*
p05g_mRNA17320	_joined_ _translated_	
p05m_mRNA12409	_joined_ _translated_	
p1bd_mRNA1147	_joined_ _translated_	
p05n_mRNA11205	_joined_ _translated_	
p05k_mRNA3392	_joined_ _translated_	
p05d_mRNA9122	_joined_ _translated_	
p05e_mRNA13670	_joined_ _translated_	
p05c_mRNA16607	_joined_ _translated_	
p1ap_mRNA3793	_joined_ _translated_	
p1b1_EXF72942.1	hypothetical protein CFIO01_04648	*Colletotrichum fioriniae PJ7*
p1bd_mRNA10016	_joined_ _translated_	
p1bo_mRNA4951	_joined_ _translated_	
p1bi_OBR06575.1	EC13 protein	
p09v_mRNA9195	_joined_ _translated_	
p0de_mRNA10272	_joined_ _translated_	
p2g2_PZC93680.1	hypothetical protein A1F95_07125	*Pyrenophora triticirepentis*
p2g1_PZD05769.1	hypothetical protein A1F94_09312	*Pyrenophora triticirepentis*

Table S2 MAX-effector candidates identified by PSI-BLAST in the genomes of the *M. oryzae* isolates 70-15 and TH16 and the *M. grisea* isolate BR29. Taken from Table S3 (de Guillen *et al*., 2015).

geneID	localization	AVR-Pia*	AVR1-CO39*	ToxB*
M_BR29_EuGene_00004921	scaffold00001	9,0E-12	-	6,0E-12
M_BR29_EuGene_00041131	scaffold00007	-	9,0E-06	-
M_BR29_EuGene_00043011	scaffold00008	-	9,0E-06	-
M_BR29_EuGene_00060181	scaffold00013	-	4,0E-08	-
M_BR29_EuGene_00081821	scaffold00023	-	3,0E-07	-
M_BR29_EuGene_00082031	scaffold00023	-	1,0E-05	-
M_BR29_EuGene_00085071	scaffold00025	-	-	3,0E-15
M_BR29_EuGene_00087671	scaffold00027	4,0E-13	4,0E-12	8,0E-11
M_BR29_EuGene_00088411	scaffold00027	-	-	5,0E-12
M_BR29_EuGene_00091361	scaffold00030	3,0E-12	-	-
M_BR29_EuGene_00091681	scaffold00031	-	2,0E-05	-
M_BR29_EuGene_00095641	scaffold00035	-	3,0E-13	5,0E-13
M_BR29_EuGene_00106461	scaffold00049	7,0E-23	-	-
M_BR29_EuGene_00107481	scaffold00051	-	2,0E-06	-
M_BR29_EuGene_00112111	scaffold00059	-	6,0E-09	-
M_BR29_EuGene_00113041	scaffold00061	-	7,0E-08	-
M_BR29_EuGene_00118801	scaffold00076	-	2,0E-05	-
M_BR29_EuGene_00119491	scaffold00079	-	3,0E-22	-
M_BR29_EuGene_00119511	scaffold00079	-	9,0E-17	-
M_BR29_EuGene_00121691	scaffold00087	-	1,0E-13	-
M_BR29_EuGene_00125811	scaffold00145	-	7,0E-08	-
M_BR29_EuGene_00126081	scaffold00163	-	-	2,0E-20
MGG_00821	Chromosome_8.5	-	6,0E-14	-
MGG_04384	Chromosome_8.2	-	2,0E-04	-
MGG_08482	Chromosome_8.4	-	1,0E-06	-
MGG_08944	Chromosome_8.2	-	-	5,0E-09
MGG_10120	Chromosome_8.4	-	5,0E-04	-
MGG_14793	Chromosome_8.2	4,0E-19	3,0E-06	9,0E-15
MGG_14834	Chromosome_8.4	-	2,0E-05	-
MGG_15207	Chromosome_8.3	-	5,0E-08	-
MGG_15459	Chromosome_8.1	-	8,0E-08	-
MGG_16058	Chromosome_8.1	-	1,0E-05	-
MGG_16113	Chromosome_8.1	-	3,0E-09	-
MGG_16175	Chromosome_8.1	-	5,0E-08	-
MGG_16619	Chromosome_8.3	-	3,0E-06	-
MGG_17132	Chromosome_8.4	2,0E-06	7,0E-04	9,0E-10
MGG_17255	Chromosome_8.4	-	1,0E-06	-
MGG_18019	Chromosome_8.7	-	7,0E-05	8,0E-11
MGG_18060	Chromosome_8.7	5,0E-14	5,0E-14	4,0E-06
M_TH16_EuGene_00000541	scaffold00001	-	1,0E-14	-
M_TH16_EuGene_00027191	scaffold00004	4,0E-10	-	-
M_TH16_EuGene_00027411	scaffold00004	4,0E-12	-	-
M_TH16_EuGene_00034081	scaffold00004	-	3,0E-08	-
M_TH16_EuGene_00040131	scaffold00005	2,0E-25	-	-
M_TH16_EuGene_00045871	scaffold00007	-	3,0E-12	-
M_TH16_EuGene_00079081	scaffold00016	6,0E-10	-	-
M_TH16_EuGene_00079311	scaffold00016	-	2,0E-08	-
M_TH16_EuGene_00099371	scaffold00026	-	3,0E-08	-
M_TH16_EuGene_00101881	scaffold00028	-	2,0E-05	2,0E-08
M_TH16_EuGene_00106621	scaffold00033	-	-	5,0E-14
M_TH16_EuGene_00120731	scaffold00052	3,0E-06	-	-
M_TH16_EuGene_00124981	scaffold00063	-	7,0E-11	-
M_TH16_EuGene_00127871	scaffold00072	-	2,0E-21	-
M_TH16_EuGene_00134971	scaffold00110	-	9,0E-14	1,0E-14
M_TH16_EuGene_00135161	scaffold00112	-	-	2,0E-19

Table S3 List of the top five templates used in the modelling of MAX-like effector candidates with RaptorX, sorted by alphabetical order. Their respective *p-value*, RaptorX score, uGDT/GDT, uSeqID/SeqID and PDB ID of the reference template are shown. MAX structures used within the top five templates are highlighted in bold.

**MAX-like effector candidates**	**Rank**	**Template PDB ID**	***p-value***	**RaptorX score**	**uGDT/ GDT**	**uSeqID/ SeqID**
M.BR29.EuGene_00004921	1	2wcoA	2.30E-02	19	28/28	10/10
	2	4ij3C	3.50E-02	17	27/27	9/9
	3	1hn0A	2.70E-02	18	30/30	7/7
	4	1rw9A	3.10E-02	18	25/25	13/13
	5	4rzcA	2.40E-02	19	25/26	12/12
M.BR29.EuGene_00041131	1	6czjA	7.30E-04	26	27/27	19/19
	2	4rhzA	2.10E-03	23	23/24	11/11
	3	2fkgA	2.00E-02	17	29/29	15/15
	4	6czgA	1.90E-03	23	25/25	11/11
	5	1fj1E	1.90E-02	17	28/28	15/15
M.BR29.EuGene_00043011	1	4c2mA	3.60E-03	25	29/25	6/5
	2	2bmbA	2.20E-02	20	24/20	7/6
	3	3lr5A	2.30E-02	19	24/20	5/4
	4	3g65A	6.90E-03	23	21/18	3/3
	5	2ya1A	3.10E-02	18	25/21	9/8
M.BR29.EuGene_00060181	1	6euhA	1.30E-02	22	40/55	8/11
	2	6eufA	6.10E-03	24	36/50	9/13
	3	3vsfA	4.10E-03	26	34/48	10/14
	4	3nqhA	2.00E-02	20	36/50	4/6
	5	1uypA	2.00E-02	20	32/44	8/11
M.BR29.EuGene_00081821	1	**2mm2A**	1.20E-02	25	40/36	9/8
	2	**5a6wC**	1.80E-02	24	34/30	10/9
	3	4i2yA	1.80E-02	24	32/28	12/11
	4	3qphA	1.60E-02	24	27/24	11/10
	5	1hkfA	3.70E-02	21	32/28	5/4
M.BR29.EuGene_00082031	1	1uy1A	1.00E-02	24	28/30	3/3
	2	2vvzA	1.50E-02	23	27/29	9/10
	3	4yz1A	9.80E-03	25	28/31	7/8
	4	1w0oA	5.30E-03	27	26/28	8/9
	5	5xxzA	8.30E-03	25	21/23	7/8
M.BR29.EuGene_00085071	1	**2mm2A**	7.20E-04	28	41/71	20/34
	2	1hm2A	1.80E-02	17	24/41	7/12
	3	4ohvA	1.90E-02	17	25/42	6/10
	4	3jx8A	2.40E-02	16	24/41	10/17
	5	3n91A	4.10E-02	15	23/39	5/9
M.BR29.EuGene_00087671	1	2axcA	3.90E-03	22	31/48	10/15

	2	6bbtA	9.70E-03	19	31/47	9/14
	3	3b2mA	1.10E-02	19	30/45	7/11
	4	5lgdA	1.10E-02	19	27/41	7/11
	5	4ohvA	8.90E-03	20	26/40	4/6
M.BR29.EuGene_00088411	1	**2mm2A**	2.30E-03	26	37/49	10/13
	2	4p71A	3.00E-02	17	25/32	13/17
	3	6aphA	4.50E-02	16	30/39	5/6
	4	1a6xA	2.20E-02	18	23/30	10/13
	5	5y9hA	3.50E-02	16	23/30	4/5
M.BR29.EuGene_00091361	1	3prxB	1.90E-02	26	27/47	7/12
	2	5xxzA	1.50E-02	27	29/50	3/5
	3	**2mm2A**	2.30E-02	25	27/48	12/21
	4	3h7jA	1.20E-02	28	28/49	4/7
	5	5zbfA	2.10E-02	25	27/47	6/11
M.BR29.EuGene_00091681	1	4r4xA	2.30E-02	16	33/34	12/13
	2	5x04A	5.00E-02	14	24/25	5/5
	3	1jb7B	5.60E-02	13	23/24	12/13
	4	3b7kA	7.70E-02	12	22/23	5/5
	5	3odtA	7.80E-02	12	21/22	5/5
M.BR29.EuGene_00095641	1	5izdA	3.40E-02	27	28/26	10/10
	2	2obdA	1.40E-02	31	26/25	6/6
	3	4m4dA	1.50E-02	31	25/24	10/10
	4	1bp1A	1.80E-02	30	26/25	7/7
	5	3ue8A	2.10E-02	29	25/24	3/3
M.BR29.EuGene_00106461	1	**2mywA**	3.70E-05	56	58/90	29/45
	2	**2n37A**	4.60E-04	43	58/90	29/45
	3	2lqmA	2.50E-02	24	25/39	11/17
	4	2o14A	1.60E-02	26	28/43	6/9
	5	1yllA	1.60E-02	26	23/37	7/11
M.BR29.EuGene_00107481	1	**2mm2A**	3.20E-02	15	30/38	4/5
	2	**5a6wC**	1.60E-02	16	24/30	8/10
	3	3bmzA	1.90E-02	16	23/30	6/8
	4	4q63A	2.40E-02	15	22/28	8/10
	5	5nldA	2.50E-02	15	26/33	3/4
M.BR29.EuGene_00112111	1	2n59A	6.60E-03	22	23/28	4/5
	2	5iwzA	2.50E-02	17	24/29	8/10
	3	4gucA	3.40E-02	16	23/27	12/14
	4	1vl4A	1.40E-02	19	20/25	9/11
	5	5woeA	5.20E-02	14	26/31	6/7
M.BR29.EuGene_00113041	1	1lshA	1.10E-02	23	33/32	11/11
	2	4ga7A	1.80E-02	21	29/28	13/13
	3	5ek8A	1.90E-02	21	30/30	8/8
	4	3e8vA	6.50E-03	25	29/29	6/6
	5	3ne4A	1.50E-02	22	28/28	8/8

M.BR29.EuGene_00118801	1	6e7kC	2.00E-02	14	27/38	9/13
	2	1kbaA	4.00E-02	12	24/34	6/9
	3	4wa0A	2.40E-03	18	18/26	5/7
	4	5opgA	4.30E-03	17	18/26	6/9
	5	6bbtA	1.50E-02	14	19/27	9/13
M.BR29.EuGene_00119491	1	**2myvA**	1.00E-02	21	34/44	19/25
	2	**5zngC**	3.00E-02	18	30/39	19/25
	3	5y6tA	4.50E-02	17	27/36	11/14
	4	3dt5A	4.50E-02	17	23/31	9/12
	5	5mekA	3.50E-02	18	22/29	7/9
M.BR29.EuGene_00119511	1	**2myvA**	4.30E-04	28	41/51	18/23
	2	**5zngC**	1.10E-03	25	39/49	18/23
	3	1dabA	5.00E-03	21	35/43	9/11
	4	5is8A	2.80E-03	22	33/41	12/15
	5	3b3jA	4.00E-03	21	33/41	13/16
M.BR29.EuGene_00121691	1	**5zngC**	8.70E-03	23	30/49	11/18
	2	**2myvA**	8.40E-03	23	29/48	11/18
	3	1b8mA	3.60E-02	17	27/44	7/11
	4	1gztA	2.20E-02	19	24/39	5/8
	5	1d2pA	2.50E-02	18	24/39	7/11
M.BR29.EuGene_00125811	1	3e8tA	2.80E-02	14	30/30	6/6
	2	**5zngC**	1.10E-02	17	26/26	9/9
	3	**2myvA**	1.20E-02	17	26/26	12/12
	4	4he4A	3.50E-02	14	30/29	10/10
	5	5l7nA	1.10E-02	17	25/25	11/11
M.BR29.EuGene_00126081	1	**2mm2A**	5.90E-06	54	50/74	21/31
	2	5j44A	1.50E-02	22	30/44	11/16
	3	2wlcA	1.60E-02	21	27/39	10/15
	4	3laqU	1.40E-02	22	29/42	8/12
	5	3jx8A	2.40E-02	20	25/37	12/18
M.TH16.EuGene_00000541	1	2w5fA	1.30E-02	18	25/26	12/12
	2	2uv8G	2.80E-02	16	25/26	8/8
	3	3c7gA	5.80E-03	21	24/25	7/7
	4	4n0rA	2.20E-02	17	24/24	12/12
	5	5tgnA	2.90E-02	16	22/23	14/14
M.TH16.EuGene_00027191	1	5cyyA	5.30E-03	22	24/36	8/12
	2	1h54A	6.00E-03	21	27/41	7/11
	3	**2mm2A**	8.10E-03	20	26/40	10/15
	4	4tsdA	5.50E-03	21	23/35	7/11
	5	3j31Q	2.40E-03	24	21/32	11/17
M.TH16.EuGene_00027411	1	6fbsA	1.30E-02	22	33/39	11/13
	2	6bn8A	5.60E-03	26	32/37	10/12
	3	4a08A	2.20E-03	29	26/31	9/10
	4	4xrtA	2.60E-03	29	26/31	7/8

	5	2znwA	1.00E-02	23	28/32	9/10
M.TH16.EuGene_00034081	1	2qc5A	9.50E-03	19	25/34	8/11
	2	1npeA	1.50E-02	18	24/32	7/10
	3	2xe4A	8.60E-03	20	20/28	7/10
	4	3nvnB	1.60E-02	18	20/27	5/7
	5	3u0sA	4.70E-03	22	21/29	5/7
M.TH16.EuGene_00040131	1	**2mywA**	5.00E-05	54	53/79	29/43
	2	**2n37A**	7.20E-04	41	54/80	29/43
	3	5jctA	2.00E-02	24	30/45	12/18
	4	5yaxA	6.10E-03	30	27/40	8/12
	5	2bjqA	1.20E-02	27	28/42	11/16
M.TH16.EuGene_00045871	1	**5zngC**	5.30E-03	22	27/44	7/11
	2	**2myvA**	6.50E-03	21	27/43	7/11
	3	**5a6wC**	3.40E-02	15	25/40	9/15
	4	5uc2A	3.70E-02	15	25/40	7/11
	5	4b4pA	3.70E-02	15	24/38	7/11
M.TH16.EuGene_00079081	1	3u2gA	2.40E-02	21	33/44	10/13
	2	1smpI	9.90E-03	24	22/29	5/7
	3	3mswA	2.70E-02	20	28/37	7/9
	4	4qm8A	1.90E-02	21	28/38	8/11
	5	1jiwI	2.30E-02	21	26/34	5/7
M.TH16.EuGene_00079311	1	2uv8G	4.70E-02	15	31/43	11/15
	2	3vsfA	6.90E-03	21	24/34	10/14
	3	6eufA	7.00E-03	21	23/33	9/13
	4	1s98A	2.80E-02	16	24/34	7/10
	5	3wovA	1.70E-02	18	23/33	12/17
M.TH16.EuGene_00099371	1	5jowA	2.60E-02	16	29/42	12/17
	2	4csdA	1.80E-02	17	28/41	9/13
	3	6b9rA	4.20E-02	15	30/44	9/13
	4	**2myvA**	5.00E-03	21	24/35	8/12
	5	2wyoA	2.90E-02	16	25/36	5/7
M.TH16.EuGene_00101881	1	**2myvA**	3.60E-03	22	29/39	9/12
	2	**5zngC**	5.70E-03	20	28/37	9/12
	3	3vtmA	1.50E-02	17	26/35	6/8
	4	2e7dA	1.50E-02	17	26/35	6/8
	5	2qv3A	2.30E-03	23	20/27	11/15
M.TH16.EuGene_00106621	1	**2mm2A**	2.90E-03	26	47/74	19/30
	2	3jx8A	5.70E-03	23	22/34	4/6
	3	4peuA	7.40E-03	22	23/36	5/8
	4	4v17A	2.40E-02	18	27/43	7/11
	5	1yoxA	3.00E-02	17	25/40	9/14
M.TH16.EuGene_00120731	1	1e3mA	6.30E-02	14	27/22	11/9
	2	2d2xA	7.60E-02	14	22/19	10/8
	3	5m21B	1.50E-02	18	22/18	7/6

	4	4ehiA	3.90E-02	16	23/19	10/8
	5	4u4hA	3.50E-02	16	22/18	7/6
M.TH16.EuGene_00124981	1	**5a6wC**	1.60E-03	18	25/35	14/19
	2	3au7A	1.40E-02	13	26/37	14/19
	3	4eo0A	9.80E-03	14	27/37	9/13
	4	1xn6A	1.60E-02	13	24/34	3/4
	5	3amtA	1.40E-02	13	22/31	13/18
M.TH16.EuGene_00127871	1	**2myvA**	6.00E-03	18	34/38	20/23
	2	**5zngC**	4.70E-03	18	26/29	17/19
	3	1qwrA	2.20E-02	14	18/21	5/6
	4	5j4fA	2.80E-02	14	19/21	13/15
	5	1ftrA	2.60E-02	14	20/22	5/6
M.TH16.EuGene_00134971	1	**5zngC**	5.20E-03	21	25/42	11/18
	2	4lziA	2.20E-02	16	27/45	5/8
	3	4it7A	1.60E-02	17	26/44	7/12
	4	**5a6wC**	3.50E-02	15	23/38	11/18
	5	1b8mA	3.20E-02	15	27/44	9/15
M.TH16.EuGene_00135161	1	**2mm2A**	9.10E-06	53	50/74	20/29
	2	5j44A	1.70E-02	22	26/38	12/18
	3	5nxkA	2.00E-02	21	27/39	3/4
	4	2wlcA	2.30E-02	21	26/39	11/16
	5	4ohvA	9.40E-03	24	26/39	3/4
MGG_00821	1	3rwkX	2.80E-02	17	32/30	10/9
	2	6czjA	1.20E-02	19	23/22	14/13
	3	4eqvA	3.60E-02	16	31/30	8/8
	4	2vbeA	6.50E-03	21	27/25	13/12
	5	2ac1A	3.20E-02	16	28/26	12/11
MGG_04384	1	4erdA	3.90E-02	18	21/22	6/6
	2	5uf5A	4.50E-02	18	23/24	10/10
	3	3vqtA	5.10E-02	17	20/20	13/13
	4	2xubA	6.80E-02	17	20/21	6/6
	5	6an0A	5.00E-02	18	20/20	11/11
MGG_08482	1	4r80A	9.00E-03	27	33/28	14/12
	2	5trvA	3.90E-02	22	30/25	9/8
	3	4n20A	3.90E-02	22	26/21	10/8
	4	5l33A	4.20E-02	21	26/21	11/9
	5	3ffyA	3.90E-02	22	24/20	11/9
MGG_08944	1	5azwA	1.80E-02	18	35/29	8/6
	2	**2mm2A**	1.00E-02	20	30/24	9/7
	3	6czjA	2.20E-03	25	29/23	15/12
	4	6czgA	6.30E-03	22	30/24	18/15
	5	3e5zA	5.20E-03	22	29/23	9/7
MGG_10120	1	1qxmA	1.80E-02	20	29/19	13/9
	2	3zssA	2.00E-02	19	27/18	10/7

	3	3winA	1.10E-02	22	28/18	8/5
	4	5gaqA	8.90E-03	22	31/21	14/9
	5	5ifeA	1.20E-02	21	26/17	10/7
MGG_14793	1	2xtlA	4.60E-03	19	30/39	10/13
	2	2d73A	6.50E-03	18	29/38	8/10
	3	2wv4A	8.90E-03	17	28/35	8/10
	4	**5a6wC**	5.00E-03	19	27/34	9/12
	5	4fqbB	2.40E-03	21	25/32	8/10
MGG_14834	1	5xcsA	2.00E-02	22	32/22	17/11
	2	3mkqA	2.80E-02	21	25/17	17/11
	3	1ee8A	3.90E-02	20	29/19	9/6
	4	5e1rA	2.50E-02	21	30/20	13/9
	5	3h4zA	2.80E-02	21	24/16	14/9
MGG_15207	1	3laeA	3.20E-02	15	28/24	5/4
	2	4yk8A	3.00E-02	16	27/24	7/6
	3	3dedA	4.40E-02	14	24/21	6/5
	4	3l5iA	3.60E-02	15	24/21	11/10
	5	4uf7A	2.90E-02	16	23/20	7/6
MGG_15459	1	1jiwI	2.20E-02	16	28/30	10/11
	2	2zuyA	1.70E-02	17	25/27	8/9
	3	2yb5A	1.50E-02	17	26/28	14/15
	4	3ge2A	1.10E-02	18	24/26	9/10
	5	4qniA	1.10E-02	18	24/26	9/10
MGG_16058	1	5wp4A	1.50E-02	24	34/32	11/10
	2	4jp0A	1.40E-02	24	40/37	14/13
	3	5x6rA	1.20E-02	25	40/37	8/7
	4	2wb7A	5.60E-03	28	36/33	13/12
	5	3kv4A	3.80E-03	29	36/33	9/8
MGG_16113	1	5n09A	3.00E-02	19	33/33	9/9
	2	5yeeA	8.10E-03	24	24/24	12/12
	3	5tz8A	2.20E-02	21	24/25	5/5
	4	5vbaA	3.60E-02	19	26/26	9/9
	5	4ye0A	2.70E-02	20	23/24	7/7
MGG_16175	1	4zktB	2.60E-02	17	28/49	8/14
	2	5hccC	4.30E-02	15	23/41	3/5
	3	4kh8A	4.90E-02	15	20/35	8/14
	4	4nw4A	4.40E-02	15	19/34	7/13
	5	5amsA	4.00E-02	16	21/37	1/2
MGG_16619	1	6d0gA	1.60E-02	23	30/33	7/8
	2	3abzA	2.40E-02	22	32/35	5/5
	3	1r6vA	2.50E-02	21	26/28	11/12
	4	6d0pA	1.20E-02	24	25/27	10/11
	5	3k4zA	1.60E-02	23	26/28	11/12
MGG_17132	1	**2mm2A**	1.00E-02	22	38/57	12/18

	2	3p53A	2.90E-02	18	29/43	9/13
	3	1jjuB	2.10E-02	20	27/40	4/6
	4	5n4aA	1.40E-02	21	27/40	8/12
	5	1ygyA	1.50E-02	21	25/38	9/13
MGG_17255	1	4le7A	2.40E-02	21	30/31	7/7
	2	5oo4A	1.80E-02	22	29/31	2/2
	3	3no2A	1.80E-02	22	26/28	9/9
	4	1srvA	2.40E-02	21	25/26	10/11
	5	5cyyA	1.40E-02	23	24/25	4/4
MGG_18019	1	**2mm2A**	2.30E-02	19	29/35	12/15
	2	3lxuX	4.00E-02	17	32/39	11/13
	3	3eifA	1.30E-02	22	26/31	13/16
	4	5xxzA	4.20E-02	17	28/34	12/15
	5	3hfqA	1.50E-02	21	29/36	5/6
MGG_18060	1	2axcA	4.20E-03	18	25/34	9/12
	2	3lycA	5.40E-03	18	21/29	5/7
	3	3b2mA	1.50E-02	15	24/32	5/7
	4	6bbtA	1.70E-02	15	22/30	5/7
	5	3petA	7.00E-03	17	19/25	2/3

Table S4 List of the top five templates used in the modelling of phenotypically-validated effector candidates used in this study (including effectors used in benchmarking), sorted by alphabetical order. Effector candidates marked with an asterisk (*) have more than one domain, modelled using a different template structure. Template PDB ID with entries modl# indicates no template structures available for modelling.

Effector name	Rank	Template PDB ID	PValue	RaptorX score	uGDT/GDT	uSeqID/SeqID
Ave1	1	4jcwA	2.40E-07	83	82/71	21/18
	2	2hczX	1.50E-07	85	80/69	21/18
	3	1n10A	2.70E-07	83	78/67	19/16
	4	2bh0A	5.00E-07	80	80/69	21/18
	5	4pmkA	9.30E-07	77	78/67	19/16
Avr1Co39	1	2myvA	9.40E-10	60	67/100	67/100
	2	5zngC	2.40E-08	52	60/89	61/91
Avr2	1	6atyA	5.00E-03	36	17/29	8/14
	2	2mn1A	1.00E-02	33	14/25	6/11
	3	2kvxA	1.00E-02	33	15/26	6/11
	4	1wqkA	1.40E-02	31	19/34	8/14
	5	6i31A	1.60E-02	30	19/34	6/11
Avr4	1	6bn0A	1.60E-11	64	66/56	79/68
Avr4e	1	modl1	0.00E+00	7	57/56	0/0
	2	modl2	0.00E+00	7	57/56	0/0
	3	modl3	0.00E+00	7	57/56	0/0
	4	modl4	0.00E+00	8	58/57	0/0
	5	modl5	0.00E+00	8	58/58	0/0

Avr5	1	5ji4A	5.90E-03	29	15/17	8/9
	2	1mynA	1.40E-02	25	15/17	5/6
	3	1rjiA	1.60E-02	25	12/14	6/7
	4	1nd9A	1.70E-02	25	14/17	2/2
	5	2e2fA	1.90E-02	24	13/15	9/11
Avr9	1	6k4fU	3.00E-02	10	21/53	9/23
	2	4i6jB	5.00E-03	14	15/38	8/20
	4	5e5qA	1.30E-02	12	17/43	8/20
	3	3odnA	1.90E-02	11	16/40	11/28
	5	2jveA	4.20E-02	10	17/43	6/15
Avra1	1	modl1	0.00E+00	6	52/56	0/0
	2	modl2	0.00E+00	6	51/56	0/0
	3	modl3	0.00E+00	6	52/57	0/0
	4	modl4	0.00E+00	6	53/58	0/0
	5	modl5	0.00E+00	6	52/56	0/0
AvrA13	1	6fmbA	1.80E-07	74	64/64	13/13
	2	1rtuA	4.90E-04	41	60/60	13/13
	3	1rdsA	1.10E-04	47	54/54	13/13
	4	3whoA	1.20E-04	47	54/54	8/8
	5	1b2mA	1.30E-04	47	51/51	14/14
AvrL2-A	1	2goxB	6.20E-03	49	24/21	6/5
	2	5w7gB	6.30E-03	48	25/22	8/7
	3	3fblA	6.40E-03	48	23/20	8/7
	4	3crjA	7.40E-03	47	23/20	4/4
	5	4l3iA	7.90E-03	47	26/23	12/11
AvrL567A	1	2opcA	1.60E-13	100	119/93	115/91
	2	2qvtA	4.30E-13	97	115/91	103/81
AvrLm1*	1	4uu4A	3.70E-03	56	20/31	8/13
	2	2c2iA	4.50E-03	54	20/32	7/11
	3	1e54A	4.90E-03	54	21/33	2/3
	4	5g38A	5.10E-03	53	25/40	6/9
	5	5dyqA	5.30E-03	53	23/35	0/0
AvrLm1	1	2zxqA	5.00E-03	42	19/30	4/6
	2	6dhxA	5.20E-03	41	22/35	5/8
	3	4ci1B	5.50E-03	41	22/36	5/8
	4	5fqdB	5.50E-03	41	21/34	5/8
	5	4akrA	5.50E-03	41	27/43	12/19
AvrLm11	1	modl1	0.00E+00	6	43/57	0/0
	2	modl2	0.00E+00	6	43/57	0/0
	3	modl3	0.00E+00	6	43/56	0/0
	4	modl4	0.00E+00	6	43/56	0/0
	5	modl5	0.00E+00	6	44/58	0/0
AvrLm4-7	1	4fprA	2.80E-10	98	107/87	118/97
AvrLm6	1	modl1	0.00E+00	6	75/60	0/0
	2	modl2	0.00E+00	6	73/59	0/0
	3	modl3	0.00E+00	6	75/60	0/0

	4	modl4	0.00E+00	6	71/57	0/0
	5	modl5	0.00E+00	6	73/59	0/0
AvrM	1	4bjmA	1.70E-06	243	204/71	226/79
AvrM14	1	3u53A	1.20E-06	105	96/65	28/19
	2	1vc8A	1.00E-06	106	93/64	28/19
	3	3i7uA	6.20E-07	110	90/62	23/16
	4	5gg5A	1.00E-06	106	89/61	24/16
	5	3gz8A	9.90E-07	106	85/58	43752
AvrP123	1	5vjjA	7.80E-13	65	65/69	48/51
AvrPi9	1	modl1	0.00E+00	5	41/56	0/0
	2	modl2	0.00E+00	5	41/55	0/0
	3	modl3	0.00E+00	5	41/55	0/0
	4	modl4	0.00E+00	5	40/55	0/0
Avr-Pia	1	5jhjA	2.70E-06	62	65/99	66/100
	2	2n37A	4.40E-05	50	67/101	64/97
AvrPib	1	3p8dA	2.50E-03	45	17/33	06/12
	2	4w9xA	2.80E-03	44	17/33	3/6
	3	2bujA	2.90E-03	44	19/36	0/0
	4	5kzfA	3.30E-03	43	20/38	2/4
	5	3ossC	3.50E-03	43	19/36	4/8
AVR-Pii	1	5yegA	2.10E-03	39	13/25	7/14
	2	5t0uA	2.50E-03	38	13/26	7/14
	3	5undA	3.20E-03	37	15/29	7/14
	4	5k5iA	3.60E-03	36	13/26	7/14
	5	2jvxA	3.60E-03	36	13/26	7/14
AvrPik	1	5a6wC	1.60E-12	76	84/91	83/90
Avr-Pita1	1	1eb6A	1.10E-15	246	131/63	44/21
	2	1g12A	3.10E-14	223	108/52	29/14
	3	2x3aA	5.70E-13	202	128/62	34/16
AvrPizt	1	2lw6A	1.20E-10	66	77/86	80/89
AvrPm2	1	6fmbA	4.00E-08	82	69/70	40/41
AvrSr50	1	2ghsA	2.20E-03	42	23/21	3/2
	2	2zzeA	2.80E-03	40	22/20	13/12
	3	2n93A	3.40E-03	39	22/20	7/6
	4	6c1zA	4.10E-03	38	24/22	8/7
	5	3r05A	4.30E-03	38	18/16	7/6
Bas1	1	3vjfA	1.00E-02	4	14/85	3/18
	2	6acxA	1.60E-04	7	13/78	8/47
	3	4a17M	2.40E-03	5	13/79	2/12
	4	1abzA	5.10E-03	4	14/84	3/18
	5	4dv8A	2.10E-02	4	14/84	3/18
Bas107	1	modl1	0.00E+00	5	67/59	0/0
	2	modl3	0.00E+00	5	67/60	0/0
	3	modl4	0.00E+00	5	67/59	0/0
	4	modl5	0.00E+00	5	67/59	0/0
	5	modl6	0.00E+00	5	66/58	0/0

Bas162	1	modl1	0.00E+00	7	54/53	0/0
	2	modl2	0.00E+00	7	53/52	0/0
	3	modl3	0.00E+00	7	53/52	0/0
	5	modl5	0.00E+00	7	53/52	0/0
	4	modl4	0.00E+00	7	52/52	0/0
Bas2	1	1q3jA	5.60E-03	44	14/17	7/8
	2	1m2sA	7.60E-03	42	13/16	9/11
	3	2msoA	7.70E-03	42	14/17	6/7
	4	4mpiA	8.10E-03	42	41974	8/10
	5	6atlA	8.10E-03	42	25/31	12/14
Bas3	1	1ti5A	4.30E-04	59	25/27	12/13
	2	6atyA	5.50E-04	58	22/24	9/10
	3	1fjnA	9.00E-04	55	27/30	9/10
	4	1wmtA	9.40E-04	54	23/25	9/10
	5	6atmC	1.00E-03	54	27/30	7/8
Bas4	1	4gvbB	4.90E-03	52	19/23	14/17
	2	4gvbA	7.50E-03	48	19/24	11/14
	3	2cb0A	8.30E-03	48	19/23	4/5
	4	2o57A	9.20E-03	47	19/23	4/5
	5	3tn7A	9.40E-03	47	18/22	2/2
Cgfl	1	4k90A	1.30E-14	337	360/92	282/72
Cmu1	1	6fpgC	1.19E-08	234	235/87	262/97
Ecp1	1	1bnbA	1.40E-02	35	15/19	5/6
	2	5k3qA	1.40E-02	34	16/21	5/6
	3	3t5nA	1.50E-02	34	15/20	0/0
	4	5il7A	1.80E-02	33	14/18	3/4
	5	3ctaA	1.90E-02	33	15/20	2/3
Ecp2*	1	6pzkA	3.30E-03	57	19/21	3/3
	2	3tjoA	4.20E-03	55	25/27	5/5
	3	5y2dA	4.90E-03	53	21/23	3/3
	4	3nziA	5.20E-03	53	24/26	5/5
	5	4ic6A	5.40E-03	53	25/27	1/1
Ecp2	1	modl1	0.00E+00	2	35/69	0/0
	2	modl2	0.00E+00	2	35/69	0/0
	3	modl3	0.00E+00	2	35/69	0/0
	4	modl4	0.00E+00	2	35/68	0/0
	5	modl5	0.00E+00	2	36/71	0/0
Ecp4	1	modl1	0.00E+00	6	56/56	0/0
	2	modl2	0.00E+00	6	56/56	0/0
	3	modl3	0.00E+00	6	57/57	0/0
	4	modl4	0.00E+00	6	58/58	0/0
	5	modl5	0.00E+00	6	55/55	0/0
Ecp5	1	1vr7A	8.30E-03	37	18/18	9/9
	2	2fjzA	1.00E-02	36	20/21	6/6
	3	2axkA	1.10E-02	36	14/14	7/7
	4	1mynA	1.30E-02	35	13/13	5/5

	5	2z3sA	1.60E-02	34	13/13	3/3
Ecp6	1	4b8vA	9.50E-12	158	160/76	189/90
eff1-1*	1	modl1	0.00E+00	2	47/69	0/0
	2	modl2	0.00E+00	2	46/68	0/0
	3	modl3	0.00E+00	2	45/66	0/0
	4	modl4	0.00E+00	2	45/66	0/0
	5	modl5	0.00E+00	2	45/67	0/0
eff1-1	1	modl1	0.00E+00	5	102/64	0/0
	2	modl2	0.00E+00	5	102/64	0/0
	3	modl3	0.00E+00	5	102/64	0/0
	4	modl4	0.00E+00	5	103/65	0/0
	5	modl5	0.00E+00	5	103/65	0/0
FGL1	1	3ngmA	3.10E-12	325	275/82	293/87
Iug9	1	3gedA	1.70E-02	26	14/25	6/10
	2	3l46A	1.70E-02	26	16/27	3/5
	3	3ewmA	1.90E-02	26	13/23	2/3
	4	2hy5C	2.00E-02	26	14/25	6/10
	5	1x1eA	2.00E-02	26	14/24	3/5
lug6	1	2yilA	1.40E-02	35	15/28	5/9
	2	1bgkA	1.50E-02	35	18/34	7/13
	3	5l74A	1.60E-02	34	45261	6/11
	4	6atyA	1.90E-02	34	15/29	6/11
	5	1wmtA	2.20E-02	33	14/26	5/9
MC69	1	6i5OA	8.05E-03	13	25/66	4/11
	2	1rj8A	1.30E-02	12	25/65	4/11
	3	4mxwB	1.50E-02	12	25/65	8/21
	4	5cirA	1.80E-02	11	23/61	6/16
	5	4ht1T	1.20E-02	12	22/59	3/8
MiSSP7	1	1r7oA	1.20E-01	15	22/47	4/9
	2	4yn5A	1.20E-01	16	23/48	5/11
	3	4u94A	1.00E-01	16	21/45	7/15
	4	6co7A	1.50E-01	15	21/44	7/15
	5	5kdgA	1.10E-01	16	22/46	5/11
MoCDIP1	1	3gq7A	2.70E-12	226	171/51	47/14
	2	2uveA	6.10E-11	201	173/52	45/13
	3	5zruA	7.60E-12	218	168/50	46/14
	4	3jurA	3.30E-11	206	163/49	43/13
	5	3sucA	3.50E-12	224	161/48	48/14
MoCDIP2*	1	4y7sA	7.70E-05	97	52/78	15/23
MoCDIP2	1	6sgwB	3.40E-02	30	30/103	4/14
	2	5a1sA	4.30E-02	28	29/101	2/7
	3	2jlnA	5.10E-02	27	28/95	4/14
	4	2x1lA	4.80E-02	28	21/73	3/10
	5	4kppA	3.40E-02	30	21/74	3/10
MoCDIP3	1	modl1	0.00E+00	6	54/58	0/0
	2	modl3	0.00E+00	6	54/58	0/0

	3	modl4	0.00E+00	6	54/57	0/0
	4	modl5	0.00E+00	6	55/58	0/0
	5	modl6	0.00E+00	6	54/57	0/0
MoCDIP4*	1	4b5qA	1.40E-12	148	172/80	85/40
	2	3eiiA	7.70E-13	150	163/76	69/32
	3	4d7uA	5.10E-13	152	156/73	74/34
MoCDIP4	1	4bmfA	2.90E-04	17	32/52	20/32
	2	4qi6A	6.20E-05	20	27/43	19/31
	3	4qi4A	9.20E-05	19	26/42	19/31
MoHEG13	1	3dzuA	2.10E-02	31	14/28	3/6
	2	1vraB	2.40E-02	30	15/30	1/2
	3	1erpA	2.70E-02	29	12/24	5/10
	4	3dzuD	2.80E-02	29	13/26	3/6
	5	1fjnA	2.90E-02	29	13/25	5/10
Msp1	1	3m3gA	5.00E-10	114	106/89	81/68
NEP1	1	3gnuP	6.85E-19	204	174/77	89/39
NIP1	1	1kg1A	2.63E-09	53	55/88	60/97
NIP2	1	3tljA	3.80E-03	52	21/22	9/10
	2	3gnlA	4.60E-03	51	21/23	5/5
	3	3tmaA	4.80E-03	51	21/22	8/7
	4	4x4nA	5.50E-03	50	19/20	6/6
	5	2yxdA	5.50E-03	49	20/22	4/4
NIP3	1	modl1	0.00E+00	9	50/51	0/0
	2	modl2	0.00E+00	9	49/50	0/0
	3	modl3	0.00E+00	9	49/50	0/0
	4	modl4	0.00E+00	9	49/50	0/0
	5	modl5	0.00E+00	9	49/51	0/0
Pep1	1	modl1	0.00E+00	7	58/59	0/0
	2	modl2	0.00E+00	7	56/57	0/0
	3	modl3	0.00E+00	7	59/59	0/0
	4	modl4	0.00E+00	7	57/58	0/0
	5	modl5	0.00E+00	8	57/57	0/0
PevD1	1	5xmzA	2.40E-09	125	110/81	122/89
PGTG_08638	1	5cwhA	7.00E-04	124	35/16	21/10
	2	5cwmA	8.20E-04	122	38/18	29/13
	3	5cwpA	8.40E-04	122	41/19	15/7
	4	5cwbA	8.40E-04	122	34/16	27/13
	5	5cwjA	1.10E-03	117	36/17	15/7
PpEC23	1	modl1	0.00E+00	7	152/56	0/0
	2	modl2	0.00E+00	8	150/55	0/0
	3	modl3	0.00E+00	8	151/55	0/0
	4	modl4	0.00E+00	8	146/53	0/0
	5	modl5	0.00E+00	8	150/55	0/0
PST_Pec6	1	2mn1A	2.20E-02	30	16/24	7/11
	2	2kvxA	2.30E-02	30	12/14	7/11
	3	1afpA	3.00E-02	28	9/14	10/15

	4	4mi0A	3.30E-02	28	15/23	11/17
	5	4w2rA	3.30E-02	28	15/23	10/15
PstSCR1	1	modl1	0.00E+00	4	61/66	0/0
	2	modl2	0.00E+00	4	61/66	0/0
	3	modl3	0.00E+00	4	61/67	0/0
	4	modl4	0.00E+00	4	61/67	0/0
	5	modl5	0.00E+00	4	62/67	0/0
Pwl1	1	modl1	0.00E+00	6	70/56	0/0
	2	modl2	0.00E+00	6	69/55	0/0
	3	modl3	0.00E+00	6	69/55	0/0
	4	modl4	0.00E+00	6	70/56	0/0
	5	modl5	0.00E+00	6	69/55	0/0
RTP1	1	5v6iA	6.10E-05	77	52/40	16/12
	2	4ndsA	1.20E-04	72	34/26	13/10
	3	1kptA	1.40E-04	71	25/19	9/7
	4	6greA	4.70E-04	62	25/19	10/8
	5	6grfA	7.30E-04	59	33/26	13/10
See1	1	modl1	0.00E+00	6	77/56	0/0
	2	modl2	0.00E+00	6	77/56	0/0
	5	modl5	0.00E+00	6	77/56	0/0
	4	modl4	0.00E+00	6	75/56	0/0
	3	modl3	0.00E+00	6	77/57	0/0
Six1	1	modl1	0.00E+00	8	158/60	0/0
	2	modl2	0.00E+00	8	158/60	0/0
	3	modl3	0.00E+00	8	153/58	0/0
	4	modl4	0.00E+00	8	156/59	0/0
	5	modl5	0.00E+00	8	160/61	0/0
Six2	1	modl1	0.00E+00	8	115/54	0/0
	2	modl2	0.00E+00	8	119/56	0/0
	3	modl3	0.00E+00	8	115/54	0/0
	4	modl4	0.00E+00	8	116/55	0/0
	5	modl5	0.00E+00	8	115/54	0/0
Six3	1	5od4A	2.50E-09	96	120/84	123/85
Six4	1	modl1	0.00E+00	6	134/59	0/0
	2	modl2	0.00E+00	6	133/59	0/0
	3	modl3	0.00E+00	6	131/58	0/0
	4	modl4	0.00E+00	6	133/59	0/0
	5	modl5	0.00E+00	6	135/60	0/0
Six5	1	modl1	0.00E+00	3	74/73	0/0
	2	modl2	0.00E+00	3	75/73	0/0
	3	modl3	0.00E+00	3	75/74	0/0
	4	modl4	0.00E+00	3	74/73	0/0
	5	modl5	0.00E+00	3	73/72	0/0
Six6*	1	1a9xA	5.10E-03	41	15/24	7/11
	2	3bg5A	5.90E-03	40	17/27	3/5
	3	6eojA	6.00E-03	40	21/32	3/5

	4	4k86A	6.90E-03	39	18/29	5/8
	5	5dotA	7.00E-03	39	19/30	2/3
Six6	1	2e3xA	4.80E-03	51	9/17	4/8
	2	3zxbA	5.30E-03	50	14/26	5/9
	3	1ethB	6.20E-03	49	16/31	5/9
	4	1i8xA	6.30E-03	49	19/35	5/9
	5	5hccD	6.50E-03	49	12/22	5/9
Six6	1	modl1	0.00E+00	6	44/53	0/0
	2	modl2	0.00E+00	6	43/53	0/0
	3	modl3	0.00E+00	6	44/54	0/0
	4	modl4	0.00E+00	6	44/54	0/0
	5	modl5	0.00E+00	6	44/53	0/0
Six7	1	modl1	0.00E+00	7	114/56	0/0
	2	modl2	0.00E+00	7	117/58	0/0
	3	modl3	0.00E+00	7	118/59	0/0
	4	modl4	0.00E+00	7	119/59	0/0
	5	modl5	0.00E+00	7	115/57	0/0
Six8*	1	1es2A	6.00E-03	61	21/35	3/5
	2	1w80A	8.50E-03	58	22/36	5/8
	3	1qh3A	8.60E-03	58	21/35	4/7
	4	3hs8A	1.10E-02	56	23/38	5/8
	5	5bmnA	1.10E-02	56	21/34	5/8
Six8	1	modl1	0.00E+00	4	40/63	0/0
	2	modl2	0.00E+00	4	38/61	0/0
	3	modl3	0.00E+00	4	40/63	0/0
	4	modl4	0.00E+00	4	39/62	0/0
	5	modl5	0.00E+00	4	39/62	0/0
SPD10	1	modl1	0.00E+00	5	35/56	0/0
	2	modl2	0.00E+00	5	35/56	0/0
	3	modl3	0.00E+00	5	35/56	0/0
	4	modl4	0.00E+00	5	37/58	0/0
	5	modl5	0.00E+00	5	35/56	0/0
SPD2	1	3evsC	9.80E-03	12	22/50	6/13
	2	5wb7E	2.00E-02	10	25/55	6/13
	3	1bnbA	9.20E-03	12	21/46	6/13
	4	2k3gA	9.70E-03	12	20/43	6/13
	5	1kj6A	1.00E-02	12	24/53	8/18
SPD4	1	2kt2A	1.10E-02	41	18/35	9/17
	2	6mrsA	1.10E-02	41	15/30	4/8
	3	3lvjC	1.10E-02	41	17/32	5/10
	4	1t43A	1.20E-02	41	16/31	3/6
	5	1dusA	1.30E-02	40	16/32	4/8
SPD7	1	5zngC	3.50E-03	33	21/23	10/11
	2	2ml7A	6.40E-03	30	14/15	1/1
	3	1cnnA	8.30E-03	29	44176	4/4
	4	2mm2A	9.20E-03	29	22/24	6/6

	5	2myvA	9.30E-03	29	21/22	7/8
SPD9	1	5vjjA	2.40E-02	6	19/56	24/8
	2	5edvA	7.50E-02	5	19/56	44169
	3	3v64C	5.50E-02	5	18/54	45505
	4	3rlcA	2.30E-02	6	17/50	43252
	5	5gyyG	3.90E-02	6	14/44	44169
SPD10	1	modl1	0.00E+00	5	35/56	0/0
	2	modl2	0.00E+00	5	35/56	0/0
	3	modl3	0.00E+00	5	35/56	0/0
	4	modl5	0.00E+00	5	37/58	0/0
	5	modl5	0.00E+00	5	35/56	0/0
Tin2	1	1s8nA	1.40E-03	65	31/17	15/8
	2	2chgA	2.30E-03	61	38/21	9/17
	3	4d6xA	2.50E-03	60	30/17	12/21
	4	3grcA	2.70E-03	60	30/17	11/20
	5	1a3aA	2.80E-03	59	25/14	11/20
Tox1	1	modl1	0.00E+00	6	60/60	0/0
	2	modl2	0.00E+00	6	59/59	0/0
	3	modl3	0.00E+00	6	59/59	0/0
	4	modl4	0.00E+00	6	59/59	0/0
	5	modl5	0.00E+00	6	58/58	0/0
Tox3	1	1cidA	3.00E-03	31	17/20	9/11
	2	3u0sA	3.20E-03	31	21/25	4/3
	3	4leeA	3.50E-03	30	21/24	12/14
	4	4zkqA	4.80E-03	29	24/28	9/8
	5	2l4vA	5.00E-03	29	20/23	6/5
ToxA	1	1zldA	3.20E-06	70	96/59	97/60
ToxB	1	2mm0	3.10E-06	60	64/100	64/100
UhAvr1	1	6fcxA	6.50E-03	40	22/26	7/8.0
	2	3ffyA	6.90E-03	39	21/25	11/9
	3	6hxjB	7.20E-03	39	22/26	5/4
	4	5gzsA	8.10E-03	38	22/26	4/3
	5	1oxbB	8.50E-03	38	19/22	12/10
VdSCP7	1	5cwfA	9.20E-03	61	22/23	10/10
	2	5hdtA	1.00E-02	60	23/24	7/7
	3	5c9sB	1.20E-02	58	21/22	12/13
	4	5zypA	1.30E-02	57	23/24	5/5
	5	5cwqA	1.30E-02	57	22/23	12/13
Zt6	1	1fusA	1.90E-09	102	98/82	66/55
	2	1trqA	2.80E-09	100	96/81	61/51

Table S5 Comparison of template-based modelling using RaptorX, SWISS-MODEL and Phyre2 of ToxA-like (A), MAX-like (B) and phenotypically-validated effector candidates (C). Template selection (PDB ID) and TM-score of the predicted models are displayed.

Table S5A ToxA-like candidates

**ToxA-like effector candidates**	**RaptorX**			**SWISS MODEL**			**Phyre2**		
	**Template PDB ID**	**RMSD**	**TM-score**	**Template PDB ID**	**RMSD**	**TM-score**	**Template PDB ID**	**RMSD**	**TM-score**
p05c_mRNA16607	1zldA	1.12	0.88709	1zleA	1.12	0.89181	3d12A	1.55	0.07707
p05d_mRNA9122	1zldA	0.92	0.89386	1zleA	1.00	0.90052	4q5yA	3.38	0.07543
p05e_mRNA13670	1zldA	1.00	0.88914	1zleA	0.90	0.90490	4q5yA	1.55	0.08218
p05g_mRNA17320	1zldA	0.87	0.90005	1zleA	1.06	0.89496	3d12A	5.11	0.13094
p05k_mRNA3392	1zldA	0.87	0.90005	1zleA	1.12	0.89181	3d12A	1.55	0.07707
p05m_mRNA12409	1zldA	0.91	0.90067	1zleA	1.12	0.89181	3d12A	1.55	0.07707
p05n_mRNA11205	1zldA	0.99	0.89235	1zleA	1.12	0.89181	3d12A	1.55	0.07771
p09v_mRNA10419	1zldA	1.26	0.87117	1bc8C	2.55	0.16959	4gs3A	0.00	0.31111
p09v_mRNA9195	1zldA	1.10	0.85520	3bc1B	2.87	0.17317	3onrI	0.61	0.48803
p0dd_mRNA2255	1zldA	1.01	0.89151	1zleA	1.29	0.87644	5nf8A	0.00	0.07547
p0de_mRNA10272	1zldA	0.84	0.66836	1zleA	0.88	0.61331	2xi5D	0.23	0.07050
p1ap_mRNA3793	1zldA	0.87	0.90312	1zleA	1.11	0.89401	4q5yA	1.55	0.08218
p1b1_EXF72942.1	1zldA	0.91	0.89719	1zleA	0.90	0.90490	4q5yA	1.55	0.08218
p1bd_mRNA10016	1zldA	0.79	0.90687	1zleA	0.90	0.90496	4q5yA	1.55	0.08218
p1bd_mRNA1147	1zldA	1.07	0.88942	1zleA	0.93	0.90126	4q5yA	1.55	0.08218
p1bi_OBR06575.1	1zldA	1.32	0.89174	1zleA	1.09	0.90632	1wfwa	1.05	0.58057
p1bo_mRNA4951	1zldA	0.82	0.90455	1zleA	1.04	0.89663	3d12A	1.49	0.08407
p22r_EXK24251.1	1zldA	1.16	0.88646	1zleA	1.00	0.88781	2gtia	0.66	0.13873
p2fk_EMD96331.1	1zldA	0.39	0.95090	1zleA	1.34	0.91668	1zldA	1.26	0.91087
p2fl_ENH98532 .1	1zldA	0.39	0.95141	1zleA	1.34	0.91668	1zldA	1.26	0.91087
p2fn_EUC44184.1	1zldA	1.19	0.90785	1zleA	1.57	0.88317	1zldA	1.12	0.77915
p2fq_EUC36307.1	1zldA	0.73	0.93061	1zleA	1.43	0.88198	3d37A	0.97	0.10535
p2g0_EFQ93895.1	1zldA	1.01	0.89152	1zleA	1.29	0.87644	5nf8A	0.00	0.07547

p2g1_PZD05769.1	1zldA	0.79	0.67131	1zleA	0.88	0.61331	2xi5D	0.23	0.07050
p2g2_PZC93680.1	1zldA	0.78	0.67124	1zleA	1.13	0.88886	1swga	0.68	0.15700
p2g3_PZD24241.1	1zldA	0.85	0.90474	1zleA	1.13	0.88886	1swga	0.68	0.15700
p2g4_PZD32416.1	1zldA	0.85	0.90474	1zleA	1.13	0.88886	1swga	0.68	0.15700
p2g5_PZD46046.1	1zldA	0.92	0.89835	1zleA	1.13	0.88886	1swga	0.68	0.15700
p2g6_PWO08528.1	1zldA	0.85	0.90474	1zleA	1.13	0.88886	1swga	0.68	0.15700
p2g7_PZD04407.1	1zldA	0.94	0.89685	1zleA	1.13	0.88886	1swga	0.68	0.15700
p2g8_PWO20795.1	1zldA	0.92	0.89835	1zleA	1.13	0.88886	1swga	0.68	0.15700
p2g9_EDU49735.1	1zldA	0.85	0.90474	1zleA	0.88	0.61331	2xi5D	0.23	0.07050
p2gb_RAQ98980.1	1zldA	0.89	0.91064	1zleA	1.04	0.88506	7jhyi	3.04	0.11098

Table S5B MAX-like candidates

MAX-like effector candidates	RaptorX			SWISSMODEL			Phyre2		
	Template PDB ID	RMSD	TM-score	Template PDB ID	RMSD	TM-score	Template PDB ID	RMSD	TM-score
M.BR29.EuGene_00004921	2wcoA	1.66	0.09860	3evvA	3.97	0.08492	1jiwi	1.01	0.03350
M.BR29.EuGene_00041131	6czjA	1.07	0.58545	5nbbA	3.33	0.17460	4opbA	1.53	0.08152
M.BR29.EuGene_00043011	4c2mA	4.99	0.04478	6ncqA	2.69	0.08762	3c6vB	0.62	0.25311
M.BR29.EuGene_00060181	3vsfA	1.37	0.10331	4ps6A	2.97	0.15394	1gqpa	0.00	0.06667
M.BR29.EuGene_00081821	2mm2A	0.72	0.92004	4r80A	0.33	0.74257	6pdyA	3.08	0.10637
M.BR29.EuGene_00082031	1w0oA	3.90	0.05838	5ikkA	3.05	0.05902	5j8jA	0.00	0.18033
M.BR29.EuGene_00085071	2mm2A	0.90	0.81525	2mm0A	1.18	0.83773	2mm0A	0.76	0.83949
M.BR29.EuGene_00087671	2axcA	2.20	0.23424	3ljyA	2.70	0.08828	2j2za	0.00	0.13889
M.BR29.EuGene_00088411	2mm2A	0.47	0.97549	2mm2A	0.13	0.75221	2r32A	1.72	0.21012
M.BR29.EuGene_00091361	3h7jA	0.68	0.25085	5z1vA	0.61	0.86577	5z1vB	0.45	0.88400
M.BR29.EuGene_00091681	4r4xA	1.51	0.14152	1evwC	1.35	0.30126	6cpdB	0.92	0.45646
M.BR29.EuGene_00095641	2obdA	1.58	0.18182	1j2jB	2.77	0.13625	4wt3A	1.04	0.17111
M.BR29.EuGene_00106461	2mywA	0.31	0.80389	2mywA	1.26	0.75242	2n37A	0.70	0.91942
M.BR29.EuGene_00107481	5a6wC	0.87	0.68531	2mm2A	1.20	0.68480	bjjA	0.00	0.17925
M.BR29.EuGene_00112111	2n59A	1.84	0.52902	3qk3A	0.80	0.22322	4pl6A	2.80	0.25363
M.BR29.EuGene_00113041	3e8vA	1.09	0.75567	2p52A	1.01	0.20350	3j81i	4.65	0.13312

M.BR29.EuGene_00118801	4wa0A	1.12	0.17542	4k5qA	0.33	0.16994	5apgA	0.00	0.08889
M.BR29.EuGene_00119491	2myvA	1.63	0.73718	5zngB	2.99	0.33481	2myvA	0.00	0.70886
M.BR29.EuGene_00119511	2myvA	1.13	0.74793	5zngB	2.61	0.31969	2myvA	0.00	0.79747
M.BR29.EuGene_00121691	5zngC	0.56	0.93489	1r6vA	0.06	0.05216	1jb3a	1.74	0.21206
M.BR29.EuGene_00125811	2myvA	0.50	0.82891	2o0iA	0.52	0.36861	1khiA	1.04	0.43526
M.BR29.EuGene_00126081	2mm2A	0.37	0.95371	6r5jA	0.06	0.99951	6r5jA	0.00	1.00000
M.TH16.EuGene_00000541	3c7gA	1.85	0.12762	6mjpC	2.59	0.07493	1e44b	1.99	0.10308
M.TH16.EuGene_00027191	3j31Q	0.83	0.26355	5z1vA	0.08	0.82060	5z1vB	0.41	0.76680
M.TH16.EuGene_00027411	4a08A	1.63	0.06316	3p6iA	0.77	0.29796	1vw4Q	3.55	0.06684
M.TH16.EuGene_00034081	3u0sA	1.17	0.17585	2it7A			1sqra	0.67	0.24537
M.TH16.EuGene_00040131	2mywA	0.24	0.81836	2n37A	2.22	0.71446	2n37A	0.47	0.95386
M.TH16.EuGene_00045871	5zngC	0.47	0.95616	2po8A			6e1lA	0.00	0.37500
M.TH16.EuGene_00079081	1smpI	1.16	0.61084	5g1kA	0.52	0.13048	6r9tA	0.00	0.01053
M.TH16.EuGene_00079311	3vsfA	2.69	0.11815	4ps6A	0.84	0.33397	1gqpa	0.00	0.06667
M.TH16.EuGene_00099371	2myvA	2.50	0.50920	2fjlA	0.09	0.21992	2ootA	1.59	0.03266
M.TH16.EuGene_00101881	2qv3A	1.19	0.14058	5zngB	3.84	0.24553	1k8wa	0.51	0.06866
M.TH16.EuGene_00106621	2mm2A	0.73	0.85801	2mm2A	1.07	0.80570	2mm0A	1.05	0.83790
M.TH16.EuGene_00120731	5m21B	3.57	0.17152	2atmA	1.04	0.20153	1ynha	0.97	0.08097
M.TH16.EuGene_00124981	5a6wC	0.63	0.73633	5apmB	1.76	0.18306	2xh3B	0.42	0.09430
M.TH16.EuGene_00127871	2myvA	0.80	0.79760	5zngB	2.46	0.23385	2myvA	0.00	0.44304
M.TH16.EuGene_00134971	5zngC	0.63	0.91542	2po8A			1ik9C	1.60	0.04456
M.TH16.EuGene_00135161	2mm2A	0.36	0.95411	6r5jA	0.06	0.99958	6r5jA	0.00	1.00000
MGG_00821	2vbeA	0.57	0.19744	6d9jA	2.60	0.10519	1jb0f	2.25	0.03729
MGG_04384	4erdA	1.79	0.48701	3hfnA	0.13	0.69831	4bjiA	1.05	0.07558
MGG_08482	4r80A	1.93	0.82190	2llxA	2.49	0.26535	4bjiA	3.06	0.05511
MGG_08944	6czjA	0.95	0.70716	2p4oA	0.21	0.25057	1gyva	0.50	0.23067
MGG_10120	5gaqA	2.75	0.23555	2mm2A	0.57	0.88416	6gf8B	0.24	0.25142
MGG_14793	4fqbB	1.46	0.41575	7nljB	3.68	0.22908	2hx6A	0.00	0.32026
MGG_14834	5xcsA	2.06	0.69473	1fcbA	0.35	0.07678	3fo8D	0.00	0.22263

MGG_15207	4uf7A	4.03	0.10481	5lzyA	3.36	0.08256	3esmA	0.00	0.13235
MGG_15459	3ge2A	1.48	0.69771	6asiA	0.63	0.12590	3biyA	3.38	0.07670
MGG_16058	3kv4A	2.07	0.19428	6qwjH	2.39	0.03779	1x4pA	0.00	0.45455
MGG_16113	5yeeA	1.69	0.45303	2gupA	0.73	0.11158	6e7kC	2.34	0.04796
MGG_16175	4zktB	2.68	0.04602	1xs0A	0.88	0.26623	2rqeA	1.39	0.41799
MGG_16619	6d0pA	1.50	0.26280	3s5dA	0.85	0.18242	5jk2I	0.38	0.10852
MGG_17132	2mm2A	0.90	0.86823	2mm0A	0.45	0.73380	1hx0a	1.50	0.05300
MGG_17255	5cyyA	1.28	0.23275	3gxwA	1.87	0.38839	5xfmD	0.88	0.09876
MGG_18019	3eifA	1.86	0.07889	5tcup	4.02	0.11777	2dexX	1.48	0.07174
MGG_18060	2axcA	2.78	0.20271	5z1vA	0.35	0.68300	3qrzC	1.91	0.24978

Table S5C Phenotypically-validated effector candidates.

EXP effector candidates	RaptorX			SWISSMODEL			Phyre2		
	Template PDB ID	RMSD	TM-score	Template PDB ID	RMSD	TM-score	Template PDB ID	RMSD	TM-score
Ave1	4jcwA	0.90	0.46504	2hczA	1.15	0.43719	3d30A	1.03	0.44469
Avr1Co39	2myvA	0.19	0.84511	5zngB	3.32	0.33362	2myvA	0.00	0.84810
Avr2	6atyA	2.03	0.54893	2mfjA	2.44	0.24139	4jprA	0.35	0.23413
Avr4	6bn0A	0.17	0.99718	6bn0A	0.07	0.97425	6bn0C	0.13	0.99833
AVR4E	NA	NA	NA	7npwA	0.09	0.10327	1c8ba	0.00	0.02500
Avr5	5ji4A	0.68	0.90329	6wf0B	3.17	0.05971	2qezC	2.66	0.06849
Avr9	6k4fU	2.04	0.23841	2msoA	2.27	0.24033	6kffG	1.51	0.02965
AVRa1	NA	NA	NA	3uc2A	2.66	0.16212	1saza	1.26	0.06166
AvrA13	6fmbA	0.47	0.95356	6fmbA	1.50	0.90910	4aygB	0.13	0.01459
AvrL2-A	2goxB	4.39	0.20287	6ft6j	4.91	0.19605	6cc4A	1.36	0.07428
AvrL567A	2opcA	0.12	0.99908	2opcA	0.09	0.99945	2qvtA	0.00	1.00000
AvrLm1	4uu4A	0.90	0.41112	7cyzA	0.10	0.16474	1vi7a	0.56	0.09610
AvrLm11	NA	NA	NA	1dnvA	0.13	0.06986	6wjeB	0.10	0.10312
AvrLm4-7	4fprA	0.37	0.93397	4fprA	0.10	0.93798	4fprC	0.01	0.93846
AvrLm6	NA	NA	NA	4l99A	0.42	0.09968	2jyvA	0.54	0.55951
AvrM	4bjmA	0.39	0.99636	4bjmA	1.72	0.87451	4bjnC	2.44	0.82599
Avrm14	3u53A	0.60	0.90964	1xsaA	2.64	0.68997	3fjyB	1.12	0.34818
AvrP123	5vjjA	0.89	0.94371	5vjjA	0.08	0.99938	5vjjA	0.00	0.93506
AvrPi9	NA	NA	NA	NA	NA	NA	6tmfW	1.86	0.06055
Avr-Pia	5jhjA	0.16	0.83321	5jhjA	1.19	0.73729	2n37A	0.00	1.00000
AvrPib	3p8dA	1.82	0.59263	5z1vA	0.45	0.90172	5z1vB	0.45	0.90176
AVR-Pii	5yegA	1.73	0.16841	4tpuA	2.08	0.10265	1yuzB	0.06	0.08499
AvrPik	5a6wC	4.24	0.31586	7bntC	3.98	0.30238	5a6wC	4.19	0.30165
AvrPita1	1eb6A	0.42	0.95981	2x3aA	1.80	0.49473	1eb6a	1.08	0.93328
AvrPizt	2lw6A	0.14	0.99805	2lw6A	0.08	0.99939	2lw6A	0.00	1.00000
AvrPm2	6fmbA	0.15	0.99823	6fmbA	0.12	0.98858	6fmbA	1.24	0.93491

AvrSr50	2ghsA	2.01	0.19642	7mqqA	0.07	0.97318	7mqqA	0.00	0.97345
BAS1	3vjfA	1.23	0.16638	1dvoA	0.10	0.26303	4hg1C	0.27	0.11292
BAS107	NA	NA	NA	6c23A	1.26	0.27179	6nq3F	2.97	0.05906
BAS162	NA	NA	NA	2lqvA	0.22	0.53347	1vqza	1.02	0.11126
BAS2	1q3jA	1.12	0.68768	2kraA	2.31	0.27484	1imtA	0.82	0.40410
BAS3	1ti5A	0.98	0.81542	2kbhA	1.71	0.49061	1ayja	2.29	0.45496
BAS4	4gvbB	2.44	0.62243	NA	NA	NA	7l2gA	0.00	0.30769
Cgfl	4k90A	0.06	0.99994	4k90A	0.14	0.99962	4k90A	0.10	0.99980
Cmu1	6fpgC	0.07	0.99987	6fpgA	0.65	0.96628	6fpgG	0.56	0.99170
ECP1	1bnbA	1.45	0.60125	7dabA	0.30	0.52525	1w4ha	0.76	0.43024
ECP2	6pzkA	3.27	0.04012	1ayyA	0.35	0.44677	1pwka	1.08	0.59649
ECP4	NA	NA	NA	5nnwA	1.34	0.10995	2l23A	0.82	0.29155
ECP5	1vr7A	2.14	0.58296	7ak7A	0.15	0.31137	5a6wC	2.12	0.12694
Ecp6	4b8vA	0.10	0.99965	4b8vA	0.06	0.99987	4b9hA	0.00	1.00000
Eff1-1	NA	NA	NA	5t58A	0.19	0.23580	3ghgD	0.97	0.17721
FGL1	3ngmA	0.65	0.99313	3ngmA	0.13	0.98947	3ngmB	0.43	0.98206
Iug9	3gedA	2.13	0.16718	1d7qB	0.07	0.11271	5j4aA	0.00	0.08403
lug6	2yilA	1.73	0.25163	1cc1A	2.55	0.17149	4nx3D	1.28	0.17953
MC69	6i5OA	3.69	0.15567	NA	NA	NA	1zntA	1.34	0.60113
MISSP7	1r7oA	1.47	0.12803	6vmeA	0.12	0.47300	4j8cA	0.00	0.47727
MoCDIP1	3gq7A	1.96	0.49832	6k0sA	1.58	0.48166	2vjjA	1.22	0.44945
MoCDIP2	4y7sA	0.43	0.57875	4y7sA	0.21	0.63796	4y7sC	0.34	0.82289
MoCDIP3	NA	NA	NA	2mhyA	0.07	0.45578	1t61a	0.14	0.10307
MoCDIP4	4b5qA	0.35	0.95942	4b5qA	0.83	0.94162	4b5qA	1.18	0.93978
MoHEG13	3dzuA	0.72	0.07516	NA	NA	NA	2w2sA	0.00	0.04294
Msp1	3m3gA	0.14	0.99045	3m3gA	0.16	0.98196	3m3gA	0.37	0.97715
NEP1	3gnuP	0.26	0.98383	6qbeA	1.43	0.92395	3gnzP	0.54	0.97311
NIP1	1kg1A	0.24	0.99162	1kg1A	0.12	0.99779	1kg1a	0.00	1.00000
NIP2	3tljA	2.29	0.12595	1rcuA	0.19	0.24512	5btyA	0.73	0.17574
NIP3	NA	NA	NA	2cqeA	1.34	0.50896	4u4hA	0.57	0.11487
Pep1	NA	NA	NA	2cm0A	0.06	0.31304	2k0rA	0.00	0.32031
PevD1	5xmzA	0.14	0.99891	5xmzA	0.05	0.99163	5xmzA	0.00	1.00000
PGTG_08638	5cwhA	0.43	0.98591	4jrpB	0.78	0.19206	6b4gG	1.33	0.39313
PpEC23	NA	NA	NA	7bz2E	2.97	0.13718	7nxdB	6.10	0.11956
PST_Pec6	2mn1A	0.95	0.75574	5ix5A	0.25	0.46702	3t38B	0.21	0.06589
Pst2	NA	NA	NA	NA	NA	NA	NA	NA	NA
PstSCR1	NA	NA	NA	6xiwB	2.85	0.01960	6qesA	0.00	0.17500
Pw11	NA	NA	NA	2okaA	2.96	0.22761	3ulxA	0.00	0.09589
RTP1	5v6iA	0.72	0.93581	5ckqA	4.34	0.12271	1szna	0.69	0.06898
See1	NA	NA	NA	2c5rA	0.55	0.87415	1yhnB	0.53	0.07026
SIX1	NA	NA	NA	NA	NA	NA	4zv4C	2.14	0.03305
SIX2	NA	NA	NA	5mdxB	5.06	0.16983	6hoxA	0.66	0.19103
SIX3	5od4A	0.12	0.99916	5od4A	0.07	0.98357	5od4A	0.07	0.99973
SIX4	NA	NA	NA	3o6rB	0.53	0.19376	3lyvF	0.58	0.15019
SIX5	NA	NA	NA	NA	NA	NA	4o7kA	1.23	0.28155
SIX6	2e3xA	4.19	0.08203	5ttaA	0.06	0.11960	6umlC	2.71	0.03042
SIX7	NA	NA	NA	1j83A	0.99	0.47030	7rozA	1.34	0.03896
SIX8	1es2A	2.36	0.14470	1h9cA	1.87	0.50595	2lsjB	0.29	0.09218
SPD10	NA	NA	NA	7figG	2.82	0.05390	1p0gA	0.00	0.78947

SPD2	3evsC	2.37	0.19940	3zxcA	0.13	0.40772	6qetA	0.00	0.21429
SPD4	2kt2A	1.53	0.54406	4v4t0	1.53	0.12707	1yuaa	0.00	0.14754
SPD7	5zngC	3.59	0.30259	3lt9A	1.50	0.21797	2nrzB	0.96	0.14347
SPD9	5vjjA	2.29	0.25539	NA	NA	NA	1sb2b	0.43	0.10500
Tin2	1s8nA	1.08	0.72602	3m7mA	1.62	0.29077	1v32a	1.80	0.10972
Tox1	NA	NA	NA	4b2uA	1.07	0.73156	2q3za	1.06	0.04525
Tox3	1cidA	1.37	0.36661	6wesA	0.06	0.99984	6wesA	0.00	1.00000
ToxA	1zldA	0.13	0.98892	1zleA	0.88	0.95926	1zldA	0.00	0.99020
ToxB	2mm0	0.16	0.99655	2mm0A	0.10	0.99874	2mm0A	0.00	1.00000
UhAvr1	6fcxA	2.95	0.10890	1hz9A	0.75	0.34646	16vpa	1.94	0.08140
VdSCP7	5cwfA	2.69	0.40282	6juqA	0.89	0.16649	4uzzB	0.29	0.11046
Zt6	1fusA	0.11	0.99915	1fusA	0.29	0.99537	1fusa	0.24	0.97792

NA: not available.

Table S6 Pfam v35.0 and InterProScan v5.57-90.0 classifications of RaptorX predicted models of phenotypically-validated effector candidates with RaptorX score above 50. Both programs were used with default parameters.

Effector name	Template name (PDB ID)	Pfam	InterProScan
Ave1	Expansin (4jcwA)	Lytic transglycolase	EG45-like domain containing protein
AvrLm1	LptA homologous periplasmic component of lipopolysaccharide transport device (4uu4A)	NA	NA
AvrM14	Human Ap4A hydrolase (3u53A)	NUDIX domain	NUDIX hydrolase domain
Avr-Pita	Deuterolysin (1eb6A)	Lysine-specific metallo-endopeptidase	Metallopeptidase, catalytic domain superfamily
Bas3	Plant defensin 1 (1ti5A)	NA	NA
Bas4	Virally encoded antifungal protein, KP6 heterodimer (4gvbB)	NA	NA
Cgfl	Extracellular metalloproteinase (4k90A)	Fungalysin metallopeptidase (M36), fungalysin/thermolysin propeptide motif	Metalloproteases, catalytic domain
Ecp2	Respiratory syncytial virus polymerase L protein (6pzkA)	Pathogen effector, putative necrosis-inducing factor	Pathogen effector, putative necrosis-inducing factor
MoCDIP1	Bacteriophage Phi29 gene product 12 N-terminal fragment (3gq7A)	Right-handed beta helix region	Pectin lyase-like
MoCDIP2	CFEM protein Csa2 (4y7sA)	CFEM domain	CFEM domain

MoCDIP4	Lytic polysaccharide monooxygenase GH61D (4b5qA)	Auxiliary Activity family 9 (formerly GH61), fungal cellulose binding domain	CBM1 (carbohydrate binding type-1) domain profile
Msp1	Sm1, elicitor of plant defense responses (3m3gA)	Cerato-platanin	RlpA-like domain superfamily
NIP2	tRNA:m2 G6 methyltransferase Trm14/TrmN (3tljA)	NA	NA
PGTG_ 08638	De novo designed helical repeat protein DHR14 (5cwhA)	Putative aromatic acid exporter, putative DNA-binding domain, RecG N-terminal helical domain, VHS domain, coenzyme F420 hydrogenase/ dehydrogenase	NA
RTP1	Glycan binding protein Y3 (5v6iA)	Pathogen effector, putative necrosis-inducing factor (shorten)	Polyampholyte
Six6	Viper venom metalloproteinase (2e3xA)	NA	NA
Six8	DD-transpeptidase/ penicillin binding protein (1es2A)	NA	NA
Tin2	Putative transcriptional antiterminator, Rv1626 (1s8nA)	NA	NA
VdSCP7	De novo designed helical repeat protein DHR8 (5cwfA)	Protein of unknown function (DUF3626), coiled-coil domain-containing protein 73	NA
Zt6	Ribonuclease f1 (1fusA)	Ribonuclease	Ribonuclease
